# The Electrophysiological Determinants of Corticospinal Motor Neuron Vulnerability in ALS

**DOI:** 10.3389/fnmol.2020.00073

**Published:** 2020-05-19

**Authors:** Javier H. Jara, Patrick L. Sheets, Maximiliano José Nigro, Mina Perić, Carolyn Brooks, Daniel B. Heller, Marco Martina, Pavle R. Andjus, P. Hande Ozdinler

**Affiliations:** ^1^Davee Department of Neurology and Clinical Neurological Sciences, Feinberg School of Medicine, Northwestern University, Chicago, IL, United States; ^2^Department of Physiology, Feinberg School of Medicine, Northwestern University, Chicago, IL, United States; ^3^Institute for Physiology and Biochemistry “Ivan Djaja”, Faculty of Biology, University of Belgrade, Belgrade, Serbia

**Keywords:** amyotrophic lateral sclerosis, corticospinal motor neurons, microcircuit, upper motor neurons, hereditary spastic paraplegia, primary lateral sclerosis, neuronal vulnerability

## Abstract

The brain is complex and heterogeneous. Even though numerous independent studies indicate cortical hyperexcitability as a potential contributor to amyotrophic lateral sclerosis (ALS) pathology, the mechanisms that are responsible for upper motor neuron (UMN) vulnerability remain elusive. To reveal the electrophysiological determinants of corticospinal motor neuron (CSMN, a.k.a UMN in mice) vulnerability, we investigated the motor cortex of hSOD1^G93A^ mice at P30 (postnatal day 30), a presymptomatic time point. Glutamate uncaging by laser scanning photostimulation (LSPS) revealed altered dynamics especially within the inhibitory circuitry and more specifically in L2/3 of the motor cortex, whereas the excitatory microcircuits were unchanged. Observed microcircuitry changes were specific to CSMN in the motor column. Electrophysiological evaluation of the intrinsic properties in response to the microcircuit changes, as well as the exon microarray expression profiles of CSMN isolated from hSOD1^G93A^ and healthy mice at P30, revealed the presence of a very dynamic set of events, ultimately directed to establish, maintain and retain the balance at this early stage. Also, the expression profile of key voltage-gated potassium and sodium channel subunits as well as of the inhibitory GABA receptor subunits and modulatory proteins began to suggest the challenges CSMN face at this early age. Since neurodegeneration is initiated when neurons can no longer maintain balance, the complex cellular events that occur at this critical time point help reveal how CSMN try to cope with the challenges of disease manifestation. This information is critically important for the proper modulation of UMNs and for developing effective treatment strategies.

## Introduction

Amyotrophic lateral sclerosis (ALS) is characterized by progressive degeneration of both upper motor neurons (UMNs) and spinal motoneurons (SMN; Brown and Robberecht, 2001; Bruijn et al., 2004). UMN has a unique ability to collect information from many different neuron types, including long-distance projection neurons, interneurons, and local circuitry neurons so that they can convey the cerebral cortex’s input to spinal cord targets (Lemon, 2008). The cortical component of the motor neuron circuitry is complex and understanding the intrinsic and extrinsic factors that contribute to UMN vulnerability is challenging. To emphasize their iimportant role in motor neuron circuitry and their projection from cortex to the spinal cord, we refer the UMN in mice the corticospinal motor neurons (CSMN). These neurons degenerate in both ALS patients (Genç et al., 2017) and in well characterized mouse models of the disease (Ozdinler et al., 2011; Joyce et al., 2015; Gautam et al., 2016, 2019; Fil et al., 2017).

Early cortical defects in ALS, especially in the form of high intracortical excitability, are well documented by various independent groups around the globe (Eisen et al., 1993; Prout and Eisen, 1994; Mills and Nithi, 1997; Ziemann et al., 1997; Vucic and Kiernan, 2006). Such defects are linked to spasticity and hyperreflexia, hallmarks of cortical pathology and ALS (Caramia et al., 2000; Vucic et al., 2008), and more recently cortical dysfunction and hyperexcitability were suggested to be used as an early detection marker for disease initiation (Geevasinga et al., 2016).

Since the overall activity of the neural circuitries is well maintained by the orchestrated events of both excitatory and inhibitory inputs, and that these events follow a dynamic profile over time, understanding what happens during distinct disease stages remains a challenge. Especially within the context of neuronal vulnerability, such dynamics need to be revealed with precision, so that appropriate modulations can be applied either to UMN directly or to the neurons/cells that modulate them. Therefore, understanding the extrinsic and intrinsic factors that contribute to UMN vulnerability is crucial.

Most of our understanding of ALS historically came from the hSOD1^G93A^ ALS mouse model (Gurney et al., 1994), as it had been the first mouse model generated for ALS with a phenotype and all pre-clinical testing had to include data obtained from this very mouse model, which also displayed progressive CSMN loss (Ozdinler et al., 2011). Even though CSMN numbers are not yet significantly reduced at P30, by P60 genes and canonical pathways related to apoptosis are observed in CSMN (Ozdinler Lab, unpublished results), their numbers become significantly lower than healthy controls as they fail to retain their health and integrity of their cytoarchitecture (Jara et al., 2012). We thus focused our attention on CSMN at P30, a critical early time in their neurodegeneration, and investigated both the extrinsic and the intrinsic contributors to their neuronal vulnerability.

To investigate the distinct contributions of extrinsic and intrinsic factors to CSMN vulnerability, we used a multifaceted approach. First, we investigated how CSMN functional connectivity is altered in cortical circuits using glutamate uncaging by laser scanning photostimulation (LSPS; Anderson et al., 2010). This approach allows the assessment of extrinsic contributors to CSMN function in both health and disease. To reveal intrinsic changes, we took two parallel approaches, one *via* electrophysiological recordings (Martina et al., 2007), and the other by performing exon microarray analysis to determine the changes in gene expression. Our results revealed how dynamic CSMN were at P30 and how they relentlessly tried to maintain their balance and electrophysiological properties by changing the expression of selected ion channel subunits that play a pivotal role for voltage-gated ion conductance. Even though an overall look may not reveal any significant change in intrinsic membrane properties at this critical age, CSMN were very active. Revealing the details of events, which at times may appear counteractive or opposing each other’s impact, helped us understand the extent of problems UMNs are faced with and what solutions they developed to counteract them. Since neuronal vulnerability is initiated when a neuron fails to maintain its homeostasis, being able to see the components of the turmoil just before losing balance is pivotal. Defining these molecular determinants of early stages of neurodegeneration especially in CSMN is what we try to achieve in this study, because this information will help identify targets for future modulations so that effective and long-term treatment strategies can be developed.

## Materials and Methods

### Animals

All procedures were approved by the Northwestern University Animal Care and Use Committee and conformed to the standards of the National Institutes of Health. Wild type (WT), hSOD1^G93A^ transgenic ALS mice (Gurney et al., 1994) in the C57BL/6 background were obtained from Jackson laboratories. Also, the UCHL1-eGFP mice (generated by the Ozdinler Lab and made available at Jackson Laboratory, stock#. 022476; Yasvoina et al., 2013), as well as hSOD1G93A-UeGFP mice (generated in the Ozdinler Lab), were used in this study. Genotypes of mice were determined by PCR, as reported previously (Yasvoina et al., 2013). All animal procedures were approved by the Northwestern University Animal Care and Use Committee and conformed to the standards of the National Institutes of Health.

### Surgical Procedures

Surgeries were performed on mice that were deeply anesthetized with isoflurane, and placed into a stereotaxic platform. Micro-injections were performed using pulled-beveled glass micro-pipettes attached to a nanojector (Drummond Scientific, Broomall, PA, USA).

#### CSMN Retrograde Labeling

All surgeries were performed as previously described (Ozdinler et al., 2011). Briefly, a small laminectomy at the cervical spinal cord (C2-C3) level was performed to expose the spinal cord. CSMN were retrogradely labeled by injection of fluorescent microspheres (LumaFluor Inc., Naples, FL, USA; ~207 nl) into the corticospinal tract (CST) that lies within the dorsal funiculus (df) at 0.3 mm depth. Surgeries were performed at P21 and mice were sacrificed at P30 (*n* = 3). Only neurons that are retrogradely labeled were used for electrophysiological recording. All retrogradely labeled neurons were in layer 5 of the motor cortex.

#### Callosal Projection Neuron (CPN) Labeling

All surgeries were performed as previously described (Ozdinler et al., 2011). Briefly, a small unilateral craniotomy of ~3 mm^2^ to target the motor cortex (coordinates = +0.5 mm anterior-posterior; 1.5 mm mediolateral) was performed into the left hemisphere using a micro drill (Fine Science Tools, Foster City, CA, USA). Callosal Projection Neurons (CPN) were retrogradely labeled by four injections of fluorescent microspheres (LumaFluor Inc., Naples, FL, USA; a total of ~276 nl) within the craniotomy area. Surgeries were performed at P21 and mice were sacrificed at P30 (*n* = 3).

### FACS Purification of CSMN

Retrogradely labeled projection neurons were purified from WT and hSOD1^G93A^ mice based on their green fluorescence and the forward and side scatter characteristics of large projection neurons. Since retrograde labeling marks the corticospinal projection neurons within the motor cortex, at P30 the motor cortex was microdissected using a fluorescence-equipped dissecting microscope (SMZ-1500; Nikon) in the presence of cold dissociation medium (20 mM glucose, 0.8 mM kynurenic acid, 0.05 mM D(-)-2-amino-5-phosphonovaleric acid (AP5), 50 U/ml penicillin, 0.05 mg/ml streptomycin, 0.9 M Na_2_SO_4_ and 0.014 M MgCl_2_, pH = 7.35, and supplemented with B27) and enzymatically digested for 15–20 min (0.16 mg/liter L-cysteine HCl, 12 U/ml papain and 1U/ml DNAseI, pH = 7.35, prepared in dissociation medium) at 37°C. Enzymatic digestion was blocked by dissociation medium containing 10 mg/ml ovomucoid (Sigma) and 10 mg/ml bovine serum albumin (BSA), and cells were mechanically dissociated in trituration buffer (OPTIMEM, supplemented with 20 mM glucose, 0.4 mM kynurenic acid, 0.025 mM AP5, B27, and BSA). The supernatant was collected in trituration buffer for FACS purification using a FACSVantage SE Diva flow cytometer (Becton Dickinson). Retrograde labeling, dissociation and FACS purification yielded approximately 30,000 live CSMN per P30 mice. Each experiment was repeated at least 4–5 times and results were reproducible and comparable.

### Exon Microarray Analysis

Total RNA was extracted from FACS-purified retrogradely labeled CSMN using TRIZOL reagent (Invitrogen Life Technologies, Grand Island, NY, USA) with DNase digestion to prevent DNA contamination. RNA was reprecipitated in ethanol, integrity, and quantity was assessed (Agilent 2100 Bioanalyzer; Agilent Technologies, Palo Alto, CA, USA). Only high-quality RNA (OD260/280 ≥ 1.8, RIN > 7.0) were used. RNA isolated from two littermate pups with the same genotype was used, and total RNA was converted into biotinylated cRNA (Ambion Illumina RNA amplification kit; Ambion, Austin, TX, USA), it was quantified (ND-1000 Spectrophotometer; NanoDrop, Wilmington, DE, USA). Biotinylated cRNA (100 ng) was hybridized to Illumina MouseWG-6 v2 Expression BeadChips at 58°C overnight, according to the manufacturer’s instructions (Illumina Inc., San Diego, CA, USA), and were scanned using the Illumina BeadArray Reader. Data was generated through the Illumina BeadStudio software (Illumina Inc., San Diego, CA, USA) and probe signal intensities were quantile normalized and log-transformed. The exon microarray analyses were performed three independent times and the averages of exon readings were color-coded to improve visualization and data analyses. These procedures were performed by the Genomics Core Facility of Northwestern University.

### Preparation of Brain Slices

Coronal brain slices (300 μm) containing the motor cortex were prepared at postnatal day 29–30 as described (Anderson et al., 2010). Slices were cut in chilled choline-based solution (in mM: 110 choline chloride, 25 NaHCO_3_, 25 D-glucose, 11.6 sodium ascorbate, 7 MgSO_4_, 3.1 sodium pyruvate, 2.5 KCl, 1.25 NaH_2_PO_4_, and 0.5 CaCl_2_), allowed to recover in 35°C ACSF (in mM: 127 NaCl, 25 NaHCO_3_, 25 D-glucose, 2.5 KCl, 1 MgCl_2_, 2 CaCl_2_, and 1.25 NaH_2_PO_4_) for 30 min and maintained at 21–22°C thereafter.

### Recordings

#### Circuit Mapping Using Laser Scanning Photostimulation (LSPS)

Local circuit maps of CSMN using LSPS were performed as described (Anderson et al., 2010). Briefly, slices were transferred to the recording chamber of an upright microscope (BX51, Olympus), and held in place with short pieces of flattened gold wire (0.813 mm diameter; Alfa Aesar). Fluorescently labeled CSMN with red microspheres were visualized in using epifluorescence optics and they were located in layer 5 (L5) of the motor cortex. Pipettes were fabricated from borosilicate capillaries with filaments (G150-F, Warner) using a horizontal puller (P-97, Sutter). A cesium-based intracellular solution was used for mapping excitatory and inhibitory inputs [composition, in mM: 128 mM CsMeSO_3_, 10 HEPES, 1 EGTA, 4 MgCl_2_, 4 ATP, and 0.4 GTP, 10 phosphocreatine, 3 ascorbate, and 0.05 Alexa-594 or 488 (Molecular Probes); pH 7.3]. The bath solution for photostimulation studies contained elevated concentrations of divalent cations (4 mM Ca^2+^ and 4 mM Mg^2+^) and an NMDA receptor antagonist (5 μM CPP; Tocris), to dampen neuronal excitability. Gabazine and NBQX were not included in the bath solution for glu-LSPS mapping studies. Caged glutamate (0.2 mM) was added directly to the bath solution. Voltages were not corrected for liquid junction potential. Recordings were performed at 21°C and were monitored for series resistance (inclusion criterion: <35 MΩ; mean: 17.3 MΩ). Once a patch recording of a labeled neuron was established, an image of the slice (4× objective) was acquired before mapping for precise registration of the mapping grid. The mapping grid (16 × 16; 100 μm spacing) was rotated with the top row of the grid flush with the pia and the soma was centered horizontally in the grid. The grid locations were sampled (every 0.4 s) with a UV stimulus 1.0 msec in duration and 20 mW at the specimen plane. Excitation profiles (EPs) were mapped as described (Weiler et al., 2008; Wood and Shepherd, 2010).

#### Intrinsic Properties Recordings

Electrophysiology recordings were performed as previously described (Martina et al., 2007). Briefly, slices were transferred to the recording chamber of an upright microscope (BX51, Olympus), and held in place with short pieces of flattened gold wire (0.813 mm diameter; Alfa Aesar). Fluorescently labeled CSMN neurons (GFP+ and red microsphere positive) were visualized using epifluorescence optics. Pipettes were fabricated from borosilicate capillaries with filaments (G150-F, Warner) using a horizontal puller (P-97, Sutter). The extracellular solution for these recordings was: (in mM: 125 NaCl, 1.25 KCl, 1.25 KH_2_PO_4_, 25 NaHCO_3_, and 16 glucose), and recording of intrinsic properties was performed in presence of blockers of fast synaptic transmission 10 μM DNQX, 50 μM APV, and 50 μM picrotoxin). For analysis of intrinsic excitability, the input/output curves obtained from each neuron were fit individually and we then calculated the mean and SEM of the fit parameters (reported in the text). I/V curves were investigated using 1 s long depolarizing current injections, with 100 pA steps. AP properties were investigated with measuring the properties of the first AP generated using 10 ms current injections, in 10 pA steps.

### Tissue Collection, Processing, and Immunocytochemistry

Mice were deeply anesthetized and perfused as previously described (Jara et al., 2012) The brain was dissected, post-fixed in 4% PFA overnight, stored in PBS with 0.01% sodium azide, and sectioned at 50 μm using Leica vibratome (Leica VT1000S, Leica Inc., Nussloch, Germany). Floating sections were processed for immunocytochemistry (Jara et al., 2017). In this study anti-GFP (1:500, Abcam, Cambridge, MA, USA), anti-GABARAPL1 (1:200, Proteintech, USA), anti-KCNV1 (1:200), anti-KCTD12 (1:200, Proteintech, USA), and anti-SCN3B (1:200, LSBio, USA) antibodies were used. All proper secondary antibodies were purchased from Abcam and used in 1:500 dilution. Immunocytochemistry was performed as previously reported (Jara et al., 2017). Expression of KCNV1, GABARAPL, KCTD12, and SCN3B was evaluated from three comparable sections of WT-UeGFP and hSOD1^G93A^-UeGFP mice (*n* = 3) spanning the motor cortex at P30 (Jara et al., 2017). Immunoreaction with KCTD12 could not be optimized and thus results could not be included in the text.

### Imaging and Data Collection

Nikon Eclipse TE2000-E (Nikon Inc., Melville, NY, USA), Leica TCS SP5 confocal microscope (Leica Inc., Bensheim, Germany), and Zeiss 880 confocal microscope (Carl Zeiss microscopy, Jena, Germany) were used to acquire low- and high-magnification images, respectively. Confocal microscopy imaging was performed at the Center for Advanced Microscopy/Nikon Imaging Center (CAM), at the Northwestern University Feinberg School of Medicine, Chicago. Plan Apo 40× Oil DIC H objective was used for image acquiring. All images were blindly taken with the same exposure and settings. The intensity of immunofluorescence was quantified using ImageJ software (NIH, USA). The soma of GFP+ CSMN in the motor cortex of both healthy and diseased mice were identified as regions of interest. The mean gray value depicting the level of immunofluorescent expression intensity for each protein investigated in this study were measured. At least 60 cells (from *n* = 3 mice) were examined for each protein of interest and each genotype.

### Statistical Analyses

Analysis of electrophysiology data was performed using Ephus software (Suter et al., 2010). Data analysis was performed offline using Matlab routines (Mathworks, Inc., Natick, MA, USA). Statistical comparisons between groups were made using Student-tests (for normally distributed data) or rank-sum test (for non-normally distributed data), as indicated. Error bars in plots represent SEM. In all cases, statistically significant differences were taken at *p* < 0.05.

## Results

### Intrinsic Subthreshold Characteristics and Photoexcitability of hSOD1^G93A^ CSMN Are Comparable to Healthy Controls

**Figure 1:**
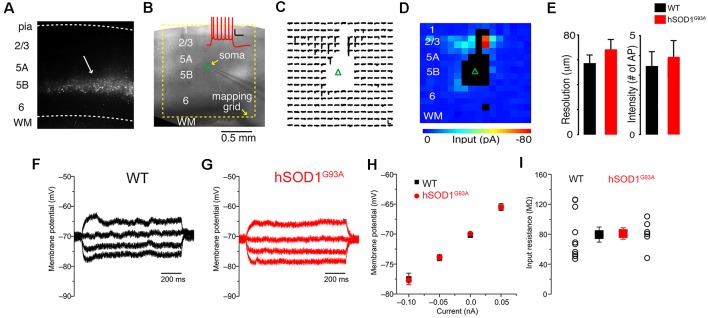
Excitation profiles (EPs) and intrinsic properties of corticospinal motor neurons (CSMN) in the motor cortex of wild type (WT) and hSOD1^G93A^ mice at P30. **(A)** Retrograde bead labeling of CSMN in the motor cortex. **(B)** Bright-field image of mapping configuration for CSMN (inset); Corticospinal action potentials generated from 100 pA current injection. **(C)** Trace map (16 × 16,100 μm spacing) showing EPSCs following stimulation of local presynaptic areas. The blank area represents areas that resulted in the direct stimulation of the recorded neuron. **(D)** Color map representing local excitatory inputs in **(C)**. **(E)** *Left*: resolution of photostimulation (the mean distance of spike evoking sites from the soma), *right*: intensity of photostimulation (total number of spikes per map per cell); WT (black) and hSOD1^G93A^ (red). The intrinsic properties of neurons from hSOD^G93A^ and WT mice were largely identical. The input resistance was measured from the slope of a linear fit to the voltage response to hyper- and depolarizing steps: Representative recordings of a **(F)** WT and **(G)** hSOD1^G93A^ CSMN. **(H)** Average V-I plot of WT (black) and hSOD1^G93A^ (red). **(I)** Summary plot showing that the input resistance is very similar between the groups (WT: *n* = 10; hSOD1^G93A^: *n* = 6).

CSMN were previously shown to display early signs of vulnerability in hSOD1^G93A^ mice (Ozdinler et al., 2011). To investigate the potential impact of synaptic and intrinsic factors contributing to CSMN vulnerability, we performed both LSPS mapping and patch-clamp recordings on retrogradely labeled CSMN in both WT and hSOD1^G93A^ mice (Figure 1A). Retrograde labeling surgeries are performed at P21 (post-natal day 21), and brain slices containing the motor cortex are prepared at P30, an early pre-symptomatic stage in the disease.

The local sources of excitatory and inhibitory synaptic inputs were mapped to these neurons using a photostimulation grid that was aligned to the pia of the motor cortex in coronal brain slice (Figure 1B). The 256 sites in the 16 × 16 square grids were visited at 1 Hz in a non-raster pattern that avoided the vicinity of recently stimulated sites, and excitatory responses were recorded in voltage-clamp mode at a holding potential of either −70 mV, close to the reversal potential for GABAergic responses, or 10 mV, close the reversal potential for glutamatergic responses, as previously reported (Weiler et al., 2008). Synaptic input maps were constructed by plotting the mean response amplitude in a 50 ms post-stimulus time window (Figure 1C). Responses contaminated by direct activation of the recorded neuron’s dendrites were excluded and rendered as black pixels in input color maps (Figure 1D). These maps thus represent “images” of the local sources of monosynaptic input, arising from small clusters of approximately 100 neurons at each stimulus location, to individual CSMN. Labeled CSMN in brain slices were targeted for patch-clamp recording and synaptic mapping so that their excitatory and inhibitory connectivity maps can be generated.

Differences in connectivity maps obtained from the motor cortex of hSOD1^G93A^ and WT mice could potentially be explained by differences in the photoexcitability of presynaptic neurons, rather than differences in synaptic connectivity. To investigate this possibility, we performed EPs—maps revealing the number and spatial distribution of photoexcitable sites across individual neurons—to quantitatively measure photoexcitability of presynaptic neurons directly as shown previously (Wood and Shepherd, 2010). In these calibration experiments, which were interleaved with synaptic input mapping from other neurons in the same slices, we recorded from L2/3 pyramidal neurons in loose seal mode for both WT and hSOD1^G93A^ mice while mapping their photoexitability using the same conditions as for synaptic input mapping (Figure 1D). We focused on L2/3 neurons as they are within the main presynaptic region of interest observed in synaptic input maps. The EP data sets were analyzed to determine: (1) the mean distance of spike evoking sites from the soma, an estimator of the resolution of photostimulation (Figure 1E, left); and (2) total number of spikes per map per cell, an estimator of the intensity of photostimulation (Figure 1E, right). These EP data suggested that photoexcitability between L2/3 neurons in hSOD1^G93A^ and WT littermates were comparable (Figure 1E).

Since intrinsic properties may also contribute to neuronal vulnerability, in a different set of experiments, we measured basic passive properties using a potassium-based intrapipette solution (Figures 1F–I). Resting membrane potential and input resistance were comparable between CSMN of WT and hSOD1^G93A^ mice (Figures 1F–I).

### CSMN of hSOD1^G93A^ Mice Receive Strong Inhibitory Input

**Figure 2:**
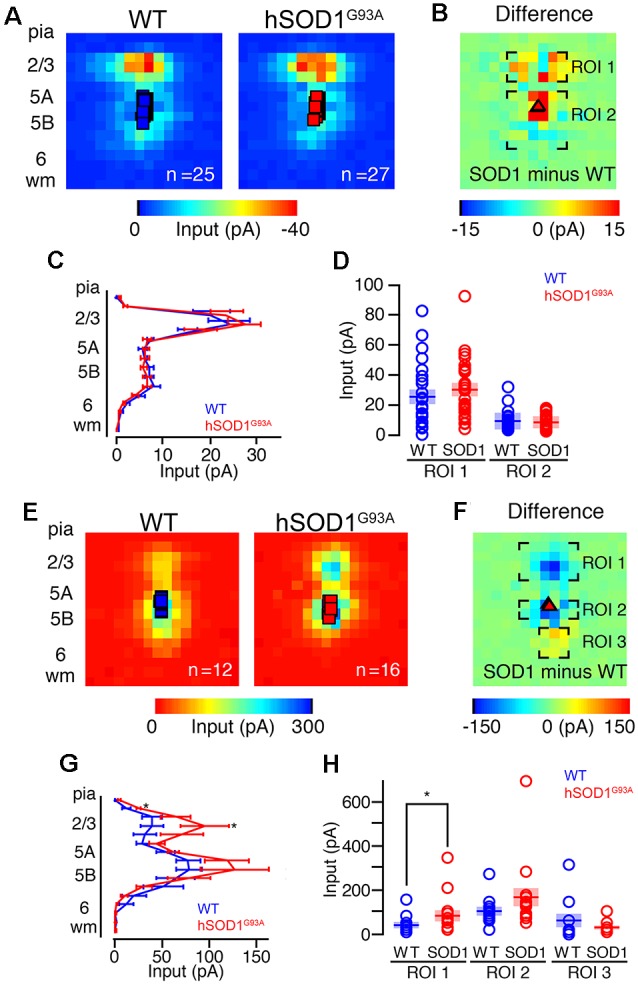
Excitation and inhibitory profiles of CSMN in the motor cortex of WT and hSOD1^G93A^ mice at P30. **(A)** Examples of EPs for WT (left) and hSOD1^G93A^ (right) neurons with excitatory color maps. Voids represent areas of direct excitation on the recorded neuron. **(B)** Mapping grid (16 × 16) depicting areas of analysis; Region-of-interest = ROI. **(C)** Row average of excitatory inputs within the analysis region. **(D)** Comparison of presynaptic excitatory input region-of-interest for WT and hSOD1^G93A^ CSMN. **(E)** Example inhibitory input trace maps for WT (left) and hSOD1^G93A^ (right) CSMN. **(F)** Mapping grid (16 × 16) depicting areas of analysis. **(G)** Row average of inhibitory inputs within the analysis region. **(H)** Comparison of inhibitory input region-of-interest for WT and hSOD1^G93A^ CSMN. *p < 0.05, Kruskal–Wallis test.

Our second set of experiments aimed to determine differences in local excitatory circuit strength of CSMN in hSOD1^G93A^ mice compared to WT littermates. Both WT (*n* = 25) and hSOD1^G93A^ CSMN (*n* = 27) in L5 received strong descending input from L2/3 (Figure 2A), as data obtained by averaging the maps and performing region-of-interest analyses revealed (Figure 2B). When the excitatory maps for each group were pooled, row analysis of inputs from a defined breadth of columns revealed similarities between two groups (Figure 2C). We next focused our analysis on a region of interest around the area of strongest L2/3 input (Figure 2D) and found no significant difference in excitatory circuit strength between CSMN of WT and hSOD1^G93A^ mice.

Unlike excitatory input maps, the inhibitory circuit mapping studies revealed a striking difference, especially at the site of L2/3 of the motor cortex. Using a cesium-based pipette (intracellular) solution and recording at a holding potential of ~0 mV, close to the reversal potential for glutamatergic inputs, we found inhibitory responses following stimulation of L2/3 and L5 for both WT and hSOD1^G93A^ CSMN (Figure 2E). We recorded inhibitory input maps from CSMN of hSOD1^G93A^ (*n* = 16) and WT (*n* = 12) mice, and performed a region-of-interest analysis (Figure 2F). When the inhibitory maps for each group were pooled, row analysis of inputs (Figure 2G) showed significantly larger inhibitory responses following stimulation of L2/3 and L5B. Although perisomatic L5B inhibitory responses appeared larger for CSMN of hSOD1^G93A^ mice, it was not significant. However, focused analysis around the region of strongest L2/3 input showed that inhibitory input resulting from L2/3 was significantly larger in CSMN of hSOD1^G93A^ mice (*p* < 0.05; Figure 2H).

### CPN Synaptic Input Confirms Cell-Type Specificity

**Figure 3:**
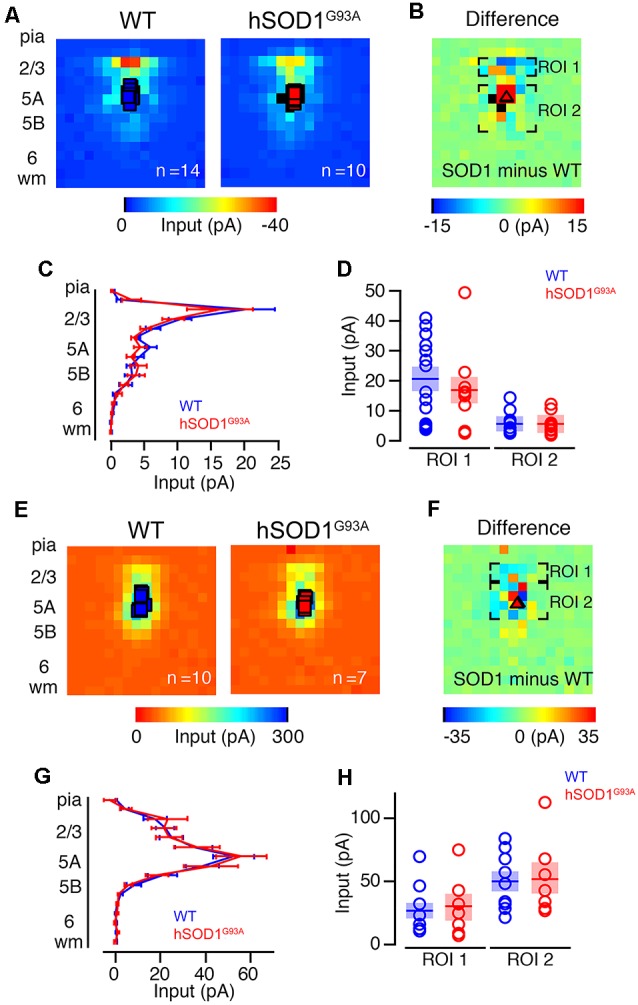
Excitation and inhibitory profiles of callosal projection neuron (CPN) in the motor cortex of WT and hSOD1^G93A^ mice at P30. Excitatory input maps for CPN of P30 WT and hSOD1^G93A^ mice. **(A)** Average excitatory color maps for WT (left) and hSOD1^G93A^ (right) CPN neurons. Voids represent areas of direct excitation on the recorded neuron. **(B)** Mapping grid (16 × 16) depicting areas of analysis; Region-of-interest = ROI. **(C)** Row average of excitatory inputs within the analysis region. **(D)** Comparison of presynaptic excitatory input region-of-interest for WT and hSOD1^G93A^ CPN neurons. Inhibitory input maps for CPN of WT and hSOD1^G93A^ mice at P30. **(E)** Average inhibitory color maps for WT (left) and hSOD1^G93A^ (right) CPN. **(F)** Mapping grid (16 × 16) depicting areas of analysis. **(G)** Row average of inhibitory inputs within the analysis region. **(H)** Comparison of inhibitory input region-of-interest for WT and hSOD1^G93A^ CPN.

To further investigate possible differences in photoexcitability, and to explore whether similar inhibitory and/or excitatory inputs are also observed in other projection neurons, we labeled CPNs at P30 by injecting fluorescent microspheres into the contralateral motor cortex at P21. We next mapped their excitatory and inhibitory inputs. CPN is located in lower layer 5A and receives strong input from layer 2/3 pyramidal neurons (Anderson et al., 2010), and therefore provides a relatively direct comparison to CSMN. Also, because CPN does not display early vulnerability in ALS, they serve as a good control to investigate whether observed effects are related to the disease state. CPN in WT and hSOD1^G93A^ mice in L5A showed strong excitatory inputs from L2/3 (Figures 3A–C). We recorded excitatory input maps from CPN of hSOD1^G93A^ (*n* = 10) and WT (*n* = 14) mice (Figure 3A), and mapping differences between WT and hSOD1^G93A^ (Figure 3B). These data showed no difference in L2/3 excitatory input for both row average (Figure 3C) and region-of-interest analysis (Figure 3D). As with CSMN, we found inhibitory responses following stimulation of L2/3 and L5 for CPN both in L5A of WT and hSOD1^G93A^ mice (Figure 3E). Analysis of inhibitory input maps recorded from CPN in hSOD1^G93A^ (*n* = 7) and WT (*n* = 10) mice (Figure 3F) also revealed comparable results between genotypes, further suggesting that the stronger inhibition observed in L2/3 of the motor cortex of hSOD1^G93A^ mice was specific to CSMN (Figures 3G,H).

### Inhibitory Transmission Is Altered in hSOD1^G93A^ CSMN

**Figure 4:**
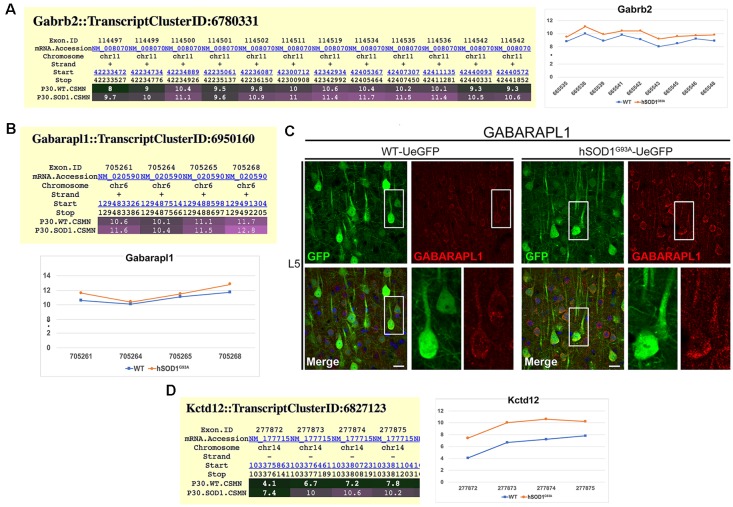
Molecular evidence of altered inhibitory transmission in hSOD1^G93A^ CSMN. **(A)** Gene expression analysis of Gabarb2. Expression values for each exon are plotted to the right. **(B)** Gene expression analysis data for Gabarapl1. Expression values for each exon is plotted at the bottom. **(C)** Representative images of GABARAPL1 expression in L5 motor cortex of WT-UeGFP (left) and hSOD1^G93A^-UeGFP (right). CSMN (green) and GABARAPL1 (red); insets are enlarged in the bottom right panels. Scale bar: 25 μm. **(D)** Gene expression analysis of Kctd12. Expression values for each exon are plotted to the right.

Since cortical connectivity studies suggested that CSMN receives increased inhibitory inputs especially at the site of L2/3, we next investigated whether the expression profile of inhibitory receptors was also altered in diseased CSMN. Exon microarray analysis performed using FACS-purified CSMN isolated from WT and hSOD1^G93A^ mice at P30, revealed that the expression profile of a distinct subset of GABA_A_ subunits were altered. For example, the expression of *Gabra4* and *Gabrb1* genes, coding for the α4 and β1 subunits (Supplementary Figure S1), and *Garb2* gene, which codes for the β2 subunits of the GABA receptor (Figure 4A) displayed increased expression profile in CSMN of hSOD1^G93A^ mice. In addition to the receptor subunits, auxiliary proteins also play a role in modulating the GABA receptor function. Therefore, we investigated potential changes in the expression profiles of some key modulators. Interestingly, the expression of GABA type A receptor-associated protein-like 1 (GABARAPL1), which plays a key role in its modulation (Chen et al., 2000), was also evident by immunofluorescence in WT CSMN (1745.65 ± 24.84 a.u. *n* = 88 cells) and in hSOD1^G93A^ CSMN (2148.1 ± 142.27 a.u. *n* = 113 cells; *p* = 0.098; *t-test*, Supplementary Figure S2, Figure 4B). Interestingly, the location of GABARAPL1 was primarily in discrete domains within the somatic membrane (Figure 4C). Also, the expression of Kctd12 (K channel tetramerization domain-containing protein 12), an important modulator of GABA towards desensitization, was present mainly in CSMN of hSOD1^G93A^ mice (Figure 4D).

### Intrinsic Electrical Properties of hSOD1^G93A^ CSMN and Their Molecular Background

**Figure 5:**
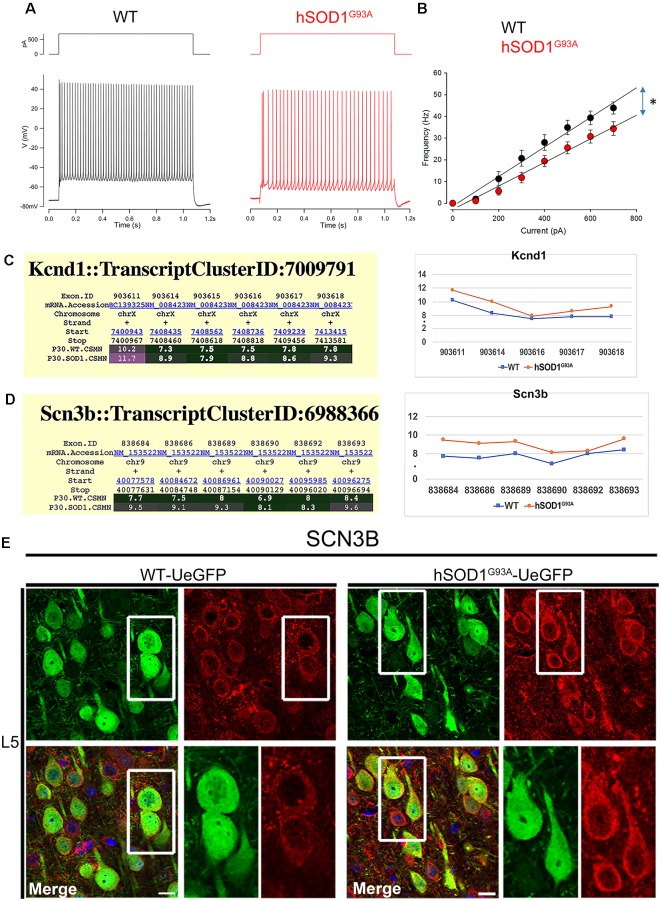
The excitability of CSMN in hSOD1^G93A^ mice. The excitability was measured as the number of action potential fired in response to 1 s depolarizing current steps of increasing amplitudes (Δ 0.05 nA). **(A)** Representative responses of WT and hSOD1^G93A^ CSMN to a 0.4 nA current step. **(B)** Average f-I curve of CSMN in WT and hSOD1^G93A^ mice. WT (black; *n* = 9) and hSOD1^G93A^ (red; *n* = 13). **(C)** Gene expression analysis of Kcnd1. Expression values for each exon are plotted to the right. **(D)** Gene expression analysis of Scn3b. Expression values for each exon are plotted to the right. **(E)** Representative images of Scn3b expression in L5 motor cortex of WT-UeGFP (left) and hSOD1^G93A^-UeGFP (right). CSMN (green) and Scn3b (red); insets are enlarged in the bottom right panels. Scale bar: 20 μm; *p ≤ 0.05.

The input resistance of hSOD1^G93A^ CSMN was not affected, as revealed by hyper- and hypo-polarization steps (Figures 1F,G). However, when injected with supra-threshold currents these neurons fired less because of a shallower current dependent increase in firing (the slope of a linear regression was 6.8 spikes/100 pA for WT CSMN (*n* = 9), and 5.4 spikes/100 pA for hSOD1^G93A^ CSMN (*n* = 13); *p* ≤ 0.05; Figure 5A). The maximum frequency with a 700 pA current injection was also lower in hSOD1^G93A^ CSMN (43.9 ± 2.8 Hz) vs. WT CSMN (34.4 ± 3.2 Hz, *p* ≤ 0.05; Figures 5A,B). This difference suggests the differential activity of a distinct voltage-gated Na^+^ and K^+^ channels. Since CSMN of hSOD1^G93A^ mice fired less, we investigated the potential changes in the expression profile of voltage-gated K^+^ and Na^+^ channel subunits. Among all subunits, Kcnh5, Kcnq2 (Supplementary Figure S1), and Kcnd1 (Figure 5C) expression was prominent in diseased CSMN at P30. Among Na^+^ subunits, we found that Scn2b (Supplementary Figure S1) and Scn3b (Figure 5D) were particularly increased in the CSMN of hSOD1^G93A^ mice at P30. Gene expression of Scn2b was also confirmed by immunocytochemical analysis (Figure 5E) and intensities of expression displayed an significant increase from WT CSMN (1,738.8 ± 33.9 a.u.; *n* = 73 cells) to hSOD1^G93A^ CSMN (2,463.5 ± 110.0 a.u.; *n* = 75 cells; *p* = 0.015; *t*-test, Supplementary Figure S2).

**Figure 6:**
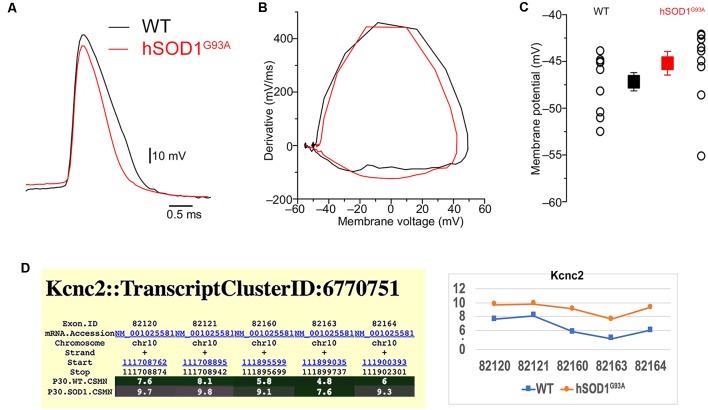
The form of action potential and the threshold in hSOD1^G93A^ CSMN.** (A)** Representative traces of action potentials in WT (black) and hSOD1^G93A^ (red) CSMN. The action potential threshold was measured from the first action potential fired in response to depolarizing current steps at the point where the first derivative was 10 mV/ms. **(B)** First derivatives of the traces in **(A)**. **(C)** Summary plot showing the action potential threshold for WT and hSOD1^G93A^ CSMN (WT: −47.2 ± 0.9 mV, *n* = 9; hSOD1^G93A^: −45.2 ± 1.3 mV, *n* = 13). **(D)** Gene expression analysis of Kcnc2. Expression values for each exon are plotted to the right.

**Figure 7:**
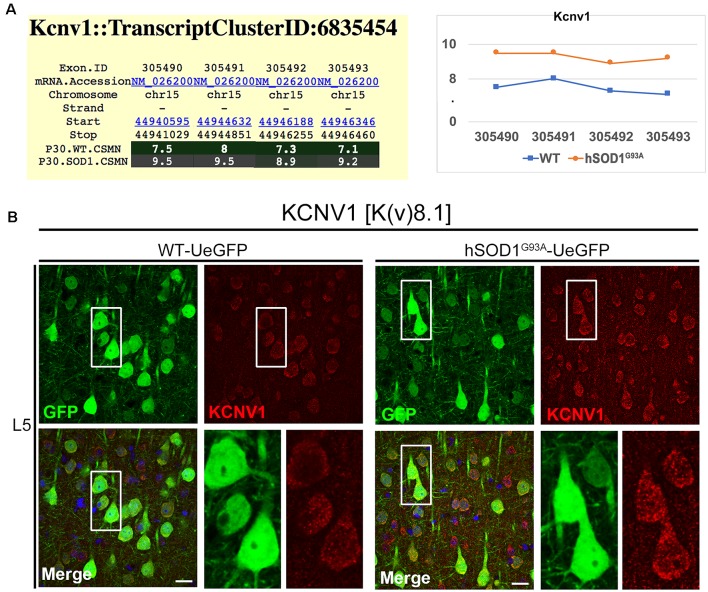
KCNV1 expression levels are increased in diseased CSMN. **(A)** Gene expression analysis of Kcnv1. Expression values for each exon are plotted to the right. **(B)** Representative images of Kcnv1 expression in L5 motor cortex of WT-UeGFP (left) and hSOD1^G93A^-UeGFP (right). CSMN (green) and Kcnv1 (red); insets are enlarged in the bottom right panels. Scale bar: 20 μm.

Interestingly, the properties of the first action potential generated by near-rheobase depolarizing current steps were comparable between WT and hSOD1^G93A^ CSMN (Figure 6A), including AP threshold (Figures 6B,C) and half duration [WT CSMN: 0.63 ± 0.017 ms (*n* = 9); hSOD1^G93A^ CSMN: 0.64 ± 0.04 ms (*n* = 13)], suggesting that the differences in spiking may depend on relatively slow K^+^ channels that are not activated during individual APs. Our data may suggest that differential expression of the A-type potassium channel subunit Kcnd1 (Figure 5C) and outward rectifying channel subunit Kcnh5 (Supplementary Figure S1) may contribute to the observed slower firing. Interestingly, expression of Kcnv1, which slows inactivation of Kv2 channels and is a negative modulator of Kv3 (Salinas et al., 1997) and thus may contribute to slower firing was present less in WT than in hSOD1^G93A^ CSMN, as revealed by exon microarray (Figure 7A, Supplementary Figure S2) and immunocytochemical analysis (WT CSMN: 2044.5 ± 132.5 a.u.; *n* = 61 cells; hSOD1^G93A^ CSMN: 2445.4 ± 146.8 a.u.; *n* = 67 cells; *p* = 0.22; *t*-test; Figure 7B). The presence of Kcnv1 may also help explain why having Kcnc2 (Kv3.2; Figure 6D) does not appear to affect AP duration.

## Discussion

Understanding the factors that contribute to UMN vulnerability and progressive degeneration has been an important quest for building effective treatments for numerous neurodegenerative diseases, in which voluntary movement is impaired. Since the cortex is complex and heterogeneous, we believe that the underlying causes are also complex and multi-factorial. The UMNs may have intrinsic problems related to their genes and/or proteins, but the environment they are in can also contribute to their disease state.

The UMNs are located in L5 of the motor cortex and they have a very long apical dendrite that extends towards the top layers of the brain. These neurons are unique in their ability to be connected by many different neuron types so that they can be properly modulated to convey cortical input towards spinal cord targets. Building evidence reveals spine loss is an early event in ALS (Jara et al., 2015). Also, extensive apical dendrite degeneration is observed in CSMN of many different mouse models of ALS (Ozdinler et al., 2011; Gautam et al., 2016, 2019; Genç et al., 2016) as well as Betz cells of a broad spectrum of ALS patients, including fALS, sALS and ALS/FTLD pathologies (Genç et al., 2017). Such architectural defects would impair proper modulation of UMNs, and would in part affect the motor neuron circuitry.

Previously, it was thought that the UMN loss is simply a byproduct of the ongoing spinal motor neuron degeneration. However, recent evidence revealed that cortical degeneration is an early event in ALS, so much so that cortical hyperexcitation occurs even before symptom onset in patients (Geevasinga et al., 2016). Hyperexcitation of neurons could be mediated by many different means. The neurons could be excited more by the excitatory neurons that converge unto them, the inhibitory neurons that inhibit them could be inhibited, or the neurons themselves may modulate expression profiles of key genes that code for the subunits of ion channels that help modulate their excitation and inhibition responses. Therefore, we expect that maintaining homeostasis is a dynamic state and perturbation of this state is the leading cause of neuronal vulnerability.

Studies in ALS patients (Caramia et al., 2000; Vucic et al., 2008; Van den Bos et al., 2018; Menon et al., 2019) demonstrate intracortical excitability, which can ultimately lead to spasticity and hyperreflexia, hallmarks of cortical pathology in ALS. ALS patients with C9orf72 expansions showed hyperexcitability as a feature of only symptomatic ALS patients (Geevasinga et al., 2015; Schanz et al., 2016), while other studies showed that cortical hyperexcitability appears early in the disease process in sALS patients (Eisen et al., 1993; Prout and Eisen, 1994; Mills and Nithi, 1997; Ziemann et al., 1997; Vucic and Kiernan, 2006), and precede the onset of the disease in fALS patients with SOD1 mutation (Vucic et al., 2008).

To understand the cortical abnormalities found in patients, several studies investigated the role of cortical neurons in the hSOD1^G93A^ ALS mouse model (Gurney et al., 1994). Different studies in motor areas of the neocortex found evidence for an increase in inhibitory circuits (Minciacchi et al., 2009) but also an increase in glutamate in the region of the motor cortex of symptomatic ALS hSOD1^G93A^ mice at P80 (Choi et al., 2009). A more recent study demonstrated early changes in pyramidal neurons in the motor cortex of the hSOD1^G93A^ mice before symptoms arise at P21 (Fogarty et al., 2015). Pyramidal neurons showed apical dendritic regression and intrinsic electrophysiological properties such as an increase in EPSC. Although this study does not directly implicate CSMN, it supports previous reports showing CSMN apical dendrite defects and spine loss. Our previous studies suggest that apical vacuolation and loss of spines in the hSOD1^G93A^ mice might play a key role in the local and distal microcircuits and that it might lead to CSMN dysfunction and overactivity by lack of proper neuronal modulation (Jara et al., 2014).

To reveal the underlying causes of neuronal vulnerability, we focused our attention on P30, a critical time point in CSMN vulnerability in the hSOD1^G93A^ mice. At this age, CSMN numbers are not yet significantly reduced and the mice show no behavioral defects. Interestingly, a thorough analysis of CSMN connectivity and electrophysiological properties at different stages of development and disease also suggested this age to be a critical time of vulnerability, as the neurons manage to maintain homeostasis briefly at this stage (Kim et al., 2017). Therefore, understanding the events that occur within and outside CSMN at this particular age is of biomedical significance. We thus analyzed CSMN functional connectivity in cortical circuits using glutamate uncaging and LSPS (Anderson et al., 2010), and intrinsic properties of CSMN (Martina et al., 2007).

We performed the first application of LSPS and cortical circuit mapping to CSMN in hSOD1^G93A^ mice. Also, we investigated the changes in the gene expression of key components of ion channel subunits, to examine the potential intrinsic changes that occur in diseased CSMN. We find subtle and yet important differences, some reaching significance, some not, during this early stage. For example, there is an enhancement of local inhibitory input especially following excitation of L2/3 for CSMN, but the same phenomenon is not observed in CPN, suggesting that the observed defects are cell-type specific. Likewise, CSMN appears to respond to inhibitory input, but it also activates expression of some key genes that are important for modulating voltage-gated Na^+^ and K^+^ currents, and thus an excitable state.

Neurons can receive inhibitory and excitatory inputs at the same time and from thousands of different neurons simultaneously. This is an immense undertaking. Especially UMNs, which are extensively modulated by both long-distance excitatory neurons and local circuitry neurons that are both excitatory and inhibitory, the balance between excitation and inhibition is very challenging to maintain. However, healthy neurons achieve this task and they become vulnerable only when they fail to maintain homeostasis.

For this study, inhibitory inputs were recorded using glutamate uncaging while holding a patch-clamped neuron at the reversal potential for glutamatergic responses. Inhibitory neurons are also a complex group of neurons with many different subtypes and specific functions (Kawaguchi and Kubota, 1997; Kawaguchi and Kondo, 2002; Apicella et al., 2012). They are present throughout the motor column and numerous studies tried to reveal potential changes in their numbers concerning disease progression. Even though some results appear to contradict, overall analyses suggest that the distribution and the function of interneurons are an important component of UMN circuitry (Ziemann et al., 1997; Clark et al., 2018). The inhibitory input can be monosynaptic (direct) and disynaptic (*via* another neuron). Therefore, understanding the imminent impact of the inhibitory input is challenging. Recorded inputs thus represent both monosynaptic inhibitory inputs resulting from stimulation of interneurons and disynaptic inhibitory inputs resulting from stimulation of pyramidal neurons, which subsequently excite interneurons. Our analysis shows that inhibitory inputs following L2/3 stimulation were significantly different. In a recent study, the main L2/3 → L5 inhibitory pathway for CSMN was shown to result from descending disynaptic excitation of low-threshold spiking interneurons in L5 (Apicella et al., 2012). Since the inhibitory input was mainly increased in L2/3, and not L5, we speculate that the difference in inhibitory inputs following L2/3 stimulation is due to a strengthening of the L2/3 → L5 inhibitory pathway, and not due to changes in enhanced L5 inhibitory neuron excitation. This in part may suggest that, since inhibitory neurons located in L2/3, whose goal is to inhibit the inhibitory neurons in L5, are more activated, they may thus inhibit the inhibitory neurons in L5 more. Dual clamp experiments are required to investigate the accuracy of this phenomenon.

The electrical properties of CSMN at this early stage of P30 demonstrated conflicting excitatory and inhibitory factors. Similar to our findings, there are reports to support hyperexcitability (van Zundert et al., 2008; Vucic et al., 2009; Wainger et al., 2014; Fogarty, 2018), but on the other hand also on reduced excitability (Mills, 2003; Delestrée et al., 2014; Leroy et al., 2014; King et al., 2016; Clark et al., 2018). We believe that hyper and/or hypo excitability is a dynamic phenomenon, and it is a function of disease state.

Interestingly, hyperexcitability has been reported as early as P4 and P5 (van Zundert et al., 2008; Kim et al., 2017), and in the course of development and disease progression neurons adapt their functional properties to normalize cortical excitability at P26–40 (Kim et al., 2017). However, as the disease progresses without intervention at P90–129 hyperexcitability appears again (Kim et al., 2017). The question arises if the cause of this modulation of excitability is extrinsic or intrinsic. Although based on the LSPS experiments and cortical circuit mapping, the extrinsic local inhibitory circuitry could not be excluded, several intrinsic factors were pointing to the specificity of CSMN electrical changes in the ALS mouse model. Foremost, in line with the increase in the inhibitory maps in L2/3, the inhibitory synapses to L5 CSMNs are strengthened as indicated by overexpression of GABA_A_ receptor subunits genes *Gabra4* *Gabrb1*, and *Gabrb2* (α4, β1 and β2 subunits, respectively). Clustering of these receptors at the postsynaptic membrane may also contribute to synaptic strengthening, and in fact, it was found by gene analysis and immunocytochemistry that the GABARAPL1 was upregulated in CSMN. This protein is known to cause the clustering of GABA_A_ receptors at the cell membrane (Chen et al., 2000), but it can also promote autophagy, particularly in neurons (Le Grand et al., 2013). The latter could be a housekeeping mechanism by which to regulate overinhibition of CSMN, which also serves as a compensatory mechanism. On the other hand, we have observed an abundant overexpression of the Kctd12 gene for the modulatory protein that acts on the kinetics of activation and desensitization of GABA_B_ receptors and thus may counteract the rise of the inhibitory input by GABA_A_ receptors (Li et al., 2017). This is rather significant because Kctd12 is one of the most potent modulators of GABA inhibition and has the demonstrated potential to reverse its effect. Only diseased CSMN express very high levels of Kctd12 and it could represent its effort to counteract the GABA inhibition.

On the other hand, the intrinsic basis of the perturbed balance of excitatory and inhibitory factors was evidenced by further gene expression analysis demonstrating overexpression of potassium voltage-gated channels Kcnc2, as well as Kcnd1 and Kcnh5, known to decrease neuronal firing (Bean, 2007; Martina et al., 2007; Brown and Passmore, 2009; Buskila et al., 2019). We also found that upon current injection, hSOD1^G93A^ CSMN fire at a slower pace compared to CSMN of WT littermates. Gene expression analysis revealed a potential explanation for the molecular background of such behavior in an overexpression of sodium channel beta subunits, particularly beta 2 and 3 (Scn2b and Scn3b), which were also found to be overexpressed in hSOD1^G93A^ mouse spinal cord MNs, albeit at later stages (Nutini et al., 2011, also see van Zundert et al., 2008; King et al., 2016; Sirabella et al., 2018). On the other hand, intense punctate staining was revealed on CSMN somata for the Kcnv1 (Kv8.1) potassium channel known to act as a negative modulator of Kv2 and Kv3 channels (Salinas et al., 1997), thus suggesting also an excitatory profile.

Our studies begin to reveal the complex nature of events that occur at the motor cortex as well as the response mechanism CSMN develops to maintain homeostasis at this critical time of neuronal vulnerability. Even though CSMN does not yet show signs of neuronal degeneration at P30, it is in a very active state with enhanced gene expression of key GABA receptor subunits, modulatory proteins as well as subunits of voltage-gated ion channels. Also, the activation of inhibitory neurons, especially in L2/3, deserves much more attention to gain a full understanding of the intrinsic and the extrinsic mechanisms responsible for UMN vulnerability. Here, we reveal the key players involved in CSMN excitation and inhibition at this critical hour of neuronal vulnerability. Our findings suggest key targets for direct modulation of UMN activity either towards inhibition or excitation states. This information is required for building effective and long-term treatment strategies by helping vulnerable neurons retain their homeostasis.

## Data Availability Statement

All datasets generated for this study are included in the Supplementary Material.

## Ethics Statement

The animal study was reviewed and approved by Northwestern University Animal Care and Use Committee.

## Author Contributions

PS, JJ, and PO designed the photostimulation electrophysiology experiments. PS conducted and analyzed the LSPS electrophysiology data. MN, JJ, MM, and PO designed the electrophysiology experiments to evaluate CSMN intrinsic properties. JJ performed all exon microarray experiments. MN conducted and analyzed the electrophysiology data. CB, DH, MP, PO, and JJ performed and analyzed all immunocytochemistry data. JJ performed all surgical procedures. JJ, PS, MM, PA, and PO wrote the manuscript.

## Conflict of Interest

The authors declare that the research was conducted in the absence of any commercial or financial relationships that could be construed as a potential conflict of interest.

## Supplementary Material

FIGURE S1 List of all genes investigated for their expression profile using exon microarray. CSMN isolated from WT and hSOD1^G93A^ mice at P30 is used for these gene expression analyses. The table displays the average of three independent experiments, and the results are log 2 transformed and color-coded to reveal the difference in levels of expression.

FIGURE S2 Bar graph representation for the average intensity of fluorescence for GABARAPL1, SCN3B, and KCNV1 expression in CSMN of WT (black box) and hSOD1^G93A^ (white box) mice at P30. Data are shown as mean ± SEM of three independent experimental replicates. *T*-test is used to determine statistical significance, and **p* < 0.05 is considered significant.
