# Supplementary material for: The Electrophysiological Determinants of Corticospinal Motor Neuron Vulnerability in ALS
Source: Front Mol Neurosci. 2020 May 19;13:73. doi: 10.3389/fnmol.2020.00073 (PMC7248374; doi:10.3389/fnmol.2020.00073)
Supplement: FIGURE S1 — List of all genes investigated for their expression profile using exon microarray. CSMN isolated from WT and hSOD1G93A mice at P30 is used for these gene expression analyses. The table displays the average of three independent experiments, and the results are log 2 transformed and color-coded to reveal the difference in levels of expression. [file Image_1.pdf]

Gabarap::TranscriptClusterID:6782090

Exon.ID	120860	120860	120862	120863	120864	120865	120865	120865
mRNA.Accession	NM_019749	NM_019749	NM_019749	NM_019749	NM_019749	NM_019749	NM_019749	NM_019749
Chromosome	chr11	chr11	chr11	chr11	chr11	chr11	chr11	chr11
Strand	+	+	+	+	+	+	+	+
Start	69805184	69805215	69805880	69806100	69807980	69808050	69808129	69808416
Stop	69805208	69805281	69805908	69806130	69808008	69808090	69808336	69808441
P30.WT.CSMN	9	8.8	8.9	10.4	6.5	10.7	8.5	5.6
P30.SOD1.CSMN	10.8	10.1	9.3	11.9	10.4	12	10.1	7.7

Gabarapl1::TranscriptClusterID:6950160

Exon.ID	705261	705261	705261	705261	705264	705265	705268	705268	705268	705268
mRNA.Accession	NM_020590	NM_020590	NM_020590	NM_020590	NM_020590	NM_020590	NM_020590	NM_020590	NM_020590	NM_020590
Chromosome	chr6	chr6	chr6	chr6	chr6	chr6	chr6	chr6	chr6	chr6
Strand	+	+	+	+	+	+	+	+	+	+
Start	129483188	129483326	129483396	129483465	129487514	129488598	129491120	129491304	129492225	129492280
Stop	129483319	129483386	129483458	129483507	129487566	129488697	129491175	129492205	129492278	129492319
P30.WT.CSMN	9	10.6	7.7	10	10.1	11.1	11.6	11.7	10.3	11.2
P30.SOD1.CSMN	9.5	11.6	9.4	10	10.4	11.5	12.1	12.8	11.4	12.2

Gabarapl2::TranscriptClusterID:6759622

Exon.ID	42051
mRNA.Accession	NM_026693
Chromosome	chr1
Strand	-
Start	71756554
Stop	71756595
P30.WT.CSMN	9.7
P30.SOD1.CSMN	11

Gabarapl2::TranscriptClusterID:6936712

Exon.ID	657813	657813	657813
mRNA.Accession	ENSMUST00000034428	NM_026693	NM_026693
Chromosome	chr5	chr5	chr5
Strand	-	-	-
Start	23623797	23624409	23624486
Stop	23623927	23624450	23624535
P30.WT.CSMN	11.2	9.8	6.8
P30.SOD1.CSMN	12.6	10.9	7.9

Gabarapl2::TranscriptClusterID:6939242

Exon.ID	666663	666664	666664
mRNA.Accession	ENSMUST00000034428	NM_026693	NM_026693
Chromosome	chr5	chr5	chr5
Strand	-	-	-
Start	76455988	76456320	76456434
Stop	76456083	76456395	76456473
P30.WT.CSMN	7.4	9.4	10.5
P30.SOD1.CSMN	8.1	10.7	11.9

Gabarapl2::TranscriptClusterID:6979181

Exon.ID	806678	806678	806678	806679	806680	806681	806682	806682	806684
mRNA.Accession	AK134873	NM_026693	NM_026693	NM_026693	NM_026693	ENSMUST00000034428	AK049819	AK134873	AK049819
Chromosome	chr8	chr8	chr8	chr8	chr8	chr8	chr8	chr8	chr8
Strand	+	+	+	+	+	+	+	+	+
Start	114464609	114464725	114465060	114466405	114476217	114476509	114476817	114477273	114478290
Stop	114464637	114464752	114465101	114466463	114476305	114476639	114477045	114477298	114479250
P30.WT.CSMN	7.5	8.7	9.7	9.5	9.8	11.2	5.3	2.3	4.8
P30.SOD1.CSMN	8.8	9.1	11.2	10.6	10.9	12.7	6.9	2.5	6.5

Gabbr1::TranscriptClusterID:6850271

Exon.ID	359119	359120	359120	359121	359123	359123	359123	359124	359128	359128	359129	359130	359131	359132
mRNA.Accession	NM_019439	AK134754	NM_019439	NM_019439	NM_019439	AK134754	AK134754	AK134754	ENSMUST00000113650	NM_019439	NM_019439	NM_019439	NM_019439	NM_019439
Chromosome	chr17	chr17	chr17	chr17	chr17	chr17	chr17	chr17	chr17	chr17	chr17	chr17	chr17	chr17
Strand	+	+	+	+	+	+	+	+	+	+	+	+	+	+
Start	37182947	37183531	37183683	37184399	37185367	37185585	37185681	37185943	37187816	37187959	37191076	37191704	37192808	37193251
Stop	37183091	37183628	37183716	37184596	37185518	37185667	37185710	37186366	37187923	37188089	37191187	37191811	37192861	37193297
P30.WT.CSMN	7.5	7.5	9.1	10.7	10	10.6	7.9	8.6	10.4	10.9	11.1	11.5	11	12.3
P30.SOD1.CSMN	8.8	9.1	10.3	11.7	11.2	12.1	9.2	9.6	11.7	12.2	12.2	12.3	12.1	12.9

Exon.ID	359133	359134	359135	359136	359138	359139	359140	359140	359141	359143	359144	359144	359144	359144	359145
mRNA.Accession	NM_019439	NM_019439	NM_019439	NM_019439	NM_019439	NM_019439	NM_019439	NM_019439	NM_019439	NM_019439	NM_019439	NM_019439	NM_019439	NM_019439	AK038844
Chromosome	chr17	chr17	chr17	chr17	chr17	chr17	chr17	chr17	chr17	chr17	chr17	chr17	chr17	chr17	chr17
Strand	+	+	+	+	+	+	+	+	+	+	+	+	+	+	+
Start	37193774	37199493	37200279	37201605	37204173	37204738	37206062	37206258	37206940	37207664	37208798	37209091	37209805	37210233	37211603
Stop	37193899	37199694	37200338	37201643	37204246	37204833	37206147	37206340	37206972	37207726	37208917	37209227	37209964	37210834	37212232
P30.WT.CSMN	12.2	12.3	11	12.1	9.6	10.2	10.7	10.1	6.3	10.2	11.3	10.7	11.3	11.6	7.4
P30.SOD1.CSMN	13.2	12.8	11.7	12.8	10.5	11.2	11.6	10.8	5.9	11.3	12.4	11.6	11.9	12.8	8.1

Gabbr2::TranscriptClusterID:6921387

Exon.ID	603024	603024	603024	603024	603024	603025	603026	603027	603028	603029
mRNA.Accession	NM_001081141	NM_001081141	NM_001081141	NM_001081141	NM_001081141	NM_001081141	NM_001081141	NM_001081141	NM_001081141	NM_001081141
Chromosome	chr4	chr4	chr4	chr4	chr4	chr4	chr4	chr4	chr4	chr4
Strand	-	-	-	-	-	-	-	-	-	-
Start	46677039	46677288	46677554	46677656	46677696	46680397	46687323	46690345	46694016	46697144
Stop	46677191	46677510	46677635	46677687	46677820	46680454	46687430	46690516	46694176	46697221
P30.WT.CSMN	8.8	11.2	8.5	8.7	9.5	10.4	11.1	11.1	11.5	11.3
P30.SOD1.CSMN	10.2	12.2	10	9.4	11	11.5	12.1	11.9	12.7	12.3

Exon.ID	603037	603040	603045	603047	603049	603055	603064	603068	603069	603073
mRNA.Accession	NM_001081141	NM_001081141	NM_001081141	NM_001081141	NM_001081141	NM_001081141	NM_001081141	NM_001081141	NM_001081141	NM_001081141
Chromosome	chr4	chr4	chr4	chr4	chr4	chr4	chr4	chr4	chr4	chr4
Strand	-	-	-	-	-	-	-	-	-	-
Start	46731717	46737173	46746930	46749264	46752666	46762394	46800310	46811385	46816931	46828510
Stop	46731775	46737258	46747023	46749330	46752734	46762426	46800485	46811529	46816992	46828586
P30.WT.CSMN	10.9	10.8	10.5	10.7	11.5	5.6	11.7	11.3	11.1	11.2
P30.SOD1.CSMN	12	12	11.6	12.1	12.5	5.7	12.5	12	11.9	11.8

Exon.ID	603085	603094	603103	603104	603105	603120	603121
mRNA.Accession	NM_001081141	NM_001081141	AK035872	AK035872	AK035872	NM_001081141	NM_001081141
Chromosome	chr4	chr4	chr4	chr4	chr4	chr4	chr4
Strand	-	-	-	-	-	-	-
Start	46859216	46888550	46918448	46919829	46921330	47003987	47004424
Stop	46859308	46888640	46919059	46919892	46921674	47004119	47004668
P30.WT.CSMN	11	10.7	5.4	5.2	6.2	10.2	6.6
P30.SOD1.CSMN	11.6	11.6	7	5.7	5.9	11	8.1

Gabra1::TranscriptClusterID:6787527

Exon.ID	141483	141483	141484	141484	141485	141486	141487	141489	141489	141491
mRNA.Accession	NM_010250	NM_010250	NM_010250	NM_010250	NM_010250	NM_010250	NM_010250	NM_010250	NM_010250	NM_010250
Chromosome	chr11	chr11	chr11	chr11	chr11	chr11	chr11	chr11	chr11	chr11
Strand	-	-	-	-	-	-	-	-	-	-
Start	41944517	41947010	41948910	41949003	41953775	41960613	41966837	41968327	41968496	41976056
Stop	41946953	41947202	41948983	41949102	41953893	41960721	41966915	41968459	41968521	41976121
P30.WT.CSMN	10.2	10.6	12.1	11.5	11.3	11.3	11.7	11.5	10.9	12.4
P30.SOD1.CSMN	11	11.8	12.5	12.5	11.8	11.7	12.2	12.1	11.2	12.8

141493	141494	141495	141496	141498	141499	141500	141502	141502	141502
NM_010250	AK142450	AK142450	NM_010250	AK020669	AK020669	AK020669	NM_010250	NM_010250	NM_010250
chr11	chr11	chr11	chr11	chr11	chr11	chr11	chr11	chr11	chr11
-	-	-	-	-	-	-	-	-	-
41989408	41990844	41992809	41993078	41994528	41994954	41995217	41995686	41995819	41996241
41989511	41991043	41992985	41993118	41994712	41995035	41995331	41995799	41995980	41996407
11	4.8	3.9	10.5	6.5	6.7	7.2	9.5	8.9	4
11.6	4.5	3.1	11.3	7.3	6.2	6.9	10.8	10.3	5.8

Gabra2::TranscriptClusterID:6939005

Exon.ID	665481	665481	665482	665482	665482	665483	665487	665488	665490	665490
mRNA.Accession	AK036049	AK048165	NM_008066	NM_008066	NM_008066	NM_008066	NM_008066	NM_008066	NM_008066	AK046953
Chromosome	chr5	chr5	chr5	chr5	chr5	chr5	chr5	chr5	chr5	chr5
Strand	-	-	-	-	-	-	-	-	-	-
Start	71350003	71352007	71352548	71353043	71353119	71364721	71397357	71399194	71403962	71404220
Stop	71351806	71352033	71352837	71353088	71353319	71364856	71397433	71399275	71404022	71404871
P30.WT.CSMN	10.2	11.4	9.9	8.5	9.2	8.5	9.6	8.7	9.2	4.9
P30.SOD1.CSMN	10.7	11.4	9.6	6.9	9.4	9	10.4	9	9.8	5.5

665491	665492	665494	665495	665496	665505	665507	665510
NM_008066	NM_008066	NM_008066	AK045091	AK045091	NM_008066	NM_008066	NM_008066
chr5	chr5	chr5	chr5	chr5	chr5	chr5	chr5
-	-	-	-	-	-	-	-
71405677	71405819	71425990	71434757	71436154	71483212	71485800	71486954
71405798	71405869	71426036	71435074	71436545	71483326	71485826	71487068
8.9	8	8.8	7.5	4.9	10.2	8.8	7.9
9.7	7.7	8.4	6.6	5	11.1	10	9

Gabra3::TranscriptClusterID:7017519

Exon.ID	927216
mRNA.Accession	NM_008067
Chromosome	chrX
Strand	-
Start	69678040
Stop	69678596
P30.WT.CSMN	6.6
P30.SOD1.CSMN	7.4

Gabra3::TranscriptClusterID:7017520

Exon.ID	927217	927217	927219	927220	927221	927223	927225	927226	927228	927228
mRNA.Accession	NM_008067	NM_008067	NM_008067	NM_008067	NM_008067	NM_008067	AK082413	AK082413	NM_008067	NM_008067
Chromosome	chrX	chrX	chrX	chrX	chrX	chrX	chrX	chrX	chrX	chrX
Strand	-	-	-	-	-	-	-	-	-	-
Start	69679308	69679989	69690554	69696850	69704936	69721565	69731644	69734052	69746429	69746521
Stop	69679734	69680273	69690671	69696987	69705070	69721642	69731878	69734356	69746501	69746620
P30.WT.CSMN	8.7	10.5	10.9	11.4	10.8	9.8	6.8	5.3	10	10.5
P30.SOD1.CSMN	9.4	11.5	11.5	12.1	11.6	10.7	5.4	4.9	10.5	11.6

927229	927232	927233	927234	927240	927241
NM_008067	NM_008067	NM_008067	NM_008067	NM_008067	ENSMUST00000114554
chrX	chrX	chrX	chrX	chrX	chrX
-	-	-	-	-	-
69756644	69785408	69798038	69798156	69901352	69901998
69756701	69785503	69798137	69798190	69901460	69902164
10.2	10.7	9.1	8.8	7.5	2.3
11.1	11.4	9.2	9.2	7.9	3.5

Gabra4::TranscriptClusterID:6939019

Exon.ID	665534	665535	665538	665539	665541	665542	665543	665545	665546
mRNA.Accession	NM_010251	NM_010251	NM_010251	NM_010251	NM_010251	NM_010251	NM_010251	NM_010251	AK079551
Chromosome	chr5	chr5	chr5	chr5	chr5	chr5	chr5	chr5	chr5
Strand	-	-	-	-	-	-	-	-	-
Start	71961649	71963157	72015189	72024789	72029379	72032082	72032351	72033500	72046725
Stop	71962549	71963345	72015313	72024906	72029488	72032148	72032476	72033544	72046804
P30.WT.CSMN	6.8	8.8	10	8.9	9.8	9.1	8	8.5	5.3
P30.SOD1.CSMN	8.2	9.5	11.1	9.9	10.4	10.4	9.2	9.6	5.4

665546	665546	665546	665548	665548	665548	665548	665548
AK079551	AK139970	NM_010251	NM_010251	NM_010251	NM_010251	NM_010251	NM_010251
chr5	chr5	chr5	chr5	chr5	chr5	chr5	chr5
-	-	-	-	-	-	-	-
72047700	72048375	72048414	72048997	72049050	72049222	72049318	72049357
72048054	72048401	72048504	72049041	72049101	72049307	72049344	72049468
6.6	7.8	9.2	8.9	8	6.5	4.7	4.8
6.8	7.5	9.7	9.8	8.3	8.6	4.7	7.6

Gabra5::TranscriptClusterID:6967593

Exon.ID	767249	767249	767251	767254	767256	767258	767263	767264	767265	767267	767267	767271
mRNA.Accession	NM_176942	NM_176942	NM_176942	NM_176942	NM_176942	NM_176942	NM_176942	NM_176942	NM_176942	NM_176942	NM_176942	NM_176942
Chromosome	chr7	chr7	chr7	chr7	chr7	chr7	chr7	chr7	chr7	chr7	chr7	chr7
Strand	-	-	-	-	-	-	-	-	-	-	-	-
Start	64663185	64664015	64669013	64673609	64677153	64708337	64744228	64744523	64746126	64763393	64763435	64765141
Stop	64663579	64664132	64669128	64673742	64677255	64708385	64744382	64744564	64746226	64763426	64763468	64765240
P30.WT.CSMN	9.1	9.9	10.8	9.9	9.7	10.2	10.4	10.2	10.6	10.7	10	7.3
P30.SOD1.CSMN	9.4	9.2	11.1	10	9.8	10.4	11	10.5	11.1	10.8	9.9	7.8

Gabra6::TranscriptClusterID:6787531

Exon.ID	141512	141513	141513	141520	141521	141521	141522	141523	141523
mRNA.Accession	NM_001099641	NM_001099641	NM_001099641	NM_001099641	NM_001099641	NM_001099641	NM_001099641	NM_001099641	NM_001099641
Chromosome	chr11	chr11	chr11	chr11	chr11	chr11	chr11	chr11	chr11
Strand	-	-	-	-	-	-	-	-	-
Start	42120016	42120791	42120913	42128470	42129949	42130499	42130904	42131082	42131251
Stop	42120390	42120867	42120992	42128696	42130068	42130577	42130963	42131214	42131278
P30.WT.CSMN	5.3	2.2	5.5	5.9	2.5	3.3	3.9	3.5	3.2
P30.SOD1.CSMN	4.1	2.9	6.3	6	3.2	3.5	5.1	3.1	4.3

141524	141525	141526	141526	141526	141526	141526
NM_001099641	NM_001099641	NM_001099641	NM_001099641	NM_001099641	NM_001099641	NM_001099641
chr11	chr11	chr11	chr11	chr11	chr11	chr11
-	-	-	-	-	-	-
42133173	42133889	42134233	42134271	42134318	42134345	42134390
42133201	42133987	42134260	42134301	42134344	42134379	42134558
3.3	3	4.3	7.6	1.8	3.9	3.5
7.1	3.9	6	8.3	3.6	2.8	3.2

Gabrb1::TranscriptClusterID:6931512

Exon.ID	638204	638204	638205	638206	638214	638215	638216	638220	638220	638220	638221	638234
mRNA.Accession	BC157920	NM_008069	NM_008069	NM_008069	AK082309	AK082309	AK082309	AK036831	AK036831	AK036831	AK036831	NM_008069
Chromosome	chr5	chr5	chr5	chr5	chr5	chr5	chr5	chr5	chr5	chr5	chr5	chr5
Strand	+	+	+	+	+	+	+	+	+	+	+	+
Start	72091192	72091255	72091578	72092063	72139598	72140838	72142723	72158488	72158612	72158766	72160287	72260704
Stop	72091237	72091321	72091615	72092110	72140006	72141182	72142886	72158518	72158705	72159044	72160668	72260820
P30.WT.CSMN	6.1	9.2	8.7	9.9	7.1	7.8	7	5.5	6	4	6.4	8.7
P30.SOD1.CSMN	6.7	9.8	9.2	10.5	6.9	7.3	5.7	6.8	6.9	4.1	6.6	9.2

Exon.ID	638241	638242	638258	638260	638261	638261	638261	638261	638261
mRNA.Accession	AK138151	AK138151	NM_008069	ENSMUST00000118662	ENSMUST00000118662	NM_016980	XR_033972	NM_016980	XR_033972
Chromosome	chr5	chr5	chr5	chr5	chr5	chr5	chr5	chr5	chr5
Strand	+	+	+	+	+	+	+	+	+
Start	72286593	72287620	72421077	72438446	72438549	72438591	72438813	72438845	72438876
Stop	72287016	72287665	72421117	72438480	72438581	72438777	72438839	72438875	72438904
P30.WT.CSMN	7.3	7.2	6.2	5	5.4	3.8	4	8.2	7.1
P30.SOD1.CSMN	6.2	7	5.9	5.7	7.4	4.5	5.9	9.9	8

Exon.ID	638262	638263	638272	638275	638276	638277	638284	638284
mRNA.Accession	XR_033733	XR_033972	AK047796	NM_008069	NM_008069	NM_008069	NM_008069	NM_008069
Chromosome	chr5	chr5	chr5	chr5	chr5	chr5	chr5	chr5
Strand	+	+	+	+	+	+	+	+
Start	72439113	72439228	72492800	72499547	72499923	72513218	72527792	72528350
Stop	72439140	72439256	72493156	72499656	72500059	72513330	72527991	72530086
P30.WT.CSMN	7.6	4.3	5.4	5.9	9.1	9.1	9.5	9
P30.SOD1.CSMN	7.3	3.5	4.9	5.4	10.1	10.3	10.4	10.3

Gabrb1::TranscriptClusterID:6931514

Exon.ID	638287
mRNA.Accession	AK045528
Chromosome	chr5
Strand	+
Start	72531497
Stop	72531888
P30.WT.CSMN	8.5
P30.SOD1.CSMN	9.8

Gabrb2::TranscriptClusterID:6780331

Exon.ID	114497	114497	114499	114499	114500	114501	114502	114511	114519	114519	114530	114534	114535	114536	114536	114542	114542
mRNA.Accession	NM_008070	NM_008070	NM_008070	NM_008070	NM_008070	NM_008070	NM_008070	NM_008070	NM_008070	AK034069	AK141441	NM_008070	NM_008070	NM_008070	NM_008070	NM_008070	NM_008070
Chromosome	chr11	chr11	chr11	chr11	chr11	chr11	chr11	chr11	chr11	chr11	chr11	chr11	chr11	chr11	chr11	chr11	chr11
Strand	+	+	+	+	+	+	+	+	+	+	+	+	+	+	+	+	+
Start	42233259	42233472	42234734	42234783	42234889	42235061	42236087	42300712	42342934	42343900	42380298	42405367	42407307	42411048	42411135	42440093	42440572
Stop	42233415	42233527	42234776	42234817	42234926	42235137	42236150	42300908	42342992	42344960	42380731	42405464	42407450	42411119	42411281	42440331	42441852
P30.WT.CSMN	8.7	8	9	9.8	10.4	9.5	9.8	10	10.6	7.8	4.6	10.4	10.2	10.3	10.1	9.3	9.3
P30.SOD1.CSMN	9.7	9.7	10	10.5	11.1	9.6	10.9	11	11.4	8	5	11.7	11.5	11.2	11.4	10.5	10.6

Gabrb2::TranscriptClusterID:6780332

Exon.ID	114543	114543
mRNA.Accession	NM_008070	NM_008070
Chromosome	chr11	chr11
Strand	+	+
Start	42442850	42445761
Stop	42443756	42446045
P30.WT.CSMN	10.1	9.7
P30.SOD1.CSMN	11.2	11

Gabrb3::TranscriptClusterID:6960885

Exon.ID	741710	741711	741711	741711	741712	741713	741742	741749	741752	741753	741754	741756	741763	741765	741766
mRNA.Accession	NM_001038701	NM_008071	NM_008071	NM_008071	NM_008071	NM_008071	AK048929	NM_008071	AK051340	AK051340	NM_008071	NM_008071	NM_008071	NM_008071	NM_008071
Chromosome	chr7	chr7	chr7	chr7	chr7	chr7	chr7	chr7	chr7	chr7	chr7	chr7	chr7	chr7	chr7
Strand	+	+	+	+	+	+	+	+	+	+	+	+	+	+	+
Start	64845893	64846569	64846646	64846700	64846876	64847145	64977855	65020837	65030836	65032568	65044236	65047864	65067424	65071682	65081751
Stop	64846002	64846611	64846691	64846725	64846941	64847202	64978659	65020995	65031120	65032978	65044272	65047974	65067556	65071878	65083996
P30.WT.CSMN	6.3	12.1	10.3	10.6	10.3	11.1	7.9	10.5	7.7	6.8	12	11.9	12.1	11.5	11.2
P30.SOD1.CSMN	7.7	12.8	10.2	11.5	10.1	11.2	7.2	10.9	7.1	5.6	12.2	12.5	12.3	10.9	12

Gabrd::TranscriptClusterID:6927253

Exon.ID	624063	624063	624064	624064	624065	624066	624067	624068	624069	624072	624072
mRNA.Accession	NM_008072	NM_008072	NM_008072	NM_008072	NM_008072	NM_008072	NM_008072	NM_008072	NM_008072	NM_008072	NM_008072
Chromosome	chr4	chr4	chr4	chr4	chr4	chr4	chr4	chr4	chr4	chr4	chr4
Strand	-	-	-	-	-	-	-	-	-	-	-
Start	154759292	154759518	154760089	154760513	154761291	154761969	154762375	154762683	154763043	154772004	154772101
Stop	154759487	154759795	154760245	154760625	154761388	154762023	154762504	154762738	154763143	154772037	154772189
P30.WT.CSMN	4.5	6	6.6	7.9	6.6	6.9	7.7	8.2	7.2	9.9	6.5
P30.SOD1.CSMN	5.8	8.1	9.3	9.3	8.1	9.1	8.9	9.3	7.4	10.8	8.1

Gabre::TranscriptClusterID:7017513

Exon.ID	927185	927185	927185	927185	927186	927187	927187	927189	927191	927192	927193	927193
mRNA.Accession	NM_017369	NM_017369	NM_017369	NM_017369	NM_017369	NM_017369	NM_017369	mmu-mir-224	NM_017369	NM_017369	NM_017369	NM_017369
Chromosome	chrX	chrX	chrX	chrX	chrX	chrX	chrX	chrX	chrX	chrX	chrX	chrX
Strand	-	-	-	-	-	-	-	-	-	-	-	-
Start	69502798	69502857	69502995	69503082	69503526	69503799	69503866	69506372	69507804	69509102	69509858	69509976
Stop	69502856	69502964	69503034	69503171	69503608	69503845	69503905	69506441	69507914	69509164	69509945	69510042
P30.WT.CSMN	3.1	2.4	2.3	3.4	4.1	5.5	1.9	3	1.4	2.5	3.4	3.2
P30.SOD1.CSMN	5.1	4.4	4	4.1	5.8	4.3	2.5	5.7	2.8	4.8	4.6	3.9

Exon.ID	927194	927194	927195	927196	927196	927201	927202
mRNA.Accession	AK079258	NM_017369	NM_017369	NM_017369	NM_017369	NM_017369	AK079258
Chromosome	chrX	chrX	chrX	chrX	chrX	chrX	chrX
Strand	-	-	-	-	-	-	-
Start	69514362	69514568	69515028	69516171	69516431	69519985	69520084
Stop	69514538	69514612	69515138	69516392	69516474	69520040	69520227
P30.WT.CSMN	3.3	5.2	3	4.6	3.2	5	5.6
P30.SOD1.CSMN	5.8	6.8	5.8	7.3	3.5	5.1	6.9

Gabrg1::TranscriptClusterID:6938999

Exon.ID	665457	665458	665460	665464	665464	665465	665466	665467	665468	665468	665468
mRNA.Accession	NM_010252	NM_010252	NR_003967	NM_010252	NM_010252	NM_010252	NM_010252	NM_010252	NM_010252	NM_010252	NM_010252
Chromosome	chr5	chr5	chr5	chr5	chr5	chr5	chr5	chr5	chr5	chr5	chr5
Strand	-	-	-	-	-	-	-	-	-	-	-
Start	71142515	71145348	71157322	71165553	71165633	71168358	71169321	71171907	71173485	71173613	71173672
Stop	71143851	71145521	71157354	71165598	71165719	71168478	71169397	71171960	71173565	71173639	71173705
P30.WT.CSMN	5	3.5	9.9	4.2	4.8	5.8	5	5.3	6.5	2.6	2.5
P30.SOD1.CSMN	5	6.3	9.7	5.2	6.1	9	7.3	7.4	8.4	5.1	3.8

Exon.ID	665472	665473	665473	665473	665475	665475
mRNA.Accession	NM_010252	AK038686	AK038686	NM_010252	NM_010252	NM_010252
Chromosome	chr5	chr5	chr5	chr5	chr5	chr5
Strand	-	-	-	-	-	-
Start	71185913	71205082	71207098	71207196	71233356	71233555
Stop	71185952	71206808	71207191	71207292	71233420	71233644
P30.WT.CSMN	4.7	2.3	2.6	6.1	5.8	6.7
P30.SOD1.CSMN	7.1	3.7	1.9	6.7	7	8.3

Gabrg2::TranscriptClusterID:6787525

Exon.ID	141449	141449	141449	141449	141450	141451	141451	141451	141452	141452	141452
mRNA.Accession	NM_177408	NM_008073	NM_008073	AK078261	AK078261	ENSMUST00000109290	ENSMUST00000109290	NM_008073	AK034338	NM_008073	NM_008073
Chromosome	chr11	chr11	chr11	chr11	chr11	chr11	chr11	chr11	chr11	chr11	chr11
Strand	-	-	-	-	-	-	-	-	-	-	-
Start	41724729	41725385	41725819	41726117	41726934	41728538	41729756	41729808	41732892	41733916	41734009
Stop	41725048	41725671	41726077	41726227	41727440	41729724	41729785	41729980	41733890	41733998	41734038
P30.WT.CSMN	8.2	9.5	9.7	5.1	5.4	7.1	1.1	11.3	7.6	11	11.2
P30.SOD1.CSMN	8.8	10.6	10.9	3.8	5.6	6.9	2.2	12.3	6.2	12.3	12.6

Exon.ID	141461	141462	141463	141464	141465	141466	141467	141468	141469	141470	141471
mRNA.Accession	NM_008073	NM_008073	NM_008073	NM_008073	NM_008073	AK043062	AK043062	AK048859	AK048859	AK048859	AK048859
Chromosome	chr11	chr11	chr11	chr11	chr11	chr11	chr11	chr11	chr11	chr11	chr11
Strand	-	-	-	-	-	-	-	-	-	-	-
Start	41781059	41782316	41785215	41788329	41790055	41790904	41792348	41797174	41797822	41798251	41798665
Stop	41781172	41782369	41785402	41788375	41790187	41790934	41792755	41797484	41797875	41798641	41798716
P30.WT.CSMN	10.2	10.4	10.6	9	10.1	3.1	7.5	4.2	2.6	6.5	3.8
P30.SOD1.CSMN	11.1	11.6	11.4	10	10.1	2.8	6.6	5.2	3.6	6.4	2.4

Exon.ID	141472	141473	141474	141475	141475	141475	141475
mRNA.Accession	AK133692	AK160669	NM_008073	NM_008073	NM_008073	NM_008073	AK034338
Chromosome	chr11	chr11	chr11	chr11	chr11	chr11	chr11
Strand	-	-	-	-	-	-	-
Start	41800129	41802125	41813729	41813794	41813857	41814067	41814160
Stop	41800157	41802668	41813769	41813822	41814030	41814127	41814337
P30.WT.CSMN	2.9	7	9.8	6.6	7.9	5.4	4
P30.SOD1.CSMN	2.2	6.7	10.8	7.5	9.4	6	4

Gabrg3::TranscriptClusterID:6967584

Exon.ID	767170	767170	767172	767173	767174	767180	767193	767194	767204	767204	767208	767209	767213	767214	767224	767224
mRNA.Accession	NM_008074	NM_008074	NM_008074	NM_008074	NM_008074	NM_008074	NM_008074	NM_008074	BC138962	BC138962	AK034328	AK034328	AK138341	AK138341	AK046483	NM_008074
Chromosome	chr7	chr7	chr7	chr7	chr7	chr7	chr7	chr7	chr7	chr7	chr7	chr7	chr7	chr7	chr7	chr7
Strand	-	-	-	-	-	-	-	-	-	-	-	-	-	-	-	-
Start	63979650	63979793	63984651	63984979	63990334	64028631	64238027	64240380	64433828	64434978	64472320	64475459	64497341	64497800	64578445	64579018
Stop	63979748	63980030	63984703	63985147	63990438	64028723	64238077	64240511	64434450	64435089	64472515	64475893	64497554	64498176	64578989	64579080
P30.WT.CSMN	10	10.6	9.1	10.1	9.1	9	8.2	9.8	7.9	8.4	7.4	6.4	6.9	7.1	7.8	8.7
P30.SOD1.CSMN	9.6	10.4	9	10.4	8.9	9.5	7.9	10.4	8.2	7.4	6.1	5.7	7	6.8	7.9	9

Exon.ID	767232	767234	767234	767234	767234
mRNA.Accession	NM_008074	NM_008074	NM_008074	ENSMUST00000068394	ENSMUST00000068394
Chromosome	chr7	chr7	chr7	chr7	chr7
Strand	-	-	-	-	-
Start	64636960	64642091	64642181	64642296	64642439
Stop	64637055	64642118	64642278	64642382	64642568
P30.WT.CSMN	9.1	10.2	8.5	7.1	6.4
P30.SOD1.CSMN	9.2	10.9	9.4	8.3	6.7

Gabrp::TranscriptClusterID:6787196

Exon.ID	140189	140189	140190	140190	140193	140194	140195	140202	140203	140204	140206	140207	140207	140210
mRNA.Accession	NM_146017	NM_146017	NM_146017	NM_146017	NM_146017	NM_146017	NM_146017	NM_146017	NM_146017	NM_146017	NM_146017	NM_146017	NM_146017	NM_146017
Chromosome	chr11	chr11	chr11	chr11	chr11	chr11	chr11	chr11	chr11	chr11	chr11	chr11	chr11	chr11
Strand	-	-	-	-	-	-	-	-	-	-	-	-	-	-
Start	33450830	33451150	33452059	33452556	33454354	33454968	33456970	33463904	33467301	33468106	33472375	33473341	33473371	33478854
Stop	33450856	33451685	33452427	33452786	33454473	33455100	33457060	33463933	33467414	33468133	33472455	33473369	33473415	33478956
P30.WT.CSMN	1.5	5.8	4.7	3.9	5.1	3.1	4.3	2	6.5	5.7	3.5	2.1	5.7	4.2
P30.SOD1.CSMN	3	7.3	6.7	3.5	6.4	5.1	7	2.4	6.9	5.7	4.8	4.9	7	5.1

Gabrq::TranscriptClusterID:7011904

Exon.ID	909868	909868	909869	909870	909871	909871	909872	909873	909874	909875	909876
mRNA.Accession	ENSMUST00000033711	NM_020488	NM_020488	NM_020488	NM_020488	NM_020488	NM_020488	NM_020488	NM_020488	NM_020488	NM_020488
Chromosome	chrX	chrX	chrX	chrX	chrX	chrX	chrX	chrX	chrX	chrX	chrX
Strand	+	+	+	+	+	+	+	+	+	+	+
Start	70070596	70070800	70072917	70077000	70078547	70078578	70080451	70080997	70081500	70082170	70083150
Stop	70070702	70070937	70072976	70077054	70078571	70078716	70080509	70081099	70081634	70082378	70083299
P30.WT.CSMN	4.4	6.5	2	2.2	1	4.3	5.1	4.1	3.9	2	5.1
P30.SOD1.CSMN	6.2	6.9	3.7	4.6	2	4.6	5.1	3.8	5.9	3.6	6.1

Exon.ID	909876	909876	909876	909877	909877	909878
mRNA.Accession	NM_020488	NM_020488	NM_020488	ENSMUST00000114553	ENSMUST00000114553	ENSMUST00000114553
Chromosome	chrX	chrX	chrX	chrX	chrX	chrX
Strand	+	+	+	+	+	+
Start	70083491	70083740	70083821	70084848	70085035	70087733
Stop	70083656	70083812	70083870	70084903	70085541	70087843
P30.WT.CSMN	2.9	5	2.7	3.7	4.1	4.7
P30.SOD1.CSMN	4.8	4.5	4.7	5	6.3	5

Gabrr1::TranscriptClusterID:6912521

Exon.ID	573149	573149	573153	573154	573156	573157	573159	573161	573162	573163	573164	573164	573165	573165	573165
mRNA.Accession	NM_008075	NM_008075	NM_008075	NM_008075	NM_008075	NM_008075	NM_008075	NM_008075	NM_008075	NM_008075	NM_008075	NM_008075	NM_008075	NM_008075	NM_008075
Chromosome	chr4	chr4	chr4	chr4	chr4	chr4	chr4	chr4	chr4	chr4	chr4	chr4	chr4	chr4	chr4
Strand	+	+	+	+	+	+	+	+	+	+	+	+	+	+	+
Start	33219539	33219648	33233917	33235897	33238347	33239391	33244035	33245013	33247198	33248710	33249627	33249756	33249863	33250043	33250296
Stop	33219640	33219745	33233961	33235983	33238408	33239603	33244065	33245152	33247244	33248752	33249693	33249825	33250006	33250160	33250511
P30.WT.CSMN	4.3	1.7	7	1.8	5.4	2.8	2.6	3.8	5.5	5.3	4.7	5	4	3.2	4.3
P30.SOD1.CSMN	5.6	2.5	8.3	3.8	7.3	6.2	5.8	3.3	7.7	7.7	8.1	5.3	5.8	5.5	5.6

Gabrr2::TranscriptClusterID:6912520

Exon.ID	573120	573121	573122	573129	573130	573131	573134	573135	573136	573137
mRNA.Accession	ENSMUST00000108162	NM_008076	NM_008076	NM_008076	NM_008076	AK160446	NM_008076	NM_008076	NM_008076	NM_008076
Chromosome	chr4	chr4	chr4	chr4	chr4	chr4	chr4	chr4	chr4	chr4
Strand	+	+	+	+	+	+	+	+	+	+
Start	33149974	33150047	33150230	33158343	33164432	33165802	33168323	33169254	33169549	33171333
Stop	33150001	33150194	33150296	33158428	33164487	33165830	33168527	33169279	33169644	33171437
P30.WT.CSMN	2.8	5.5	3.6	6.4	4.9	5.8	4.5	3.4	4.1	6
P30.SOD1.CSMN	4.5	5.7	5.8	7.6	6.8	7.7	6.6	6.8	4.4	6.6

Exon.ID	573138	573140	573141	573143	573144	573144	573145	573145
mRNA.Accession	NM_008076	AK081227	AK081227	NM_008076	NM_008076	NM_008076	NM_008076	AK132642
Chromosome	chr4	chr4	chr4	chr4	chr4	chr4	chr4	chr4
Strand	+	+	+	+	+	+	+	+
Start	33172580	33178153	33180679	33182253	33182370	33182507	33182597	33182904
Stop	33172614	33179826	33180866	33182280	33182471	33182544	33182795	33183015
P30.WT.CSMN	6.6	2.9	4.9	3.8	3.9	3.4	4.3	2.2
P30.SOD1.CSMN	8.7	5.7	6.2	5.8	5.4	2	4.9	3.8

Gabrr3::TranscriptClusterID:6841879

Exon.ID	329836	329838	329841	329842	329843	329845	329846	329847	329848	329849
mRNA.Accession	NM_001081190	NM_001081190	NM_001081190	NM_001081190	NM_001081190	NM_001081190	NM_001081190	NM_001081190	NM_178751	NM_001081190
Chromosome	chr16	chr16	chr16	chr16	chr16	chr16	chr16	chr16	chr16	chr16
Strand	+	+	+	+	+	+	+	+	+	+
Start	59407206	59415113	59426247	59429805	59433269	59434394	59440327	59447770	59457615	59461239
Stop	59407316	59415179	59426309	59429965	59433348	59434497	59440435	59447893	59458050	59461672
P30.WT.CSMN	7.4	4.4	5.2	4.4	3.3	4.5	4.2	4.5	6.1	2.2
P30.SOD1.CSMN	7.3	5.6	4.7	5.3	6.6	4.3	6.7	6.7	8.8	4.4

Gabrr3::TranscriptClusterID:6841881

Exon.ID	329852	329853
mRNA.Accession	AK141642	AK141642
Chromosome	chr16	chr16
Strand	+	+
Start	59463307	59464048
Stop	59463824	59464134
P30.WT.CSMN	3.2	3.3
P30.SOD1.CSMN	5.9	5.1

Kcna1::TranscriptClusterID:6957244

Exon.ID	728577
mRNA.Accession	NM_010595
Chromosome	chr6
Strand	-
Start	126586746
Stop	126587458
P30.WT.CSMN	10.4
P30.SOD1.CSMN	10.9

Kcna1::TranscriptClusterID:6957245

Exon.ID	728578
mRNA.Accession	NM_010595
Chromosome	chr6
Strand	-
Start	126588835
Stop	126589152
P30.WT.CSMN	10.6
P30.SOD1.CSMN	11.1

Kcna1::TranscriptClusterID:6957246

Exon.ID	728579	728579	728579	728579	728579	728579	728579	728579	728579	728579	728580	728580
mRNA.Accession	NM_010595	NM_010595	NM_010595	NM_010595	NM_010595	Y00305	NM_010595	NM_010595	NM_010595	NM_010595	NM_010595	NM_010595
Chromosome	chr6	chr6	chr6	chr6	chr6	chr6	chr6	chr6	chr6	chr6	chr6	chr6
Strand	-	-	-	-	-	-	-	-	-	-	-	-
Start	126589601	126591887	126593474	126593803	126593875	126593944	126594319	126594425	126594494	126594740	126595178	126595748
Stop	126591833	126593269	126593792	126593874	126593904	126594267	126594395	126594454	126594666	126594814	126595683	126595810
P30.WT.CSMN	10.4	11.8	9.9	10.2	8.1	8.2	9.8	9.7	8.2	7	6.6	6.9
P30.SOD1.CSMN	11.3	12.4	11	11.3	9.4	9.1	11	10.5	8.9	8.2	8.1	8.4

Kcna2::TranscriptClusterID:6900287

Exon.ID	533954	533956	533956	533957	533958	533958	533962	533963	533963	533964	533965
mRNA.Accession	NM_008417	NM_008417	NM_008417	NM_008417	NM_008417	NM_008417	AK140299	AK140299	AK140299	AK140299	AK140299
Chromosome	chr3	chr3	chr3	chr3	chr3	chr3	chr3	chr3	chr3	chr3	chr3
Strand	+	+	+	+	+	+	+	+	+	+	+
Start	106904522	106906102	106906303	106906868	106907070	106908751	106913900	106916780	106916810	106917217	106917692
Stop	106904627	106906132	106906413	106906978	106907929	106909756	106916529	106916808	106916872	106917296	106917827
P30.WT.CSMN	8.6	8.7	9.5	10.8	10.8	10.3	9.2	9.4	10.1	9.1	6.3
P30.SOD1.CSMN	10.2	9.4	9.9	11.7	11.4	11	10.5	10.8	11.2	10.5	7.9

Kcna3::TranscriptClusterID:6900277

Exon.ID	533937	533937	533937	533937
mRNA.Accession	NM_008418	NM_008418	NM_008418	NM_008418
Chromosome	chr3	chr3	chr3	chr3
Strand	+	+	+	+
Start	106839085	106839348	106839510	106839612
Stop	106839315	106839472	106839601	106840615
P30.WT.CSMN	7.8	8	6.9	6.7
P30.SOD1.CSMN	9.1	10	8.5	8.4

Kcna4::TranscriptClusterID:6879833

Exon.ID	462919	462919	462919	462920	462920	462920	462920	462920	462920	462920	462920	462920
mRNA.Accession	NM_021275	NM_021275	NM_021275	NM_021275	NM_021275	NM_021275	NM_021275	NM_021275	NM_021275	NM_021275	NM_021275	NM_021275
Chromosome	chr2	chr2	chr2	chr2	chr2	chr2	chr2	chr2	chr2	chr2	chr2	chr2
Strand	+	+	+	+	+	+	+	+	+	+	+	+
Start	107130804	107130989	107131146	107134320	107134709	107134843	107135111	107135499	107135568	107135616	107135705	107137665
Stop	107130899	107131083	107131228	107134660	107134760	107134991	107135320	107135541	107135610	107135677	107136769	107138506
P30.WT.CSMN	6	7.3	4.5	5.5	7.6	5.2	4.9	5.4	5.5	6.5	6.9	8.9
P30.SOD1.CSMN	6.3	8.1	5.6	6.4	8.8	6	6.3	5.4	7.4	8.6	7.7	9.6

Kcna5::TranscriptClusterID:6957240

Exon.ID	728542	728542	728542
mRNA.Accession	NM_145983	NM_145983	NM_145983
Chromosome	chr6	chr6	chr6
Strand	-	-	-
Start	126482771	126483657	126485252
Stop	126483335	126485073	126485351
P30.WT.CSMN	5.6	4.6	4.8
P30.SOD1.CSMN	7.1	6.9	6.6

Kcna6::TranscriptClusterID:6957248

Exon.ID	728589	728590	728590	728590	728591	728600	728601	728601	728601	728601	728601	728603	728603	728603	728603
mRNA.Accession	AK148678	AK148678	AK148678	AK148678	ENSMUST00000040751	AK134477	ENSMUST00000040751	NM_013568	NM_013568	NM_013568	NM_013568	NM_013568	NM_013568	NM_013568	M96688
Chromosome	chr6	chr6	chr6	chr6	chr6	chr6	chr6	chr6	chr6	chr6	chr6	chr6	chr6	chr6	chr6
Strand	-	-	-	-	-	-	-	-	-	-	-	-	-	-	-
Start	126655485	126657807	126658160	126658221	126658376	126677510	126685454	126686748	126687916	126688024	126688378	126689177	126689237	126689562	126690205
Stop	126655618	126658059	126658214	126658330	126658691	126677579	126686699	126687689	126687966	126688280	126688605	126689218	126689421	126689763	126690518
P30.WT.CSMN	5.7	7.3	5.7	4.7	7.5	6.7	6.3	9.7	9.3	7.8	7.5	7.6	7.9	8.8	7.6
P30.SOD1.CSMN	6.3	6.1	7.3	5.2	8.6	5.5	7.6	10.8	10.5	8.8	8.5	7	8.3	9.8	8.4

Kcna7::TranscriptClusterID:6960370

Exon.ID	739644	739645	739645	739646	739646	739647	739647	739647	739647	739647
mRNA.Accession	NM_010596	NM_010596	NM_010596	NM_010596	NM_010596	NM_010596	NM_010596	NM_010596	NM_010596	NM_010596
Chromosome	chr7	chr7	chr7	chr7	chr7	chr7	chr7	chr7	chr7	chr7
Strand	+	+	+	+	+	+	+	+	+	+
Start	52661351	52661605	52661732	52664444	52664937	52665067	52665448	52665875	52666033	52666564
Stop	52661576	52661669	52662331	52664704	52665021	52665118	52665576	52665910	52666200	52666611
P30.WT.CSMN	5.9	5.2	6.5	5.2	7.2	7.5	5.4	3.8	4.1	5.6
P30.SOD1.CSMN	6.4	7	7.1	6.3	7.7	7.5	6.6	5.4	5.9	6.6

Kcna10::TranscriptClusterID:6900290

Exon.ID	533990	533993	533994	533994
mRNA.Accession	NM_001081140	NM_001081140	NM_001081140	NM_001081140
Chromosome	chr3	chr3	chr3	chr3
Strand	+	+	+	+
Start	106986001	106996917	106997429	106998573
Stop	106986154	106996944	106997825	106998600
P30.WT.CSMN	4.3	5.8	5.9	1.6
P30.SOD1.CSMN	5.4	7.1	6.3	2.6

Kcnab1::TranscriptClusterID:6898063

Exon.ID	525995	526008	526010	526010	526016	526017	526020	526021	526029	526030	526033	526034	526042
mRNA.Accession	AK035994	AK149120	ENSMUST00000049230	NM_010597	AK157654	AK157654	AK031381	AK031381	AK153200	AK153200	AK035994	AK035994	AK015412
Chromosome	chr3	chr3	chr3	chr3	chr3	chr3	chr3	chr3	chr3	chr3	chr3	chr3	chr3
Strand	+	+	+	+	+	+	+	+	+	+	+	+	+
Start	64753208	64880438	64912997	64913743	64928985	64931788	64954087	64956286	64992080	64992909	65025849	65026495	65065510
Stop	64753430	64880638	64913427	64913911	64929168	64932025	64954485	64956757	64992152	64992946	65026021	65027589	65065652
P30.WT.CSMN	5.3	3.2	7.2	9.8	5.6	6.4	6	6.1	2.9	3.1	7.2	6.6	2.9
P30.SOD1.CSMN	6.4	4.9	7.4	9.9	5	5.6	5.3	5.2	5.5	5.5	6	5.8	2.3

Exon.ID	526042	526044	526052	526056	526061	526062	526063	526066	526073	526073	526074	526074
mRNA.Accession	ENSMUST00000029413	NM_010597	NM_010597	NM_010597	NM_010597	NM_010597	NM_010597	NM_010597	AK082310	NM_010597	NM_010597	AK082310
Chromosome	chr3	chr3	chr3	chr3	chr3	chr3	chr3	chr3	chr3	chr3	chr3	chr3
Strand	+	+	+	+	+	+	+	+	+	+	+	+
Start	65065663	65070431	65099187	65101592	65103516	65106071	65108050	65123357	65158066	65160187	65160955	65161750
Stop	65065753	65070455	65099224	65101651	65103551	65106096	65108074	65123411	65160175	65160232	65161063	65162118
P30.WT.CSMN	6.5	8	9.1	8.3	8.6	8.4	8.9	9.3	7.2	7.2	8.4	3.5
P30.SOD1.CSMN	4.9	8.1	9.3	8.4	9.1	9	9	9.9	7.1	7.5	9.1	6.3

Exon.ID	526074	526074	526074	526074	526078	526079	526083	526087	526087
mRNA.Accession	NM_010597	AK082310	AK082310	AK082310	NM_010597	AK031362	NM_010597	NM_010597	NM_010597
Chromosome	chr3	chr3	chr3	chr3	chr3	chr3	chr3	chr3	chr3
Strand	+	+	+	+	+	+	+	+	+
Start	65162214	65162333	65162417	65162479	65168535	65169261	65175291	65180409	65181031
Stop	65162254	65162360	65162447	65162728	65168633	65169632	65175356	65180470	65181600
P30.WT.CSMN	10.3	5.8	6.7	5.6	8.3	7.6	10	9.5	9.6
P30.SOD1.CSMN	10.5	7.4	8.7	5.9	8	7.5	10.4	9.8	9.7

Kcnab2::TranscriptClusterID:6927145

Exon.ID	623468	623468	623469	623470	623471	623472	623473	623475	623476	623477	623478	623479	623481	623482	623489
mRNA.Accession	NM_010598	NM_010598	NM_010598	NM_010598	NM_010598	NM_010598	NM_010598	NM_010598	NM_010598	NM_010598	NM_010598	NM_010598	NM_010598	NM_010598	AK037354
Chromosome	chr4	chr4	chr4	chr4	chr4	chr4	chr4	chr4	chr4	chr4	chr4	chr4	chr4	chr4	chr4
Strand	-	-	-	-	-	-	-	-	-	-	-	-	-	-	-
Start	151765332	151767162	151767852	151768412	151769055	151769273	151770883	151775061	151776034	151777145	151778710	151781248	151786094	151786927	151799248
Stop	151766838	151767226	151767925	151768478	151769130	151769350	151770907	151775111	151776073	151777188	151778751	151781314	151786131	151786962	151799463
P30.WT.CSMN	9.4	10.3	9.7	9.3	9.6	8	9.5	8.6	8.3	7.6	9.3	10.4	11	10	8
P30.SOD1.CSMN	11	11.4	11.1	10.5	10.8	9.2	10.7	10.4	9.1	9.2	10.4	11.6	12.1	11	8.1

Exon.ID	623490	623491	623491	623492	623496	623497	623497	623499	623501
mRNA.Accession	AK037354	AK037354	AK037354	AK037354	NM_010598	NM_010598	NM_010598	ENSMUST00000105648	XM_001473856
Chromosome	chr4	chr4	chr4	chr4	chr4	chr4	chr4	chr4	chr4
Strand	-	-	-	-	-	-	-	-	-
Start	151799717	151800464	151800637	151802089	151808908	151809965	151809992	151822801	151828994
Stop	151799944	151800570	151800766	151802392	151808932	151809990	151810026	151822912	151829095
P30.WT.CSMN	5.7	5.9	7.4	7.2	9.4	9.9	9.5	6	6.1
P30.SOD1.CSMN	6.4	5.7	6.5	6.5	9.1	10.5	10.4	6.5	7.7

Kcnab3::TranscriptClusterID:6782002

Exon.ID	120599	120599	120599	120600	120600	120602	120602	120602	120603	120605	120606	120607	120608	120608
mRNA.Accession	NM_010599	NM_010599	NM_010599	NM_010599	NM_010599	AK029750	NM_010599	NM_010599	NM_010599	NM_010599	NM_010599	NM_010599	U65593	NM_010599
Chromosome	chr11	chr11	chr11	chr11	chr11	chr11	chr11	chr11	chr11	chr11	chr11	chr11	chr11	chr11
Strand	+	+	+	+	+	+	+	+	+	+	+	+	+	+
Start	69139762	69139865	69139940	69140190	69140321	69141410	69141631	69141755	69142026	69143353	69143485	69143688	69143826	69143914
Stop	69139848	69139934	69140116	69140229	69140410	69141602	69141664	69141787	69142088	69143392	69143516	69143725	69143860	69143985
P30.WT.CSMN	6.1	6.4	6.8	7.2	6.6	5.6	8.8	7.4	7.6	5.4	6.9	8	4.8	6.6
P30.SOD1.CSMN	7.8	7.4	8	8.6	8.6	5.1	10.6	9	8.2	7.6	6.7	9	3.6	7.5

Exon.ID	120609	120610	120611	120612	120613	120614	120614
mRNA.Accession	NM_010599	NM_010599	NM_010599	NM_010599	NM_010599	NM_010599	NM_010599
Chromosome	chr11	chr11	chr11	chr11	chr11	chr11	chr11
Strand	+	+	+	+	+	+	+
Start	69144242	69144565	69144794	69145019	69145243	69145589	69145837
Stop	69144309	69144626	69144856	69145082	69145304	69145645	69146427
P30.WT.CSMN	6.4	6.1	7.8	7.6	10	8.7	7
P30.SOD1.CSMN	7.5	8.1	9.3	9.3	11.7	10.1	8.5

Kcnb1::TranscriptClusterID:6893131

Exon.ID	511208	511208	511216	511218	511219	511220
mRNA.Accession	NM_008420	NM_008420	BC095991	NM_008420	NM_008420	DQ646376
Chromosome	chr2	chr2	chr2	chr2	chr2	chr2
Strand	-	-	-	-	-	-
Start	166929195	166929934	167009207	167013566	167014161	167015576
Stop	166929844	166931687	167009276	167014068	167014280	167015903
P30.WT.CSMN	10	11	7	10.5	8.1	4.6
P30.SOD1.CSMN	11	11.8	7.4	11.5	9	5.5

Kcnb2::TranscriptClusterID:6747766

Exon.ID	1578	1592	1605	1606	1611	1616	1628	1628	1628	1628	1629	1630	1633	1633	1634	1635
mRNA.Accession	NM_001098528	AK051181	AK086148	AK086148	AK020510	AK049996	NM_001098528	NM_001098528	NM_001098528	NM_001098528	NM_001098528	NM_001098528	AK081595	AK081595	AK081595	AK081595
Chromosome	chr1	chr1	chr1	chr1	chr1	chr1	chr1	chr1	chr1	chr1	chr1	chr1	chr1	chr1	chr1	chr1
Strand	+	+	+	+	+	+	+	+	+	+	+	+	+	+	+	+
Start	15302717	15354723	15546373	15547809	15560994	15576487	15699571	15700743	15700926	15701694	15702754	15703811	15706997	15707334	15707840	15708513
Stop	15303110	15355395	15546408	15548398	15561162	15576820	15700502	15700814	15701326	15701896	15702864	15704239	15707079	15707494	15707946	15708802
P30.WT.CSMN	8.1	7.2	2.6	5.9	6.4	6.6	7.9	8.3	8.2	6.3	7.3	6.8	6.4	5.8	5.7	7.1
P30.SOD1.CSMN	8.3	7.7	3.4	6.1	5.7	6.5	8.8	8.7	9	7	6.5	7.4	6.6	6.1	6.2	8.2

Kcnc1::TranscriptClusterID:6960456

Exon.ID	740088	740088	740088	740088	740100	740100	740100	740101	740102	740103	740104	740105	740106	740107	740107
mRNA.Accession	NM_001112739	NM_001112739	NM_001112739	NM_001112739	NM_001112739	NM_008421	NM_008421	NM_008421	ENSMUST00000025202	NM_001112739	ENSMUST00000025202	ENSMUST00000025202	ENSMUST00000025202	NM_001112739	NM_001112739
Chromosome	chr7	chr7	chr7	chr7	chr7	chr7	chr7	chr7	chr7	chr7	chr7	chr7	chr7	chr7	chr7
Strand	+	+	+	+	+	+	+	+	+	+	+	+	+	+	+
Start	53652226	53653193	53653418	53653548	53682878	53683650	53683703	53684308	53690290	53690507	53690727	53690924	53691781	53692666	53693138
Stop	53652838	53653277	53653526	53653595	53683460	53683681	53683903	53684558	53690429	53690684	53690758	53691237	53692481	53692792	53694007
P30.WT.CSMN	7.2	6.6	6.3	6.8	9.5	6.6	6.6	6.6	3.6	6.5	5.6	4.3	5.9	6.5	5.5
P30.SOD1.CSMN	9.1	8.9	8.8	9.1	12	7.9	8.1	8.8	6.2	9	7.3	6.9	8.2	9.9	7.5

Kcnc1::TranscriptClusterID:6960457

Exon.ID	740108	740109	740109	740109	740109	740109	740109	740109	740109
mRNA.Accession	NM_008421	NM_008421	NM_008421	NM_008421	NM_008421	NM_008421	NM_008421	NM_008421	NM_008421
Chromosome	chr7	chr7	chr7	chr7	chr7	chr7	chr7	chr7	chr7
Strand	+	+	+	+	+	+	+	+	+
Start	53684688	53684995	53685100	53687317	53687543	53687693	53687856	53688404	53688466
Stop	53684825	53685020	53686678	53687501	53687672	53687770	53688269	53688459	53688644
P30.WT.CSMN	7.6	7.4	8	7.1	5.2	6.1	5.9	4.7	5.3
P30.SOD1.CSMN	8.5	8.7	9.1	9.1	7.8	7.4	8.1	5.5	6.7

Kcnc2::TranscriptClusterID:6770751

Exon.ID	82119	82120	82121	82121	82121	82121	82121	82121	82140	82145	82148	82148	
mRNA.Accession	NM_001025581	NM_001025581	NM_001025581	NM_001025581	NM_001025581	NM_001025581	NM_001025581	NM_001025581	AK140994	AK042937	AK045833	AK045833	A
Chromosome	chr10	chr10	chr10	chr10	chr10	chr10	chr10	chr10	chr10	chr10	chr10	chr10	
Strand	+	+	+	+	+	+	+	+	+	+	+	+	
Start	111708183	111708762	111708895	111708944	111709000	111709065	111709315	111709401	111786124	111813972	111825168	111825374	1
Stop	111708207	111708874	111708942	111708974	111709037	111709292	111709389	111709436	111786640	111814036	111825310	111825413	1
P30.WT.CSMN	6.5	7.6	8.1	11.1	5.2	5.2	6.3	6.1	3.2	3.8	4.5	4.1	
P30.SOD1.CSMN	8	9.7	9.8	12.4	7	8.5	8.8	8.9	5.6	4.9	5.7	4.2	

Exon.ID	82149	82158	82158	82159	82160	82160	82163	82164	82164	82164
mRNA.Accession	AK045833	NM_001025581	NM_001025581	AK034053	NM_001025581	NM_001025581	NM_001025581	NM_001025581	NM_001025581	AK034053
Chromosome	chr10	chr10	chr10	chr10	chr10	chr10	chr10	chr10	chr10	chr10
Strand	+	+	+	+	+	+	+	+	+	+
Start	111826786	111892782	111893354	111894008	111895546	111895599	111899035	111900393	111902322	111903596
Stop	111826933	111893083	111893511	111894083	111895596	111895699	111899737	111902301	111902772	111903980
P30.WT.CSMN	2.2	5.9	6.9	4.2	5.8	5.8	4.8	6	5.2	2.4
P30.SOD1.CSMN	5.9	8.5	8.4	6.8	8.9	9.1	7.6	9.3	8.6	4.7

Kcnc2::TranscriptClusterID:6778257

Exon.ID	108078	108078	108077
mRNA.Accession	NM_001025581	NM_001025581	AK034053
Chromosome	chr10	chr10	chr10
Strand	+	+	+
Start	111892690	111892782	111894008
Stop	111892721	111893511	111894083
P30.WT.CSMN	6.4	5.3	4.2
P30.SOD1.CSMN	8.9	8	7.2

Kcnc3::TranscriptClusterID:6960291

Exon.ID	739322	739322	739322	739322	739322	739325	739325	739326	739326	739326	739327	739330	739332
mRNA.Accession	NM_008422	NM_008422	NM_008422	NM_008422	NM_008422	BC030422	BC030422	BC030422	NM_008422	NM_008422	NM_008422	ENSMUST00000107905	NM_008422
Chromosome	chr7	chr7	chr7	chr7	chr7	chr7	chr7	chr7	chr7	chr7	chr7	chr7	chr7
Strand	+	+	+	+	+	+	+	+	+	+	+	+	+
Start	51846010	51846490	51846550	51846624	51847072	51849309	51849484	51850468	51850798	51851593	51853778	51856247	51858451
Stop	51846281	51846528	51846591	51847004	51847127	51849434	51849509	51850504	51851306	51851638	51853908	51856273	51859107
P30.WT.CSMN	6.8	13.8	7.1	7.7	9.9	7.3	5.7	6.7	10.4	6.9	8.6	9.2	9.1
P30.SOD1.CSMN	8.4	14.3	7.8	8.6	10.8	8.1	5.9	6.9	11.6	7.9	9.9	11.3	10.6

Kcnc4::TranscriptClusterID:6908029

Exon.ID	559050	559051	559052	559053	559054	559054	559055	559058	559059	559062	559064	559065	559065	559065
mRNA.Accession	NM_145922	NM_145922	AK039581	AK039581	AK039581	AK039581	AK039581	NM_145922	NM_145922	AK077483	NM_145922	NM_145922	NM_145922	NM_145922
Chromosome	chr3	chr3	chr3	chr3	chr3	chr3	chr3	chr3	chr3	chr3	chr3	chr3	chr3	chr3
Strand	-	-	-	-	-	-	-	-	-	-	-	-	-	-
Start	107241252	107242348	107243134	107243903	107244065	107244425	107245407	107248364	107250894	107255446	107261124	107261485	107261602	107261727
Stop	107242280	107242388	107243892	107243939	107244289	107244769	107245869	107248533	107251134	107258679	107261407	107261590	107261682	107261792
P30.WT.CSMN	7	8.3	4.7	8.8	4.7	7.2	6.3	7.8	10.3	5.8	7.9	8.2	8.3	6.2
P30.SOD1.CSMN	8.5	9.2	6.4	10	6.5	8	7.7	10	11.7	5.9	9.2	9.3	8.8	7.1

Kcnd1::TranscriptClusterID:7009791

Exon.ID	903608	903608	903609	903609	903609	903610	903610	903611	903611	903612	903612	903613
mRNA.Accession	XM_001473739	XM_001473739	XM_001473739	XM_001473739	XM_001473739	XM_001473739	XM_001473739	BC139325	BC139325	NM_008423	NM_008423	NM_008423
Chromosome	chrX	chrX	chrX	chrX	chrX	chrX	chrX	chrX	chrX	chrX	chrX	chrX
Strand	+	+	+	+	+	+	+	+	+	+	+	+
Start	7399279	7399448	7399822	7399937	7400048	7400166	7400397	7400897	7400943	7400977	7401226	7408311
Stop	7399388	7399498	7399876	7400047	7400152	7400231	7400655	7400924	7400967	7401006	7401638	7408349
P30.WT.CSMN	6.1	3.9	7.4	5.9	7.3	7.7	8.5	5.6	10.2	2.3	6.3	5.7
P30.SOD1.CSMN	7.4	6.1	8.2	6.5	7.6	8.4	8.7	7.2	11.7	5.2	7.4	7.3

	903614	903615	903616	903617	903618	903618	903619	903620	903620
	NM_008423	NM_008423	NM_008423	NM_008423	NM_008423	NM_008423	ENSMUST00000009875	ENSMUST00000009875	ENSMUST00000009875
	chrX	chrX	chrX	chrX	chrX	chrX	chrX	chrX	chrX
	+	+	+	+	+	+	+	+	+
	7408435	7408562	7408736	7409239	7413415	7413590	7413648	7414766	7414813
	7408460	7408618	7408818	7409456	7413581	7413625	7414703	7414793	7415237
	7.3	7.5	7.5	7.8	7.8	3	5.3	1.6	4.1
	8.9	7.9	8.8	8.6	9.3	5.6	6.9	2.2	6.3

Kcnd2::TranscriptClusterID:6944514

Exon.ID	685439	685439	685439	685439	685440	685444	685445	685446	685448	685449	685451	685454	685455	685456	685457	685458	685459
mRNA.Accession	AK032268	AB045326	AB045326	NM_019697	NM_019697	AK032319	AK032319	AK032319	AK086084	AK086084	AK034048	AK043453	AK043453	AK082100	AK082100	AK082100	AK082100
Chromosome	chr6	chr6	chr6	chr6	chr6	chr6	chr6	chr6	chr6	chr6	chr6	chr6	chr6	chr6	chr6	chr6	chr6
Strand	+	+	+	+	+	+	+	+	+	+	+	+	+	+	+	+	+
Start	21165196	21165538	21165739	21165973	21166618	21183695	21184647	21185366	21196199	21197422	21201892	21209139	21209829	21211826	21213250	21213683	21215045
Stop	21165413	21165711	21165805	21166172	21167067	21183808	21185025	21185723	21196623	21197892	21202067	21209298	21210026	21212333	21213443	21213880	21215434
P30.WT.CSMN	7.4	7.1	7.7	9.3	10	6.8	5.3	4.9	7.3	9.6	5.6	6.9	8.4	3.8	5.7	5.6	6.1
P30.SOD1.CSMN	7.9	8	8.8	10.3	10.8	6.5	5.3	5.5	6.6	9.6	6.2	7	7.8	6.2	5	5.3	5.7

	685465	685466	685467	685472	685480	685481	685484	685488	685489	685490	685503	685504	685511	685514	685515	685516	685517	685518
	AK140700	AK140700	AK140700	AK135459	AK144305	AK144305	AK048823	AK047539	AK047539	AK047539	AK042949	AK042949	NM_019697	NM_019697	NM_019697	NM_019697	NM_019697	NM_019697
	chr6	chr6	chr6	chr6	chr6	chr6	chr6	chr6	chr6	chr6	chr6	chr6	chr6	chr6	chr6	chr6	chr6	chr6
	+	+	+	+	+	+	+	+	+	+	+	+	+	+	+	+	+	+
	21257449	21259603	21260758	21387134	21448861	21450559	21463032	21490985	21492140	21492943	21631451	21633993	21664158	21672394	21673189	21676016	21677208	21677369
	21257670	21259761	21261055	21387453	21449058	21450919	21463212	21491304	21492167	21493407	21631860	21634030	21664269	21672474	21673234	21676155	21677338	21679213
	8.4	8.1	8.1	4.8	7.4	8.7	7.1	6.4	4.3	7	4.1	3.6	9.7	9.2	9.3	10.5	8.7	8.9
	7.2	7.7	6.2	3.3	6.5	8.6	6.9	6.8	3.8	6	4.5	2.7	10.3	10	9.9	11.6	9.3	10

Kcnd3::TranscriptClusterID:6900214

Exon.ID	533649	533650	533651	533651	533701	533705	533706	533706	533706	533706	533706	533707	533708	533709	533710
mRNA.Accession	NM_001039347	ENSMUST00000118360	NM_001039347	NM_001039347	NM_001039347	NM_001039347	NM_001039347	AK033962	AK033962	NM_019931	AK033962	NM_001039347	NM_001039347	NM_001039347	NM_001039347
Chromosome	chr3	chr3	chr3	chr3	chr3	chr3	chr3	chr3	chr3	chr3	chr3	chr3	chr3	chr3	chr3
Strand	+	+	+	+	+	+	+	+	+	+	+	+	+	+	+
Start	105254879	105256040	105261657	105262034	105461532	105467948	105468390	105468509	105468639	105469886	105470200	105471034	105471934	105472240	105475070
Stop	105255478	105256335	105261726	105262508	105461663	105468017	105468454	105468585	105469405	105469926	105470468	105471218	105472110	105472354	105476646
P30.WT.CSMN	6.2	5.5	6.7	9.2	9.3	10	9.4	6.8	5.4	7.8	7.8	9.5	7.1	6.3	8.9
P30.SOD1.CSMN	8.1	6.4	6.3	9.6	9.8	10.3	10.3	5.1	6.1	7.5	7	10.2	8.1	7.6	9.7

Kcnd3::TranscriptClusterID:6907903

Exon.ID	558614	558617	558619	558619	558619	558619	558620	558620
mRNA.Accession	AK018929	ENSMUST00000098762	ENSMUST00000098762	AK018929	ENSMUST00000098762	AK018929	NM_001039347	ENSMUST00000098762
Chromosome	chr3	chr3	chr3	chr3	chr3	chr3	chr3	chr3
Strand	-	-	-	-	-	-	-	-
Start	105244535	105251261	105254092	105254352	105254450	105254606	105255335	105255666
Stop	105244593	105251311	105254325	105254391	105254575	105254634	105255468	105256094
P30.WT.CSMN	4.7	4.4	7.5	4	4.7	6.4	7.5	6.9
P30.SOD1.CSMN	4.5	4.1	7.4	4.6	6.2	5.7	9	8.4

Kcne1::TranscriptClusterID:6847948

Exon.ID	349477	349478	349479	349479	349479	349479	349482	349483
mRNA.Accession	NM_008424	NM_008424	NM_008424	NM_008424	NM_008424	NM_008424	X60457	NM_008424
Chromosome	chr16	chr16	chr16	chr16	chr16	chr16	chr16	chr16
Strand	-	-	-	-	-	-	-	-
Start	92346409	92347223	92347584	92348855	92349162	92349200	92359080	92359572
Stop	92346556	92347248	92348774	92349071	92349199	92349236	92359105	92359682
P30.WT.CSMN	5.1	2.1	5.1	4.8	8.2	5.1	5.6	5.5
P30.SOD1.CSMN	5.9	4.6	5.9	6.3	8	6.5	5.4	7

Kcne2::TranscriptClusterID:6843216

Exon.ID	333742	333743	333745	333746	333746
mRNA.Accession	NM_134110	ENSMUST00000113971	NM_134110	NM_134110	NM_134110
Chromosome	chr16	chr16	chr16	chr16	chr16
Strand	+	+	+	+	+
Start	92292649	92295872	92296950	92297223	92297581
Stop	92292705	92295937	92297161	92297405	92298179
P30.WT.CSMN	4.3	5.7	4.8	5.5	5.5
P30.SOD1.CSMN	6	7.8	7.3	6.1	5.8

Kcne3::TranscriptClusterID:6962922

Exon.ID	748686	748687	748687	748688	748690	748692	748692	748692	748693	748695	748696	748696
mRNA.Accession	AK132773	AK132773	AK132773	NM_020574	NM_020574	DQ832280	NM_020574	DQ832280	DQ832280	NM_020574	NM_020574	NM_020574
Chromosome	chr7	chr7	chr7	chr7	chr7	chr7	chr7	chr7	chr7	chr7	chr7	chr7
Strand	+	+	+	+	+	+	+	+	+	+	+	+
Start	107325203	107326504	107326639	107327225	107329675	107331207	107331243	107331334	107331526	107332658	107332810	107333051
Stop	107325292	107326536	107326750	107327275	107329737	107331239	107331333	107331490	107331582	107332685	107332942	107333536
P30.WT.CSMN	4.4	1.7	4.7	3.1	3.4	5.3	2	2.6	5.6	4.2	5.5	4.1
P30.SOD1.CSMN	6.5	2.2	5.7	4.6	4.4	6.3	3.9	4.2	6.2	4.9	6.9	5.8

Kcne4::TranscriptClusterID:6750872

Exon.ID	12328	12329	12329	12329	12329	12329	12329	12329	12329
mRNA.Accession	NM_021342	NM_021342	NM_021342	NM_021342	NM_021342	NM_021342	NM_021342	NM_021342	NM_021342
Chromosome	chr1	chr1	chr1	chr1	chr1	chr1	chr1	chr1	chr1
Strand	+	+	+	+	+	+	+	+	+
Start	78813443	78814186	78814270	78814460	78814778	78815073	78815455	78815953	78816368
Stop	78813551	78814211	78814382	78814709	78814955	78815103	78815930	78816061	78816523
P30.WT.CSMN	4.3	6.9	5.3	4.2	5.8	3.4	4.8	5.5	4.7
P30.SOD1.CSMN	3.4	9.1	6	6.5	7	3.3	7	5.1	6

Kcnf1::TranscriptClusterID:6793663

Exon.ID	165041
mRNA.Accession	AK043920
Chromosome	chr12
Strand	+
Start	17182531
Stop	17182794
P30.WT.CSMN	7.2
P30.SOD1.CSMN	8.1

Kcnf1::TranscriptClusterID:6793664

Exon.ID	165042
mRNA.Accession	AK043920
Chromosome	chr12
Strand	+
Start	17184179
Stop	17184573
P30.WT.CSMN	5.6
P30.SOD1.CSMN	7.2

Kcnf1::TranscriptClusterID:6799239

Exon.ID	184491	184491	184491	184491	184491	184491	184491
mRNA.Accession	NM_201531	NM_201531	NM_201531	NM_201531	NM_201531	NM_201531	NM_201531
Chromosome	chr12	chr12	chr12	chr12	chr12	chr12	chr12
Strand	-	-	-	-	-	-	-
Start	17179027	17179140	17179956	17181257	17181507	17182989	17183245
Stop	17179126	17179197	17181056	17181475	17182815	17183056	17183499
P30.WT.CSMN	8.2	8.7	8.4	9.3	9.2	7.9	6.8
P30.SOD1.CSMN	9.4	9.4	9.2	10.1	9.9	9	6.5

Kcng1::TranscriptClusterID:6893276

Exon.ID	511584
mRNA.Accession	ENSMUST00000109191
Chromosome	chr2
Strand	-
Start	168085658
Stop	168086083
P30.WT.CSMN	9.3
P30.SOD1.CSMN	9.8

Kcng1::TranscriptClusterID:6893277

Exon.ID	511585	511587	511587	511591
mRNA.Accession	NM_001081134	NM_001081134	NM_001081134	ENSMUST00000109191
Chromosome	chr2	chr2	chr2	chr2
Strand	-	-	-	-
Start	168087073	168094416	168094679	168106406
Stop	168087497	168094648	168094723	168106543
P30.WT.CSMN	9.8	10.1	8.9	8.1
P30.SOD1.CSMN	9.7	10.4	9.1	8.6

Kcng2::TranscriptClusterID:6866931

Exon.ID	416432	416432	416432	416432
mRNA.Accession	AK158780	AK158780	AK158780	AK158780
Chromosome	chr18	chr18	chr18	chr18
Strand	-	-	-	-
Start	80491304	80491373	80491546	80491731
Stop	80491355	80491478	80491667	80491760
P30.WT.CSMN	3.9	4.3	6	6.3
P30.SOD1.CSMN	6.2	6	7.5	7

Kcng2::TranscriptClusterID:6866932

Exon.ID	416433	416433	416433	416433	416433	416436	416438	416446	416446	416446	416446
mRNA.Accession	AK158780	AK158780	AK158780	AK158780	AK158780	AK136256	AK136002	XM_140499	XM_140499	XM_140499	XM_140499
Chromosome	chr18	chr18	chr18	chr18	chr18	chr18	chr18	chr18	chr18	chr18	chr18
Strand	-	-	-	-	-	-	-	-	-	-	-
Start	80492141	80492208	80492262	80492299	80492443	80499787	80502274	80519219	80519399	80519513	80519754
Stop	80492170	80492255	80492287	80492349	80493147	80500022	80502562	80519344	80519461	80519669	80519901
P30.WT.CSMN	3.4	6.9	7.3	7.2	7.6	4.8	5.2	5.8	9.8	6.6	3.9
P30.SOD1.CSMN	2	7.6	7.9	8.3	7.9	6.9	5.8	7.7	10.9	7.4	7.1

Kcng3::TranscriptClusterID:6857763

Exon.ID	385042	385043	385043	385043	385043	385055	385055	385055
mRNA.Accession	NM_153512	NM_153512	NM_153512	NM_153512	NM_153512	NM_153512	NM_153512	NM_153512
Chromosome	chr17	chr17	chr17	chr17	chr17	chr17	chr17	chr17
Strand	-	-	-	-	-	-	-	-
Start	83985915	83987076	83987106	83987225	83987401	84030310	84030673	84031101
Stop	83986992	83987105	83987194	83987290	83987710	84030335	84030795	84031181
P30.WT.CSMN	6.7	4.7	5.3	2.8	5.3	7.6	5.1	7
P30.SOD1.CSMN	8.6	6.6	6.1	7.2	7	9.3	7.7	8.4

Kcng4::TranscriptClusterID:6985813

Exon.ID	830432	830432	830432	830433	830434	830434	830435	830437
mRNA.Accession	NM_025734	NM_025734	NM_025734	NM_025734	NM_025734	NM_025734	NM_025734	NM_025734
Chromosome	chr8	chr8	chr8	chr8	chr8	chr8	chr8	chr8
Strand	-	-	-	-	-	-	-	-
Start	122147781	122147866	122148127	122149628	122156801	122157476	122157543	122159293
Stop	122147852	122147921	122149104	122150164	122157426	122157508	122157571	122159506
P30.WT.CSMN	2	6	5.9	6.2	7	4.8	6.2	4.4
P30.SOD1.CSMN	5.2	8	9.1	7.7	8.6	8	7.8	6.8

Kcnh1::TranscriptClusterID:6756419

Exon.ID	30910	30910	30914	30915	30918	30919	30923	30923	30937	30946	30947	30965	30965	30965	30966
mRNA.Accession	NM_010600	NM_010600	NM_010600	NM_010600	NM_010600	NM_010600	NM_010600	NM_010600	NM_010600	NM_010600	NM_010600	NM_010600	NM_010600	NM_010600	NM_010600
Chromosome	chr1	chr1	chr1	chr1	chr1	chr1	chr1	chr1	chr1	chr1	chr1	chr1	chr1	chr1	chr1
Strand	+	+	+	+	+	+	+	+	+	+	+	+	+	+	+
Start	194014543	194014917	194045230	194048764	194062621	194065807	194100581	194100973	194161303	194236913	194242581	194329483	194329945	194330271	194333008
Stop	194014828	194014967	194045320	194048827	194062734	194065910	194100945	194101005	194161698	194237061	194242735	194329927	194330002	194331421	194333688
P30.WT.CSMN	7.3	9.5	9.7	7.9	9.1	9.5	9.1	8.2	8.5	7.6	8.7	9.2	8.3	7.9	8.2
P30.SOD1.CSMN	8.4	9.7	10.4	8.6	9.9	10.3	10.4	9.2	10.1	9	9.5	10.4	9.4	9.2	9

Kcnh2::TranscriptClusterID:6936719

Exon.ID	657839	657840	657841	657841	657842	657843	657843	657844	657845	657846	657847	657848
mRNA.Accession	NM_013569	NM_013569	NM_013569	NM_013569	NM_013569	NM_013569	NM_013569	NM_013569	NM_013569	NM_013569	NM_013569	NM_013569
Chromosome	chr5	chr5	chr5	chr5	chr5	chr5	chr5	chr5	chr5	chr5	chr5	chr5
Strand	-	-	-	-	-	-	-	-	-	-	-	-
Start	23825536	23825861	23827063	23827173	23827463	23827764	23827872	23828475	23828890	23830167	23830797	23831370
Stop	23825807	23825964	23827117	23827239	23827587	23827812	23828005	23828531	23829057	23830347	23830887	23831589
P30.WT.CSMN	6.8	7.4	7.4	7.4	7	4.4	6.5	4.1	7.7	6.8	6.6	8.9
P30.SOD1.CSMN	7.1	9.4	9.1	7.9	8.3	6.2	8.9	6.1	8.8	8.4	7.7	10

Exon.ID	657849	657851	657851	657853	657853	657854	657855	657860	657861	657861
mRNA.Accession	NM_013569	ENSMUST00000115098	AF034762	NM_013569	NM_013569	NM_013569	NM_013569	NM_013569	NM_013569	NM_013569
Chromosome	chr5	chr5	chr5	chr5	chr5	chr5	chr5	chr5	chr5	chr5
Strand	-	-	-	-	-	-	-	-	-	-
Start	23832241	23834993	23835103	23836740	23836896	23837565	23838735	23854129	23857026	23857155
Stop	23832476	23835021	23835156	23836890	23836926	23837930	23838836	23854333	23857054	23857355
P30.WT.CSMN	7	6.7	5.1	8.8	8	6.3	8	7.4	8.1	5.4
P30.SOD1.CSMN	8.7	8.9	6.6	10.2	9	8.4	9.2	7.5	8.9	7.3

Kcnh3::TranscriptClusterID:6833153

Exon.ID	298136	298137	298138	298140	298141	298142	298143	298143	298145	298146	298147	298148	298149	298150	298150	298151
mRNA.Accession	NM_010601	NM_010601	NM_010601	NM_010601	NM_010601	NM_010601	NM_010601	NM_010601	NM_010601	NM_010601	NM_010601	NM_010601	NM_010601	NM_010601	NM_010601	AK135093
Chromosome	chr15	chr15	chr15	chr15	chr15	chr15	chr15	chr15	chr15	chr15	chr15	chr15	chr15	chr15	chr15	chr15
Strand	+	+	+	+	+	+	+	+	+	+	+	+	+	+	+	+
Start	99055417	99056786	99057421	99058351	99058939	99059495	99059737	99059809	99063151	99063271	99063726	99064318	99068537	99070148	99070258	99070863
Stop	99055445	99056966	99057527	99058430	99059055	99059639	99059783	99059943	99063198	99063316	99063873	99064522	99068698	99070221	99070316	99071080
P30.WT.CSMN	7.9	9.8	8.8	7.8	9.3	9.4	10.5	10.2	9.6	10.4	10.4	8.3	7.9	8.5	8.5	7.7
P30.SOD1.CSMN	9.9	10.6	10.3	9.2	10.3	10.1	11.6	11.1	10.4	11.8	11.5	9.4	9.4	10.2	10.4	7.9

Exon.ID	298152	298152	298153	298154	298154	298154	298154	298154	298154	298154	298155	298155
mRNA.Accession	NM_010601	NM_010601	AK135093	AK135093	NM_010601	AK135093	NM_010601	NM_010601	NM_010601	NM_010601	NM_010601	NM_010601
Chromosome	chr15	chr15	chr15	chr15	chr15	chr15	chr15	chr15	chr15	chr15	chr15	chr15
Strand	+	+	+	+	+	+	+	+	+	+	+	+
Start	99071354	99071380	99071467	99072150	99072228	99072307	99072388	99072670	99072704	99072847	99072900	99072961
Stop	99071378	99071404	99072053	99072178	99072257	99072332	99072549	99072696	99072842	99072877	99072933	99073202
P30.WT.CSMN	7.2	7.1	3.9	6.2	7.2	7.8	8.1	7.8	7.3	8	8	7.3
P30.SOD1.CSMN	8	8.1	5.9	6.2	8.3	9.2	9.7	8.1	7.5	8.8	9.3	9.3

Kcnh4::TranscriptClusterID:6791441

Exon.ID	156107	156107	156108	156111	156112	156113	156113	156114	156116	156117	156118	156119	156120	156120	156122	156123	156125	156125	156126	156127	156128	156128	156128
mRNA.Accession	NM_001081194	NM_001081194	NM_001081194	NM_001081194	NM_001081194	NM_001081194	NM_001081194	NM_001081194	NM_001081194	NM_001081194	NM_001081194	NM_001081194	NM_001081194	NM_001081194	NM_001081194	NM_001081194	NM_001081194	NM_001081194	NM_001081194	NM_001081194	NM_001081194	NM_001081194	NM_001081194
Chromosome	chr11	chr11	chr11	chr11	chr11	chr11	chr11	chr11	chr11	chr11	chr11	chr11	chr11	chr11	chr11	chr11	chr11	chr11	chr11	chr11	chr11	chr11	chr11
Strand	-	-	-	-	-	-	-	-	-	-	-	-	-	-	-	-	-	-	-	-	-	-	-
Start	100601718	100601911	100602624	100605438	100606582	100606933	100607139	100607487	100608117	100608894	100610943	100611576	100613559	100613727	100616482	100617051	100618200	100618274	100618393	100618941	100620871	100620924	100621056
Stop	100601827	100601935	100603264	100605463	100606656	100607125	100607281	100607515	100608294	100609104	100611044	100611720	100613670	100613753	100616597	100617223	100618272	100618300	100618492	100619111	100620911	100620952	100621228
P30.WT.CSMN	5.8	5.2	5	2.9	5.6	7.6	6.9	7.2	4.8	7	4.2	5.3	6.3	7.1	7.1	5.9	4.9	4.7	5.1	6	7.6	5.9	4.2
P30.SOD1.CSMN	6.2	8.4	6.7	6.8	6.4	8.4	8.3	8.6	6	7.8	5.8	7.5	8.4	8.5	8.2	7.8	7.1	6.9	6	7.3	9.3	7.5	5.3

Kcnh5::TranscriptClusterID:6801807

Exon.ID	192160	192161	192162	192163	192164	192165	192165	192165	192165	192165	192171	192172	192175	192187	192187	192187	192195	192199	192202	192204	192206	192210	192210
mRNA.Accession	AK034056	AK034056	AK083633	AK083633	NM_172805	NM_172805	NM_172805	NM_172805	NM_172805	NM_172805	NM_172805	NM_172805	NM_172805	NM_172805	NM_172805	NM_172805	NM_172805	NM_172805	NM_172805	NM_172805	NM_172805	NM_172805	NM_172805
Chromosome	chr12	chr12	chr12	chr12	chr12	chr12	chr12	chr12	chr12	chr12	chr12	chr12	chr12	chr12	chr12	chr12	chr12	chr12	chr12	chr12	chr12	chr12	chr12
Strand	-	-	-	-	-	-	-	-	-	-	-	-	-	-	-	-	-	-	-	-	-	-	-
Start	75988752	75990301	75995240	75996046	75998496	75998604	75998686	75998950	75999191	75999265	76066123	76077535	76108633	76188424	76188461	76188536	76215261	76220910	76231889	76238655	76255156	76277445	76277707
Stop	75989156	75990726	75995446	75996244	75998555	75998655	75998795	75999174	75999254	75999438	76066272	76077641	76108731	76188460	76188506	76188761	76215528	76221022	76232000	76238732	76255265	76277494	76278181
P30.WT.CSMN	5.6	6.4	6.2	8.6	6.2	7.3	6.2	8.6	7.8	5.4	8.2	7.7	7	8.1	9.2	8.6	8.2	8.3	7.3	8.3	6.4	7.8	8.2
P30.SOD1.CSMN	6.1	7.4	7.5	10.1	8	8.7	8	10.4	9.3	8	10.2	8.6	7.4	9.5	11	10.9	10.1	9.8	8.7	10	8.2	9.8	9.2

Kcnh6::TranscriptClusterID:6753646

Exon.ID	21732
mRNA.Accession	AK038513
Chromosome	chr1
Strand	+
Start	144485961
Stop	144485994
P30.WT.CSMN	4.1
P30.SOD1.CSMN	3.4

Kcnh6::TranscriptClusterID:6784588

Exon.ID	130743	130744	130746	130746	130747	130748	130749	130750	130751	130751	130753	130754
mRNA.Accession	NM_001037712	NM_001037712	NM_001037712	NM_001037712	NM_001037712	NM_001037712	NM_001037712	NM_001037712	NM_001037712	NM_001037712	NM_001037712	NM_001037712
Chromosome	chr11	chr11	chr11	chr11	chr11	chr11	chr11	chr11	chr11	chr11	chr11	chr11
Strand	+	+	+	+	+	+	+	+	+	+	+	+
Start	105869479	105870297	105875467	105875574	105875702	105878671	105880129	105881500	105881826	105881914	105885083	105887117
Stop	105869541	105870524	105875500	105875602	105875823	105878904	105880455	105881594	105881870	105882056	105885217	105887190
P30.WT.CSMN	6.6	3.7	6.3	6.7	6.2	5.5	4.1	7.3	10.4	7.7	8	6.9
P30.SOD1.CSMN	7.4	5.8	6.2	6.9	8.4	5.7	6.4	7.7	11.2	8.1	7.4	6.9

130758	130758	130759	130760	130761	130761	130761	130761	130761	130761
NM_001037712	NM_001037712	NM_001037712	ENSMUST00000051640	NM_001037712	NM_001037712	NM_001037712	ENSMUST00000001965	ENSMUST00000001965	ENSMUST00000001965
chr11	chr11	chr11	chr11	chr11	chr11	chr11	chr11	chr11	chr11
+	+	+	+	+	+	+	+	+	+
105888030	105888065	105888873	105894021	105895104	105895289	105895327	105895391	105895432	105895577
105888056	105888173	105889035	105894045	105895192	105895314	105895377	105895430	105895475	105895895
6.2	5.9	8	9.2	6.2	6.6	6.2	6.2	6.9	7.3
8.3	6	8.5	8.5	7	7.2	6.1	4.6	8.1	6.5

Kcnh6::TranscriptClusterID:6808750

Exon.ID	215173
mRNA.Accession	AK038513
Chromosome	chr13
Strand	+
Start	87492985
Stop	87493018
P30.WT.CSMN	3
P30.SOD1.CSMN	2.9

Kcnh6::TranscriptClusterID:6972654

Exon.ID	784648
mRNA.Accession	AK038513
Chromosome	chr7
Strand	+
Start	4411537
Stop	4411570
P30.WT.CSMN	3.1
P30.SOD1.CSMN	3

Kcnh6::TranscriptClusterID:6986860

Exon.ID	833813	833822	833823
mRNA.Accession	AK038513	BC044760	BC044760
Chromosome	chr9	chr9	chr9
Strand	+	+	+
Start	10197868	10290423	10294992
Stop	10197901	10290525	10295221
P30.WT.CSMN	2.8	8.4	8.5
P30.SOD1.CSMN	2.5	8.1	8

Kcnh7::TranscriptClusterID:6887205

Exon.ID	490261	490262	490263	490264	490265	490266	490267	490267	490267	490267
mRNA.Accession	ENSMUST00000075052	ENSMUST00000075052	ENSMUST00000075052	ENSMUST00000075052	ENSMUST00000075052	ENSMUST00000075052	ENSMUST00000075052	ENSMUST00000075052	ENSMUST00000075052	NM_133207
Chromosome	chr2	chr2	chr2	chr2	chr2	chr2	chr2	chr2	chr2	chr2
Strand	-	-	-	-	-	-	-	-	-	-
Start	62531585	62533057	62533463	62533935	62535733	62536708	62540470	62540626	62540761	62541003
Stop	62531686	62533123	62533596	62534485	62536249	62536974	62540564	62540704	62540996	62541058
P30.WT.CSMN	6.7	5.2	9.5	8.9	9	10.7	8.4	9.1	8.5	8.3
P30.SOD1.CSMN	7.8	5	10	9.6	9.1	11	8.8	9.7	9.5	9.3

Exon.ID	490267	490267	490268	490270	490271	490271	490272	490273	490274	490275	490276	490284	490287	490288	490288	490289	490293
mRNA.Accession	NM_133207	NM_133207	NM_133207	NM_133207	NM_133207	NM_133207	NM_133207	NM_133207	NM_133207	AK149160	AK149160	NM_133207	NM_133207	ENSMUST00000112454	NM_133207	NM_133207	ENSMUST00000104358
Chromosome	chr2	chr2	chr2	chr2	chr2	chr2	chr2	chr2	chr2	chr2	chr2	chr2	chr2	chr2	chr2	chr2	chr2
Strand	-	-	-	-	-	-	-	-	-	-	-	-	-	-	-	-	-
Start	62541003	62541123	62543988	62554207	62559777	62559840	62572286	62574129	62577237	62578787	62578909	62602640	62615462	62624527	62625684	62625955	62640581
Stop	62541058	62541268	62544158	62554298	62559802	62559992	62572355	62574210	62577381	62578852	62579148	62602816	62615706	62625282	62625856	62626054	62640665
P30.WT.CSMN	8.3	9.2	9.3	9	9.9	9.2	9.4	9.3	8.5	3.7	5.9	9.7	9.2	8	10.2	10	5.9
P30.SOD1.CSMN	9.3	9.7	9.4	9.1	10.3	9.5	9	9.7	9	4.9	6.8	10.3	10.2	8.1	10.8	10.2	5.9

Exon.ID	490298	490302	490305	490317	490318	490319	490320	490325	490329	490330	490338	490339	490343	490343	490343	490344	490344
mRNA.Accession	NM_133207	NM_133207	NM_133207	AK043796	AK043796	AK051001	AK051001	AK045846	AK039646	AK039646	AK035130	AK035130	AK043768	AK043768	NM_133207	NM_133207	NM_133207
Chromosome	chr2	chr2	chr2	chr2	chr2	chr2	chr2	chr2	chr2	chr2	chr2	chr2	chr2	chr2	chr2	chr2	chr2
Strand	-	-	-	-	-	-	-	-	-	-	-	-	-	-	-	-	-
Start	62675123	62688474	62715263	62805361	62806675	62834490	62835409	62945252	62962286	62963315	63003241	63005194	63019756	63020078	63020113	63022062	63022153
Stop	62675313	62688718	62715371	62805964	62807081	62835056	62835825	62945851	62962787	62963807	63003753	63005417	63020077	63020109	63020257	63022114	63022287
P30.WT.CSMN	10.3	11.3	10	8.5	7.8	8.3	7.9	8	9.6	9	8.8	8	7.2	6	10.9	11.1	8
P30.SOD1.CSMN	10.7	11.6	9.9	8.1	6.7	7.6	6.6	7.5	9.6	8.7	7.9	7.8	5.4	5.3	11.4	11	8.7

Kcnip1::TranscriptClusterID:6787197

Exon.ID	140221	140221	140221	140223	140225	140226	140226	140227	140227	140227	140227	140227	140227
mRNA.Accession	AY171234	NM_027398	NM_027398	NM_027398	NM_027398	AK048178	NM_027398	NM_027398	AK048178	NM_027398	AK048178	NM_027398	NM_027398
Chromosome	chr11	chr11	chr11	chr11	chr11	chr11	chr11	chr11	chr11	chr11	chr11	chr11	chr11
Strand	-	-	-	-	-	-	-	-	-	-	-	-	-
Start	33529412	33529825	33530560	33533162	33534615	33541784	33542433	33543307	33543656	33544504	33544622	33545508	33545599
Stop	33529717	33530503	33530586	33533197	33534648	33542285	33542503	33543364	33544342	33544563	33545115	33545569	33545627
P30.WT.CSMN	3.6	6.3	2.7	4.2	5.9	4.1	3.6	7.3	6.1	5.7	5.4	7.7	6.3
P30.SOD1.CSMN	5.3	8	6.5	5.7	7.9	5.6	6.4	9.6	6.9	7.4	6.3	8.5	8.3

Exon.ID	140227	140229	140270	140270	140270	140270	140270	140270	140277
mRNA.Accession	AY171234	ENSMUST00000065970	AK013657	AK013657	AK013657	NM_027398	NM_027398	NM_027398	DQ148494
Chromosome	chr11	chr11	chr11	chr11	chr11	chr11	chr11	chr11	chr11
Strand	-	-	-	-	-	-	-	-	-
Start	33545642	33551541	33742379	33742990	33743044	33743133	33743191	33743249	33892913
Stop	33545753	33551572	33742821	33743034	33743121	33743190	33743227	33743698	33893164
P30.WT.CSMN	5.7	3.4	5.9	3.7	6.2	7.1	6.3	5.3	5.2
P30.SOD1.CSMN	7.1	5.6	6.7	5.4	7.1	9	8.6	7.1	7

Kcnip2::TranscriptClusterID:6873368

Exon.ID	440536	440536	440536	440536	440537	440538	440539	440540	440541	440543
mRNA.Accession	ENSMUST00000079431	ENSMUST00000079431	ENSMUST00000079431	NM_145703	NM_145703	NM_145703	NM_145703	NM_145703	NM_145703	NM_145703
Chromosome	chr19	chr19	chr19	chr19	chr19	chr19	chr19	chr19	chr19	chr19
Strand	-	-	-	-	-	-	-	-	-	-
Start	45866882	45867467	45867691	45868165	45868493	45868728	45868976	45869242	45869481	45870082
Stop	45867443	45867509	45868041	45868207	45868541	45868793	45869063	45869295	45869536	45870181
P30.WT.CSMN	7.6	6.3	6.7	6.3	6	7	7	7	7.8	6.2
P30.SOD1.CSMN	10	8.9	8.9	8.6	8	9.2	10	9	9.7	8.4

Exon.ID	440544	440544	440545	440550	440552	440554	440554
mRNA.Accession	NM_145703	BC056434	NM_145703	AK137312	ENSMUST00000086993	NM_145703	ENSMUST00000079431
Chromosome	chr19	chr19	chr19	chr19	chr19	chr19	chr19
Strand	-	-	-	-	-	-	-
Start	45870769	45870833	45871601	45881457	45886697	45890231	45890341
Stop	45870794	45871429	45871641	45881642	45886735	45890286	45890624
P30.WT.CSMN	7.8	6.9	8.3	7	4.3	5.7	6
P30.SOD1.CSMN	9.5	6.5	10.5	8.8	5.5	7.5	8.5

Kcnip3::TranscriptClusterID:6890699

Exon.ID	502743	502743	502744	502745	502746	502750	502751	502752	502761	502762	502780	502784	502785
mRNA.Accession	NM_019789	NM_019789	NM_019789	NM_019789	NM_019789	NM_019789	NM_019789	NM_019789	NM_001111331	NM_001111331	NM_019789	NM_019789	AK016721
Chromosome	chr2	chr2	chr2	chr2	chr2	chr2	chr2	chr2	chr2	chr2	chr2	chr2	chr2
Strand	-	-	-	-	-	-	-	-	-	-	-	-	-
Start	127282536	127284077	127285077	127285749	127286178	127290792	127291101	127291596	127307744	127308013	127336588	127346993	127347531
Stop	127283774	127284124	127285117	127285797	127286276	127290829	127291139	127291697	127307836	127308135	127336695	127347097	127347814
P30.WT.CSMN	8.6	12.1	12	11.9	12	12.4	10.3	11.4	6.9	7.5	10.9	9.5	6
P30.SOD1.CSMN	9.9	12.3	12	11.9	11.8	12.4	9.4	11	7.8	7.9	10.7	9.4	7.9

Kcnip4::TranscriptClusterID:6937986

Exon.ID	662577	662577	662577	662580	662581	662583	662587	662590	662597	662599	662603	662606	662607	662620	662622	662623	662626	662627	662636
mRNA.Accession	NM_030265	NM_030265	NM_030265	NM_030265	NM_030265	NM_030265	NM_030265	NM_030265	NM_030265	AY647240	BC051130	AK045382	AK045382	AK049021	DQ148504	AK049021	AK158542	AK158542	AK141008
Chromosome	chr5	chr5	chr5	chr5	chr5	chr5	chr5	chr5	chr5	chr5	chr5	chr5	chr5	chr5	chr5	chr5	chr5	chr5	chr5
Strand	-	-	-	-	-	-	-	-	-	-	-	-	-	-	-	-	-	-	-
Start	48780806	48782053	48782165	48787245	48787893	48789718	48801040	48810460	48873803	48883408	48900934	48926687	48928812	48982205	48990663	48990998	49016106	49018053	4910364[illegible]
Stop	48781974	48782114	48782195	48787286	48787980	48789799	48801090	48810520	48873872	48883483	48900997	48927130	48928996	48982565	48990877	48991181	49016288	49018277	4910387[illegible]
P30.WT.CSMN	10	9.9	9.9	10.2	10.6	8.7	10.9	8	7.3	5.9	7.5	4.1	6.6	7.5	7	6.9	5.8	6.1	9.4
P30.SOD1.CSMN	10.7	10.3	10.4	10.2	10.8	8.2	11.4	8.3	8.7	7.4	7.3	4.2	6.4	7.3	7.3	7.3	5.3	6.4	8.4

Exon.ID	662637	662644	662649	662650	662651	662652	662656	662657	662666	662666	662671	662673	662674	662675	662676	662677	662678
mRNA.Accession	AK141008	AK133416	AK038678	AK038678	AK138340	AK138340	AK039632	AK039632	NM_030265	NM_030265	AK148828	AK138288	AK138288	AK021085	AK089840	AK083109	AK043572
Chromosome	chr5	chr5	chr5	chr5	chr5	chr5	chr5	chr5	chr5	chr5	chr5	chr5	chr5	chr5	chr5	chr5	chr5
Strand	-	-	-	-	-	-	-	-	-	-	-	-	-	-	-	-	-
Start	49106510	49145699	49190450	49192012	49194004	49194324	49234818	49237179	49280609	49280705	49339377	49369548	49370442	49389245	49396744	49409053	4941328[illegible]
Stop	49106905	49145738	49190825	49192431	49194165	49195149	49235159	49237554	49280699	49280786	49339685	49369771	49370593	49389736	49397248	49409709	4941346[illegible]
P30.WT.CSMN	7.8	6	7.3	7.6	8.6	8.7	7.1	7.9	6.6	5	8.5	7.5	8.7	6.8	7.9	8.3	9.4
P30.SOD1.CSMN	7.5	7.3	6.6	8.6	8.3	8.3	6.7	5.9	7.3	5.3	8.7	7.9	8.6	5.2	7.6	8.5	10.1

Exon.ID	662680	662684	662686	662687	662701	662703	662704	662705	662713	662722	662723	662741	662742	662746
mRNA.Accession	AK149080	AK149261	AK036025	AK036025	AK080530	AK144382	AK144382	AK144382	AK148685	AK035988	AK035988	AK090283	AK090283	BC051130
Chromosome	chr5	chr5	chr5	chr5	chr5	chr5	chr5	chr5	chr5	chr5	chr5	chr5	chr5	chr5
Strand	-	-	-	-	-	-	-	-	-	-	-	-	-	-
Start	49462104	49507064	49515941	49519402	49608553	49610933	49611667	49612665	49676511	49800069	49802572	49907124	49908172	49915904
Stop	49462567	49507144	49516316	49519901	49608891	49611249	49612123	49613190	49676898	49800463	49802952	49907281	49908369	49916031
P30.WT.CSMN	7.9	8.1	8.5	8.5	8.9	8.7	9.4	8.1	7.8	6.5	7.1	6.7	9.4	8.9
P30.SOD1.CSMN	7.6	7.8	8.3	9	8.4	8.3	8.2	8.2	7.4	6.4	6.2	6.1	7.6	9.1

Kcnq1::TranscriptClusterID:6965319

Exon.ID	758555	758555	758555	758564	758566	758572	758573	758574	758575	758582	758583	758584	758585	758591	758603
mRNA.Accession	NM_008434	NM_008434	NM_008434	ENSMUST00000105918	NM_008434	NM_008434	NM_008434	NM_008434	NM_008434	NM_008434	NM_008434	NM_008434	NM_008434	NM_008434	AK052149
Chromosome	chr7	chr7	chr7	chr7	chr7	chr7	chr7	chr7	chr7	chr7	chr7	chr7	chr7	chr7	chr7
Strand	+	+	+	+	+	+	+	+	+	+	+	+	+	+	+
Start	150293172	150293281	150293521	150334670	150335408	150368638	150369003	150369552	150370317	150377341	150378493	150380139	150381182	150447181	15048377[illegible]
Stop	150293247	150293434	150293646	150334694	150335480	150368757	150369074	150369590	150370437	150377424	150378559	150380219	150381308	150447261	15048392[illegible]
P30.WT.CSMN	7	5.3	5.1	5.7	6	6.5	3.8	4.4	2.9	3.5	5.9	4.2	4.1	6.8	4.9
P30.SOD1.CSMN	9.1	7.1	6.4	4.2	6.8	7.7	6.9	6.9	5.4	5.2	7.4	7.1	3.8	7.9	6.8

Exon.ID	758604	758605	758605	758617	758618	758619	758620	758621	758642	758642
mRNA.Accession	AK052149	AK052149	AK052149	AK045069	NM_008434	NM_008434	NM_008434	NM_008434	NM_008434	NM_008434
Chromosome	chr7	chr7	chr7	chr7	chr7	chr7	chr7	chr7	chr7	chr7
Strand	+	+	+	+	+	+	+	+	+	+
Start	150484326	150486928	150487290	150531140	150544617	150548501	150549135	150549539	150611880	150612472
Stop	150484376	150487217	150487481	150531712	150544679	150548577	150549180	150549589	150611999	150612848
P30.WT.CSMN	4.6	3.5	4	3.6	3.5	3.7	5	6.3	4.6	5
P30.SOD1.CSMN	5.9	3.3	5	6	6.2	6	6.3	6.8	6.3	6.3

Kcnq2::TranscriptClusterID:6894265

Exon.ID	514515	514516	514517	514517	514517	514517	514517	514517	514517	514517	514517	514517	514517
mRNA.Accession	NM_001003824	NM_001003824	ENSMUST00000103051	ENSMUST00000103051	ENSMUST00000103051	ENSMUST00000103051	ENSMUST00000103051	ENSMUST00000103051	ENSMUST00000103051	NM_010611	NM_010611	NM_010611	NM_010611
Chromosome	chr2	chr2	chr2	chr2	chr2	chr2	chr2	chr2	chr2	chr2	chr2	chr2	chr2
Strand	-	-	-	-	-	-	-	-	-	-	-	-	-
Start	180810330	180810878	180812312	180814688	180814854	180814933	180814999	180815088	180815311	180815758	180815811	180815943	180816270
Stop	180810632	180810907	180814352	180814810	180814883	180814983	180815053	180815120	180815511	180815795	180815889	180816036	180816457
P30.WT.CSMN	8	9.4	10.9	9.1	9.1	8.1	8.7	9.4	9.8	9.5	8.7	8.1	9.7
P30.SOD1.CSMN	9.1	10.3	11.5	8.5	10	8.4	9.2	9.7	10.5	10.3	9.5	8.6	10.4

514518	514519	514519	514519	514519	514519	514520	514521	514522	514523	514526	514527	514527	514527	514529	514529	514530
NM_010611	AK079328	NM_001003824	NM_001006675	NM_001006675	NM_001003825	NM_010611	NM_010611	NM_010611	NM_010611	NM_010611	NM_001006677	NM_001006676	NM_010611	NM_010611	AK079328	NM_010611N
chr2	chr2	chr2	chr2	chr2	chr2	chr2	chr2	chr2	chr2	chr2	chr2	chr2	chr2	chr2	chr2	chr2
-	-	-	-	-	-	-	-	-	-	-	-	-	-	-	-	-
180817076	180819575	180819955	180820114	180821488	180821582	180821680	180822280	180823052	180823240	180826947	180831091	180831511	180831655	180835261	180835335	1808383501
180817136	180819902	180820055	180821105	180821563	180821646	180821774	180822354	180823178	180823272	180826997	180831443	180831597	180831684	180835288	180835450	1808383761
9.4	7.2	7.4	8.1	7.5	7.9	10.2	9.5	9.9	8.8	10	7.6	6.4	11	9.7	8.1	10.8
10.1	7.4	7.5	7.5	7.6	8.6	11	10.4	10.4	9.3	10.5	7	6.1	11.7	10	8.5	10.6

514531	514534	514534	514534	514535	514536	514536	514536	514536	514537	514538	514539	514540	514549	514549
1NM_010611	NM_001006678	NM_001006678	NM_001006679	NM_010611	NM_001006680	NM_001006680	NM_001006680	NM_010611	NM_010611	NM_010611	NM_010611	NM_010611	NM_010611	NM_010611
chr2	chr2	chr2	chr2	chr2	chr2	chr2	chr2	chr2	chr2	chr2	chr2	chr2	chr2	chr2
-	-	-	-	-	-	-	-	-	-	-	-	-	-	-
0180839603	180843175	180843240	180843272	180843728	180843922	180844304	180844350	180844391	180846722	180847779	180848326	180849344	180869505	180869804
6180839676	180843203	180843266	180843520	180843814	180844204	180844337	180844379	180844474	180846825	180847933	180848403	180849394	180869653	180869926
10.6	8.1	9.9	9.3	11.6	6.2	5.6	7.3	11.4	11.5	11.8	11	10.2	8.9	7.6
11.1	7	10.7	9.1	11.7	6	7	8.5	11.5	11.7	12	11.3	9.4	8.6	8.8

Kcnq3::TranscriptClusterID:6836305

Exon.ID	308693	308694	308694	308695	308698	308700	308701	308702	308705	308707	308707	308708	308709	308710	308711	308712
mRNA.Accession	AK046858	NM_152923	NM_152923	NM_152923	NM_152923	NM_152923	NM_152923	NM_152923	NM_152923	NM_152923	NM_152923	NM_152923	NM_152923	NM_152923	NM_152923	NM_152923N
Chromosome	chr15	chr15	chr15	chr15	chr15	chr15	chr15	chr15	chr15	chr15	chr15	chr15	chr15	chr15	chr15	chr15
Strand	-	-	-	-	-	-	-	-	-	-	-	-	-	-	-	-
Start	65818172	65826736	65827124	65829281	65831626	65833763	65836289	65837352	65848011	65851870	65851909	65853303	65855243	65856791	65860239	65862102
Stop	65820907	65826768	65827444	65829314	65831677	65833850	65836355	65837522	65848037	65851905	65851941	65853398	65855310	65856826	65860357	65862179
P30.WT.CSMN	8.4	8.3	9.1	9.4	7.5	9.6	7.8	9.8	10.5	8	7.7	8.8	9.3	9.6	8.8	9.8
P30.SOD1.CSMN	9.6	9.1	9.5	10.1	7.9	10.3	8	10.2	10.5	7.4	8	9.7	9.9	10.5	9.6	10.7

308713	308735	308741	308742	308743	308744	308744	308745	308754	308755	308756	308756	308757	308757	308757
3NM_152923	AK158928	AK088992	AK088992	AK158948	AK158948	AK158948	AK158948	AK043539	AK043539	AK043539	AK043539	AK043539	NM_152923	NM_152923
chr15	chr15	chr15	chr15	chr15	chr15	chr15	chr15	chr15	chr15	chr15	chr15	chr15	chr15	chr15
-	-	-	-	-	-	-	-	-	-	-	-	-	-	-
65862980	66067513	66091780	66092815	66098379	66099592	66099737	66100119	66114861	66115596	66115949	66116025	66117018	66117435	66117693
65863016	66067697	66092355	66093330	66098671	66099720	66099765	66100307	66115133	66115708	66116010	66116151	66117321	66117637	66117719
10.1	8.1	8.8	8.6	8.8	10.8	10.5	9	9.3	7.9	7.2	8	7.8	8	8.2
10.9	7.8	8.9	8.1	7.9	9.4	9	6.5	8.2	7	6.5	6.7	7.8	8.1	9.8

Kcnq4::TranscriptClusterID:6925062

Exon.ID	615556	615557	615558	615559	615561	615566	615566	615568	615569	615570
mRNA.Accession	NM_001081142	NM_001081142	NM_001081142	NM_001081142	NM_001081142	NM_001081142	NM_001081142	NM_001081142	NM_001081142	NM_001081142
Chromosome	chr4	chr4	chr4	chr4	chr4	chr4	chr4	chr4	chr4	chr4
Strand	-	-	-	-	-	-	-	-	-	-
Start	120369037	120371122	120372511	120374991	120376965	120383847	120383946	120385626	120387607	120387876
Stop	120370426	120371186	120372590	120375045	120377180	120383905	120383982	120385699	120387632	120387947
P30.WT.CSMN	4.3	4.1	3.3	5.3	3.2	7.8	4.9	6	5.4	5.1
P30.SOD1.CSMN	6.8	7.5	6.5	6.7	6.3	9.2	6.2	7	7.2	7.2

Exon.ID	615571	615573	615574	615575	615585	615585	615585	615587
mRNA.Accession	NM_001081142	NM_001081142	NM_001081142	NM_001081142	NM_001081142	NM_001081142	NM_001081142	AK163659
Chromosome	chr4	chr4	chr4	chr4	chr4	chr4	chr4	chr4
Strand	-	-	-	-	-	-	-	-
Start	120388379	120389159	120389546	120390063	120419465	120419548	120419640	120421150
Stop	120388494	120389300	120389654	120390134	120419543	120419618	120419709	120421191
P30.WT.CSMN	5.2	1.9	3.8	5.6	5.9	8.6	3.6	3.4
P30.SOD1.CSMN	7.1	6.2	7.8	7	7.8	10.1	7.2	5.1

Kcnq5::TranscriptClusterID:6757326

Exon.ID	34047
mRNA.Accession	NM_023872
Chromosome	chr1
Strand	-
Start	21389160
Stop	21389809
P30.WT.CSMN	8
P30.SOD1.CSMN	8.7

Kcnq5::TranscriptClusterID:6757327

Exon.ID	34048
mRNA.Accession	NM_023872
Chromosome	chr1
Strand	-
Start	21390347
Stop	21390744
P30.WT.CSMN	10.1
P30.SOD1.CSMN	11

Kcnq5::TranscriptClusterID:6757328

Exon.ID	34049
mRNA.Accession	NM_023872
Chromosome	chr1
Strand	-
Start	21390863
Stop	21391056
P30.WT.CSMN	9.7
P30.SOD1.CSMN	10.5

Kcnq5::TranscriptClusterID:6757329

Exon.ID	34050	34050	34051	34052	34055	34057	34058	34059	34059	34059	34059	34060	34060	34064	34064	34065	34068
mRNA.Accession	NM_023872	NM_023872	NM_023872	NM_023872	NM_023872	AK132615	AK132615	AK132615	AK132615	AK132615	AK132615	NM_023872	NM_023872	ENSMUST00000115299	NM_023872	NM_023872	NM_023872
Chromosome	chr1	chr1	chr1	chr1	chr1	chr1	chr1	chr1	chr1	chr1	chr1	chr1	chr1	chr1	chr1	chr1	chr1
Strand	-	-	-	-	-	-	-	-	-	-	-	-	-	-	-	-	-
Start	21391712	21392265	21395017	21395914	21414130	21444850	21446140	21446471	21446528	21446591	21446735	21447509	21447675	21456342	21456464	21459584	21469510
Stop	21392185	21393184	21395110	21395995	21414238	21445364	21446175	21446522	21446589	21446640	21447047	21447636	21447701	21456450	21456490	21459645	21469537
P30.WT.CSMN	9.7	8.4	9.9	8.3	8.8	4.5	8.2	6.6	4.1	5.7	7.1	8.6	7	7.6	9.2	9.8	7.9
P30.SOD1.CSMN	10.3	9	10.4	9	9	5.1	8.5	7.3	6.1	6.1	7.6	9.5	7.7	6.5	9.8	10.6	7.7

34070	34071	34072	34072	34077	34080	34097	34098	34105	34106	34110	34111	34113	34114	34115	34119	34120	34126
NM_023872	NM_023872	NM_023872	NM_023872	NM_023872	NM_023872	AK042152	AK042152	AK083993	AK083993	AK049918	AK049918	AK037950	AK037950	AK037950	AK040086	AK040086	AK080550
chr1	chr1	chr1	chr1	chr1	chr1	chr1	chr1	chr1	chr1	chr1	chr1	chr1	chr1	chr1	chr1	chr1	chr1
-	-	-	-	-	-	-	-	-	-	-	-	-	-	-	-	-	-
21477323	21495388	21495767	21495837	21525148	21583605	21794255	21795499	21817135	21818488	21829152	21830063	21835192	21835955	21836747	21847195	21851192	21887974
21477380	21495499	21495796	21495932	21525245	21583679	21794744	21795871	21817678	21818831	21829528	21830274	21835539	21836352	21837322	21847636	21851362	2188046
10.2	9.4	9.5	9.6	10.1	9.8	6.7	6.9	7.7	9.1	8.5	7.4	7.3	8.3	6.6	6.8	6.9	8.9
10.3	10.2	10.8	10.8	11.3	10.2	7.1	6.2	7.6	8.9	7.9	5.4	5.5	8.4	5.5	6	5.8	9.2

34127	34128	34131	34132	34133	34133	34133	34133	34133	34133	34134	34135	34135	34135
AK088791	AK088791	AK042483	AK039046	AK039046	NM_023872	NM_023872	NM_023872	NM_023872	NM_023872	NM_023872	AK039046	AK039046	AK039046
chr1	chr1	chr1	chr1	chr1	chr1	chr1	chr1	chr1	chr1	chr1	chr1	chr1	chr1
-	-	-	-	-	-	-	-	-	-	-	-	-	-
21895167	21897370	21941647	21949957	21951220	21951264	21951367	21951465	21951632	21951718	21951901	21952152	21952389	21952449
21895420	21897962	21941955	21950391	21951260	21951365	21951432	21951555	21951660	21951873	21952042	21952367	21952421	21952654
8.6	7	6.7	6.9	7	9.5	8.9	7.5	7.3	7.6	5.8	5.7	6.7	4.8
7.1	5.4	6.5	7.6	9	10.2	10.1	9.2	8.7	8.7	7.5	7	8.6	5.6

Kcns1::TranscriptClusterID:6892888

Exon.ID	510225	510225	510225	510226	510226	510227	510228
mRNA.Accession	NM_008435	NM_008435	AK140377	NM_008435	NM_008435	NM_008435	NM_008435
Chromosome	chr2	chr2	chr2	chr2	chr2	chr2	chr2
Strand	-	-	-	-	-	-	-
Start	163989525	163990288	163990804	163993707	163994531	163994846	163996774
Stop	163990184	163990731	163991196	163994375	163994573	163994977	163996862
P30.WT.CSMN	5.7	8.4	6	8	7.8	4.1	5.1
P30.SOD1.CSMN	7.4	9	4.8	8.9	9.5	6.1	7.3

Kcns2::TranscriptClusterID:6829687

Exon.ID	286273	286274	286275	286276	286276
mRNA.Accession	NM_181317	BC059833	NM_181317	NM_181317	NM_181317
Chromosome	chr15	chr15	chr15	chr15	chr15
Strand	+	+	+	+	+
Start	34767120	34767853	34768205	34768443	34770434
Stop	34767555	34767929	34768246	34769261	34772863
P30.WT.CSMN	5.5	7.3	8.2	7.3	6.8
P30.SOD1.CSMN	6.9	8.3	9.1	8.1	7.3

Kcns3::TranscriptClusterID:6798933

Exon.ID	183540	183540	183540	183540	183541	183544
mRNA.Accession	NM_173417	NM_173417	NM_173417	NM_173417	NM_173417	NM_173417
Chromosome	chr12	chr12	chr12	chr12	chr12	chr12
Strand	-	-	-	-	-	-
Start	11097387	11098303	11099503	11099534	11126574	11157004
Stop	11098026	11099389	11099529	11099561	11126737	11157119
P30.WT.CSMN	2.9	5.4	7.1	7.2	5.2	8.2
P30.SOD1.CSMN	5.8	6.4	8.6	8.7	6.9	9.9

Kcnv1::TranscriptClusterID:6835454

Exon.ID	305490	305490	305491	305491	305492	305492	305493
mRNA.Accession	NM_026200	NM_026200	AK038450	NM_026200	NM_026200	NM_026200	NM_026200
Chromosome	chr15	chr15	chr15	chr15	chr15	chr15	chr15
Strand	-	-	-	-	-	-	-
Start	44938154	44940595	44944332	44944632	44945839	44946188	44946346
Stop	44940467	44941029	44944435	44944851	44946057	44946255	44946460
P30.WT.CSMN	7.6	7.5	6.1	8	7.2	7.3	7.1
P30.SOD1.CSMN	9.3	9.5	5.9	9.5	8.3	8.9	9.2

Kcnv2::TranscriptClusterID:6868902

Exon.ID	423666	423666	423667	423668	423668	423668
mRNA.Accession	ENSMUST00000056708	NM_183179	NM_183179	NM_183179	NM_183179	NM_183179
Chromosome	chr19	chr19	chr19	chr19	chr19	chr19
Strand	+	+	+	+	+	+
Start	27397044	27397147	27397791	27408153	27408353	27409068
Stop	27397112	27397209	27398592	27408312	27408397	27411370
P30.WT.CSMN	4.5	5.3	3.9	4.2	4.8	3.3
P30.SOD1.CSMN	5.2	6.7	7.3	4.9	4.8	5.5

Kctd1::TranscriptClusterID:6863582

Exon.ID	405248	405248	405251	405256	405256	405256	405256	405256	405256	405256	405259	405264
mRNA.Accession	NM_001142731	NM_001142731	NM_001142731	AK054557	AK054557	AK054557	AK054557	AK054557	NM_001142731	NM_001142731	AK016623	NM_001142731
Chromosome	chr18	chr18	chr18	chr18	chr18	chr18	chr18	chr18	chr18	chr18	chr18	chr18
Strand	-	-	-	-	-	-	-	-	-	-	-	-
Start	15127390	15128065	15132536	15141286	15141935	15142200	15143181	15143303	15144856	15144914	15147437	15166250
Stop	15127980	15128149	15132656	15141928	15142094	15142712	15143243	15144790	15144894	15144962	15147739	15166366
P30.WT.CSMN	6.8	8.1	10.4	6.2	6.5	6.3	7.9	7.4	8.6	7	7.3	8.8
P30.SOD1.CSMN	7.1	8.8	11.9	6.2	6.8	6.3	8.2	7.4	9.4	7.7	7.8	9.9

Exon.ID	405281	405281	405281	405281	405281	405287	405287	405287	405288	405289	405297
mRNA.Accession	NM_001142731	NM_001142731	NM_001142731	NM_001142731	NM_001142731	AK140481	AK140481	AK140481	AK140481	AK140481	NM_134112
Chromosome	chr18	chr18	chr18	chr18	chr18	chr18	chr18	chr18	chr18	chr18	chr18
Strand	-	-	-	-	-	-	-	-	-	-	-
Start	15220292	15220432	15220544	15221606	15221665	15225406	15225504	15225635	15226812	15228466	15309895
Stop	15220413	15220463	15221177	15221633	15221740	15225437	15225618	15225905	15226887	15228545	15309934
P30.WT.CSMN	9.2	8.9	8.3	8	6.2	6	4.5	3.2	5.5	4.5	8.3
P30.SOD1.CSMN	10.5	10.4	9.4	9.5	7.7	7.7	5.6	4.7	5.4	4.8	9.9

Kctd2::TranscriptClusterID:6785133

Exon.ID	132645	132646	132647	132647	132647	132650	132651	132653	132655	132656	132656	132656	132656	132657
mRNA.Accession	ENSMUST00000103035	NM_183285	NM_183285	NM_183285	NM_183285	NM_183285	NM_183285	NM_183285	NM_183285	ENSMUST00000106533	ENSMUST00000106533	ENSMUST00000106533	ENSMUST00000106533	NM_183285
Chromosome	chr11	chr11	chr11	chr11	chr11	chr11	chr11	chr11	chr11	chr11	chr11	chr11	chr11	chr11
Strand	+	+	+	+	+	+	+	+	+	+	+	+	+	+
Start	115281440	115281498	115281578	115281742	115281796	115283299	115285812	115288765	115290601	115290781	115290990	115291133	115291241	115291714
Stop	115281481	115281525	115281741	115281793	115281820	115283398	115285869	115288836	115290716	115290869	115291069	115291162	115291432	115292359
P30.WT.CSMN	9.1	10	6.8	8.7	6.7	6.7	8.5	7.3	6.4	5.8	6.6	6.8	5.1	6.9
P30.SOD1.CSMN	10.6	11.4	8	9.7	8.7	7.4	8.8	8.6	7.5	6.7	7.6	6	6.8	8.1

Kctd3::TranscriptClusterID:6765119

Exon.ID	61853	61853	61854	61854	61855	61856	61857	61857	61858	61859	61859	61859	61860
mRNA.Accession	NM_172650	NM_172650	NM_172650	NM_172650	NM_172650	NM_172650	BC061458	NM_172650	NM_172650	AK033491	NM_172650	NM_172650	NM_172650
Chromosome	chr1	chr1	chr1	chr1	chr1	chr1	chr1	chr1	chr1	chr1	chr1	chr1	chr1
Strand	-	-	-	-	-	-	-	-	-	-	-	-	-
Start	190795006	190795602	190796230	190796368	190797957	190798257	190800250	190800557	190802462	190804899	190805124	190805232	190806890
Stop	190795035	190795791	190796302	190796523	190798044	190798336	190800407	190800586	190802487	190805004	190805173	190805274	190807004
P30.WT.CSMN	4	6.9	8.1	7	8.6	6	6.5	9.9	8.4	6	8	6.6	7.8
P30.SOD1.CSMN	5.4	7.6	9.5	7.8	9.9	7.2	6	11	8.9	6.1	8.4	6.9	8.3

Exon.ID	61861	61864	61864	61868	61869	61869	61869	61869	61873	61879	61879
mRNA.Accession	NM_172650	NM_172650	NM_172650	NM_172650	AK087407	NM_172650	AK087407	NM_172650	NM_172650	NM_172650	NM_172650
Chromosome	chr1	chr1	chr1	chr1	chr1	chr1	chr1	chr1	chr1	chr1	chr1
Strand	-	-	-	-	-	-	-	-	-	-	-
Start	190807089	190811396	190811485	190816497	190819050	190819626	190819768	190820299	190824134	190831425	190831549
Stop	190807169	190811461	190811510	190816587	190819434	190819690	190820160	190820419	190824204	190831471	190831647
P30.WT.CSMN	8	7.9	8.4	8.7	4.9	8.4	6.6	7.3	9.3	7.8	8.5
P30.SOD1.CSMN	8.8	9	10	9.8	5.3	9.1	7	8	10.6	9	9.6

Kctd4::TranscriptClusterID:6820361

Exon.ID	255278	255280	255280	255280	255280
mRNA.Accession	NM_026214	NM_026214	NM_026214	NM_026214	NM_026214
Chromosome	chr14	chr14	chr14	chr14	chr14
Strand	+	+	+	+	+
Start	76354851	76362207	76362504	76363279	76363931
Stop	76354994	76362300	76363145	76363642	76365115
P30.WT.CSMN	4.6	3.4	6.3	7.9	6.6
P30.SOD1.CSMN	6.1	6.4	6.3	7.5	6.4

Kctd4::TranscriptClusterID:6820362

Exon.ID	255281
mRNA.Accession	AK163645
Chromosome	chr14
Strand	+
Start	76365678
Stop	76365797
P30.WT.CSMN	6.5
P30.SOD1.CSMN	6.6

Kctd5::TranscriptClusterID:6854314

Exon.ID	372456	372456	372458	372460	372461	372462	372465	372465
mRNA.Accession	NM_027008	NM_027008	NM_027008	NM_027008	NM_027008	NM_027008	NM_027008	NM_027008
Chromosome	chr17	chr17	chr17	chr17	chr17	chr17	chr17	chr17
Strand	-	-	-	-	-	-	-	-
Start	24185262	24186382	24192896	24194714	24196191	24197213	24210163	24210400
Stop	24186265	24186411	24192946	24194803	24196267	24197311	24210395	24210439
P30.WT.CSMN	6.4	7.9	6.8	6.6	6	7	7.8	8.1
P30.SOD1.CSMN	7.3	8.5	8.2	7.8	5.1	7.3	8.7	9.6

Kctd6::TranscriptClusterID:6816720

Exon.ID	242763	242768	242768	242770	242770
mRNA.Accession	NM_027782	NM_027782	NM_027782	NM_027782	NM_027782
Chromosome	chr14	chr14	chr14	chr14	chr14
Strand	+	+	+	+	+
Start	9046645	9052678	9052708	9054841	9055419
Stop	9046849	9052707	9052734	9055373	9055859
P30.WT.CSMN	7.9	7.4	8.7	7.8	8.7
P30.SOD1.CSMN	9.1	8.2	9.2	8.3	9.4

Kctd6::TranscriptClusterID:6914384

Exon.ID	578788	578789
mRNA.Accession	NM_027782	ENSMUST00000119774
Chromosome	chr4	chr4
Strand	+	+
Start	70733707	70734105
Stop	70733836	70734138
P30.WT.CSMN	8.2	3.2
P30.SOD1.CSMN	8.9	3.5

Kctd7::TranscriptClusterID:6983955

Exon.ID	824049
mRNA.Accession	NM_172509
Chromosome	chr8
Strand	-
Start	88377350
Stop	88377376
P30.WT.CSMN	5
P30.SOD1.CSMN	6.9

Kctd7::TranscriptClusterID:6934657

Exon.ID	650367	650367	650368	650369	650370	650370	650371	650372	650372	650372
mRNA.Accession	NM_172509	NM_172509	NM_172509	NM_172509	NM_172509	NM_172509	NM_172509	NM_172509	NM_172509	NM_172509
Chromosome	chr5	chr5	chr5	chr5	chr5	chr5	chr5	chr5	chr5	chr5
Strand	+	+	+	+	+	+	+	+	+	+
Start	130620751	130620852	130620994	130623939	130627604	130627690	130628122	130628493	130628740	130629225
Stop	130620828	130620907	130621046	130624078	130627633	130627718	130628343	130628688	130628797	130631152
P30.WT.CSMN	6.2	7	7.1	7.3	4	5	6.1	7.2	5.5	5.7
P30.SOD1.CSMN	6.7	7.4	8	7.9	5.4	6.3	6.7	8.2	6.5	6.9

Kctd8::TranscriptClusterID:6938947

Exon.ID	665323	665323	665324	665330	665331	665334	665334	665334	665339	665340	665341	665345
mRNA.Accession	NM_175519	NM_175519	U15647	AK051136	AK051136	AK039273	AK039273	AK039273	AK083782	AK053635	AK053635	AK135393
Chromosome	chr5	chr5	chr5	chr5	chr5	chr5	chr5	chr5	chr5	chr5	chr5	chr5
Strand	-	-	-	-	-	-	-	-	-	-	-	-
Start	69500776	69501592	69512303	69560051	69560969	69572934	69573037	69573142	69610945	69613301	69615189	6965486
Stop	69501536	69501953	69512400	69560287	69561292	69573034	69573114	69573299	69611414	69613719	69615318	6965505
P30.WT.CSMN	4.3	5.4	9.5	4.3	4.1	2.7	4.5	4.1	5.7	4.9	6.1	5.6
P30.SOD1.CSMN	7.5	6.1	7.9	5.2	5.9	3	4.8	5.1	5.3	4.4	6.5	5.8

Exon.ID	665346	665349	665351	665352	665354	665354	665354	665354
mRNA.Accession	AK140687	ENSMUST00000087231	AK034505	AK034505	NM_175519	NM_175519	NM_175519	NM_175519
Chromosome	chr5	chr5	chr5	chr5	chr5	chr5	chr5	chr5
Strand	-	-	-	-	-	-	-	-
Start	69667210	69687488	69703015	69706693	69732029	69732445	69732510	69732567
Stop	69667423	69687865	69703579	69707173	69732376	69732486	69732539	69732816
P30.WT.CSMN	5.8	4.3	5.6	3.3	6.3	7.8	9.7	5.9
P30.SOD1.CSMN	6.1	4.6	6.5	6	6.7	9.2	10.9	7.3

Kctd9::TranscriptClusterID:6819957

Exon.ID	253873	253873	253873	253875	253876	253877	253878	253878	253879	253881	253882
mRNA.Accession	NM_001111028	NM_001111028	NM_001111028	NM_001111028	NM_001111028	NM_001111028	BC011302	NM_001111028	NM_001111028	NM_001111028	NM_001111028
Chromosome	chr14	chr14	chr14	chr14	chr14	chr14	chr14	chr14	chr14	chr14	chr14
Strand	+	+	+	+	+	+	+	+	+	+	+
Start	68334175	68334257	68334394	68342620	68344403	68346725	68347281	68347425	68347666	68351245	68351406
Stop	68334200	68334383	68334441	68342707	68344427	68346801	68347350	68347464	68347772	68351282	68351471
P30.WT.CSMN	9.8	7.8	7.8	6.6	9.3	7.2	2.8	6.1	6.9	7.8	5.4
P30.SOD1.CSMN	11.6	9.3	9.4	6.1	9.6	7.3	4.3	6.7	6.6	7.4	5.5

Exon.ID	253883	253883	253886	253886	253887	253887	253888	253888	253889
mRNA.Accession	NM_001111028	NM_001111028	NM_001111028	NM_001111028	NM_001111028	AK087312	NM_001111028	NM_001111028	NM_001111028
Chromosome	chr14	chr14	chr14	chr14	chr14	chr14	chr14	chr14	chr14
Strand	+	+	+	+	+	+	+	+	+
Start	68352211	68352258	68355691	68355733	68356460	68356743	68358353	68359183	68360271
Stop	68352257	68352333	68355720	68355759	68356566	68356978	68358447	68359785	68360335
P30.WT.CSMN	7	6.1	6.6	6.8	7.2	1.9	6.4	7.2	4.4
P30.SOD1.CSMN	7	6.5	6.8	5.6	7.5	5.2	7.2	8.2	5

Kctd10::TranscriptClusterID:6933600

Exon.ID	646088	646088	646090	646091	646092	646093	646094	646094
mRNA.Accession	ENSMUST00000031566	ENSMUST00000031566	ENSMUST00000031566	ENSMUST00000031566	ENSMUST00000031566	ENSMUST00000031566	ENSMUST00000031566	ENSMUST00000031566
Chromosome	chr5	chr5	chr5	chr5	chr5	chr5	chr5	chr5
Strand	+	+	+	+	+	+	+	+
Start	114806875	114806945	114807929	114808925	114809529	114809758	114810494	114810597
Stop	114806937	114806990	114808052	114808983	114809611	114809802	114810555	114810624
P30.WT.CSMN	5.6	5.4	3.4	4	3.9	3.4	7.7	2.7
P30.SOD1.CSMN	6.3	7.4	6	6.6	6.1	4.9	9.1	4.1

Exon.ID	646095	646095	646095	646095	646097	646098	646098	646099
mRNA.Accession	ENSMUST00000031566	ENSMUST00000031566	ENSMUST00000031566	ENSMUST00000031566	ENSMUST00000031566	ENSMUST00000031566	NM_001159941	NM_001159941
Chromosome	chr5	chr5	chr5	chr5	chr5	chr5	chr5	chr5
Strand	+	+	+	+	+	+	+	+
Start	114811065	114811123	114811171	114811272	114812121	114813101	114814020	114815139
Stop	114811120	114811160	114811221	114811333	114812197	114813568	114814487	114815477
P30.WT.CSMN	4.1	6.4	4.9	4.3	2.7	4.7	4	5.3
P30.SOD1.CSMN	7.3	7.9	6.6	6	4.6	5.2	5.2	5.9

Kctd10::TranscriptClusterID:6941202

Exon.ID	673653	673653	673655	673656	673657	673659	673660	673664
mRNA.Accession	NM_001159941	NM_001159941	NM_001159941	NM_001159941	NM_001159941	NM_001159941	NM_001159941	NM_001159941
Chromosome	chr5	chr5	chr5	chr5	chr5	chr5	chr5	chr5
Strand	-	-	-	-	-	-	-	-
Start	114814020	114815673	114817248	114818375	114818952	114820068	114824917	114830445
Stop	114815314	114815866	114817340	114818402	114819031	114820235	114825071	114830481
P30.WT.CSMN	7.6	5.4	7	6.7	5.5	7.5	5.6	5
P30.SOD1.CSMN	7.8	7.6	7.4	6.7	4.7	8.4	8.1	6.2

Kctd11::TranscriptClusterID:6789358

Exon.ID	147950	147951	147951	147952	147953	147954	147955	147955	147955	147955	147955	147956	147956	147956
mRNA.Accession	XM_978002	XM_978002	XM_978002	XM_978002	XM_978002	XM_978002	NM_153143	NM_153143	NM_153143	NM_153143	NM_153143	NM_153143	NM_153143	ENSMUST00000050555
Chromosome	chr11	chr11	chr11	chr11	chr11	chr11	chr11	chr11	chr11	chr11	chr11	chr11	chr11	chr11
Strand	-	-	-	-	-	-	-	-	-	-	-	-	-	-
Start	69690186	69690306	69690475	69690801	69691045	69691396	69691812	69693075	69693392	69693535	69693574	69693717	69694092	69694497
Stop	69690216	69690376	69690529	69690881	69691075	69691477	69692663	69693271	69693534	69693560	69693675	69693972	69694329	69694790
P30.WT.CSMN	4.6	5.4	6.2	4.9	3.5	4.2	5.3	5.6	5.1	2.8	5.4	4.6	5.7	2.9
P30.SOD1.CSMN	4.1	5.5	7.7	6	4.2	6.4	6.2	7.4	6.4	6.4	7.4	7.1	8	5.5

Kctd12::TranscriptClusterID:6827123

Exon.ID	277872	277873	277873	277874	277875	277875	277875
mRNA.Accession	NM_177715	NM_177715	NM_177715	NM_177715	NM_177715	NM_177715	NM_177715
Chromosome	chr14	chr14	chr14	chr14	chr14	chr14	chr14
Strand	-	-	-	-	-	-	-
Start	103375863	103376461	103377654	103380723	103381055	103381104	103381359
Stop	103376141	103377189	103377899	103380819	103381082	103381203	103381491
P30.WT.CSMN	4.1	6.7	6.7	7.2	7.9	7.8	6.5
P30.SOD1.CSMN	7.4	10	9.4	10.6	9.9	10.2	9.9

Kctd12b::TranscriptClusterID:7020314

Exon.ID	935302	935302	935303	935303	935304	935305
mRNA.Accession	NM_175429	NM_175429	NM_175429	NM_175429	NM_175429	NM_175429
Chromosome	chrX	chrX	chrX	chrX	chrX	chrX
Strand	-	-	-	-	-	-
Start	150119924	150121255	150123375	150123617	150130176	150130628
Stop	150120489	150122173	150123566	150124152	150130221	150130798
P30.WT.CSMN	4.5	5.1	5.3	4.4	3.5	4.2
P30.SOD1.CSMN	4.2	7.2	6.6	6.5	3.9	6.2

Kctd13::TranscriptClusterID:6964256

Exon.ID	754212	754212	754213	754216	754217	754218	754220	754220
mRNA.Accession	NM_172747	NM_172747	NM_172747	NM_172747	NM_172747	NM_172747	NM_172747	NM_172747
Chromosome	chr7	chr7	chr7	chr7	chr7	chr7	chr7	chr7
Strand	+	+	+	+	+	+	+	+
Start	134072434	134072599	134074167	134084857	134085025	134085680	134088405	134088705
Stop	134072557	134072804	134074303	134084910	134085055	134085845	134088631	134089079
P30.WT.CSMN	8.2	7.1	8.7	8.5	9.2	8.4	9.4	9.8
P30.SOD1.CSMN	9.4	7.6	9.7	9.9	10.1	8.6	10.1	9.9

Kctd14::TranscriptClusterID:6962759

Exon.ID	748070	748071	748071	748072	748072	748072	748073	748074	748075
mRNA.Accession	NM_001136235	NM_001012434	NM_001136235	NM_001012434	NM_001012434	NM_001012434	AK033025	NM_001136235	NM_001136235
Chromosome	chr7	chr7	chr7	chr7	chr7	chr7	chr7	chr7	chr7
Strand	+	+	+	+	+	+	+	+	+
Start	104599935	104601724	104601826	104603367	104603400	104603442	104603823	104606126	104607165
Stop	104600028	104601815	104601932	104603399	104603433	104603476	104604366	104606581	104607359
P30.WT.CSMN	4.8	4.8	5.2	2.9	4.6	5.5	5.4	5.4	5.4
P30.SOD1.CSMN	4.4	5.7	6.6	2.7	6.4	7.6	6.6	6	4.8

Kctd15::TranscriptClusterID:6966448

Exon.ID	763197	763197	763200	763202	763207	763208	763208	763209	763210	763213	763214
mRNA.Accession	NM_146188	NM_146188	NM_146188	NM_146188	NM_146188	NM_146188	NM_146188	NM_146188	NM_146188	ENSMUST00000108069	ENSMUST00000032709
Chromosome	chr7	chr7	chr7	chr7	chr7	chr7	chr7	chr7	chr7	chr7	chr7
Strand	-	-	-	-	-	-	-	-	-	-	-
Start	35424052	35425319	35426843	35429871	35435003	35435779	35435832	35437617	35437715	35439268	35440370
Stop	35424701	35425415	35426997	35429979	35435147	35435831	35435858	35437691	35437831	35439397	35440513
P30.WT.CSMN	6.8	7.1	6.6	4.7	6.5	6.2	8.5	6.2	5.2	7.3	6.2
P30.SOD1.CSMN	7.9	8	6.9	6.5	7.7	7.3	9.8	7.4	7.5	8.7	7.9

Kctd16::TranscriptClusterID:6860317

Exon.ID	393954	393954	393954	393959	393960	393961	393962	393962	393963	393974	393975	393976	393985	393986	393989	393989
mRNA.Accession	NM_026135	NM_026135	NM_026135	AK047720	AK081196	AK081196	AK081196	AK081196	AK081196	AK140247	AK158841	AK158841	AK015313	BC049734	NM_026135	NM_026135
Chromosome	chr18	chr18	chr18	chr18	chr18	chr18	chr18	chr18	chr18	chr18	chr18	chr18	chr18	chr18	chr18	chr18
Strand	+	+	+	+	+	+	+	+	+	+	+	+	+	+	+	+
Start	40418019	40418397	40418559	40505845	40507533	40509157	40509236	40509323	40511182	40574037	40576364	40578151	40672677	40678577	40690316	40690467
Stop	40418270	40418465	40418839	40506473	40507720	40509184	40509304	40509489	40511357	40574401	40576609	40578324	40672783	40678631	40690417	40690723
P30.WT.CSMN	7.4	7.5	8.1	3.1	5.4	1.3	6.1	3.5	3.7	5.6	6.2	4.5	4.4	2.5	7.6	8
P30.SOD1.CSMN	9.4	9.3	8.4	4.9	7	2.2	7.7	5.1	3.5	5.5	7.1	5.5	5.1	3.2	9.1	9.3

Kctd17::TranscriptClusterID:6831772

Exon.ID	292905	292906	292924	292934	292935	292945	292947	292947	292948	292949	292951	292952	292953	292957	292958	292958	292958	292958	292958
mRNA.Accession	XR_004836	XR_004836	AK137428	AK089409	AK089409	AK132810	BC144820	NM_001081367	NM_001081367	NM_001081367	NM_001081367	NM_001081367	NM_001081367	NM_001081367	NM_001081367	BC144820	BC144820	BC144820	NM_001081367
Chromosome	chr15	chr15	chr15	chr15	chr15	chr15	chr15	chr15	chr15	chr15	chr15	chr15	chr15	chr15	chr15	chr15	chr15	chr15	chr15
Strand	+	+	+	+	+	+	+	+	+	+	+	+	+	+	+	+	+	+	+
Start	77532481	77533171	77797022	78009874	78010507	78226527	78258951	78259109	78259205	78260474	78263429	78264348	78266024	78267352	78267400	78267590	78268018	78268059	78268971
Stop	77533080	77533269	77797252	78010360	78010875	78227552	78259070	78259141	78259296	78260545	78263483	78264439	78266136	78267379	78267424	78267926	78268046	78268100	78269691
P30.WT.CSMN	2.7	7.6	3.8	4.2	4.8	5.4	6.6	9.9	8.6	8.2	7.8	8.7	8	12	9.4	9	7.8	7.7	7.5
P30.SOD1.CSMN	5.1	9.2	5.8	6.8	5.9	6.9	8.2	11.1	9.7	8.7	8.5	9.6	8.8	13	10.2	10.1	9.9	9.5	9

Kctd18::TranscriptClusterID:6758920

Exon.ID	39609	39610	39610	39611	39611	39611	39611	39613	39614	39615	39615	39616	39617
mRNA.Accession	NR_027630	NR_027630	NR_027630	NR_027630	NR_027630	NR_027630	NR_027630	NR_027630	NM_030211	NR_027630	AK033212	AK033212	NR_027630
Chromosome	chr1	chr1	chr1	chr1	chr1	chr1	chr1	chr1	chr1	chr1	chr1	chr1	chr1
Strand	-	-	-	-	-	-	-	-	-	-	-	-	-
Start	58012041	58012196	58012989	58013047	58013140	58013208	58013494	58016003	58017133	58018747	58018858	58019222	58019954
Stop	58012088	58012868	58013014	58013131	58013167	58013241	58013633	58016086	58017898	58018834	58018926	58019711	58020060
P30.WT.CSMN	3.4	5.7	3.2	5	6.4	2.8	4.5	5.4	6.1	5.5	4	3	4.3
P30.SOD1.CSMN	2.4	5.5	3.1	6.7	6.9	4.6	5.7	6.5	6.4	6.2	5.8	5.8	4.7

	39618	39619	39619	39619	39619	39619	39619	39619	39619	39620	39629	39631	39632
	AK053098	NR_027630	AK053098	AK053098	AK053098	AK053098	NR_027630	NR_027630	NR_027630	NR_027630	AK019855	AK019855	AK019855
	chr1	chr1	chr1	chr1	chr1	chr1	chr1	chr1	chr1	chr1	chr1	chr1	chr1
	-	-	-	-	-	-	-	-	-	-	-	-	-
	58020547	58022236	58022508	58023564	58023886	58024300	58024361	58024498	58024534	58026255	58059739	58070453	58072546
	58020971	58022383	58023070	58023606	58024102	58024326	58024476	58024524	58024562	58026340	58059765	58070505	58074791
	4.6	5.1	6.2	4.4	5	7.6	6.5	5.7	4.6	6.9	5.2	3.6	4.1
	5.2	5.5	7.1	6.7	5	7.7	7.6	6	5.2	8.3	5.3	3.5	6.7

Kctd19::TranscriptClusterID:6984971

Exon.ID	827387	827387	827388	827389	827390	827391	827391	827391	827392	827393	827394
mRNA.Accession	NM_177791	NM_177791	NM_177791	NM_177791	NM_177791	NM_177791	NM_177791	NM_177791	NM_177791	NM_177791	NM_177791
Chromosome	chr8	chr8	chr8	chr8	chr8	chr8	chr8	chr8	chr8	chr8	chr8
Strand	-	-	-	-	-	-	-	-	-	-	-
Start	107906743	107906863	107908634	107908923	107909274	107910723	107910807	107911153	107911708	107912092	107912414
Stop	107906850	107906968	107908709	107909031	107909398	107910753	107911100	107911232	107911746	107912151	107912501
P30.WT.CSMN	5.3	3.5	3.5	2.7	5.8	6.1	4.5	4.3	4.6	4.8	5.6
P30.SOD1.CSMN	4.2	5.4	6.6	7	6.7	7.1	7	5.8	5.8	6.1	6.7

Exon.ID	827395	827396	827399	827400	827401	827402	827403	827405	827407
mRNA.Accession	NM_177791	NM_177791	NM_177791	NM_177791	NM_177791	NM_177791	NM_177791	NM_177791	NM_177791
Chromosome	chr8	chr8	chr8	chr8	chr8	chr8	chr8	chr8	chr8
Strand	-	-	-	-	-	-	-	-	-
Start	107912955	107914203	107915794	107917501	107917729	107919189	107920247	107932235	107937362
Stop	107913038	107914313	107915963	107917527	107917852	107919346	107920339	107932501	107937399
P30.WT.CSMN	4	5.8	5.2	6.3	2	5.2	5.3	4.5	5.9
P30.SOD1.CSMN	4.4	6.9	6	8	3.2	6.8	6.3	6	7.5

Kctd20::TranscriptClusterID:6849581

Exon.ID	356379	356380	356380	356381	356382	356385	356385	356385	356386	356387	356388	356389	356390	356390	356390	356391
mRNA.Accession	ENSMUST00000057174	ENSMUST00000122163	NM_025888	AK045670	NM_025888	NM_025888	NM_025888	NM_025888	NM_025888	NM_025888	NM_025888	NM_025888	NM_025888	NM_025888	NM_025888	ENSMUST00000118762
Chromosome	chr17	chr17	chr17	chr17	chr17	chr17	chr17	chr17	chr17	chr17	chr17	chr17	chr17	chr17	chr17	chr17
Strand	+	+	+	+	+	+	+	+	+	+	+	+	+	+	+	+
Start	29089414	29089642	29089839	29092631	29094863	29098359	29098419	29098586	29099762	29100227	29100914	29101980	29103621	29103664	29104209	29104937
Stop	29089507	29089693	29090197	29093285	29094996	29098396	29098581	29098632	29099857	29100310	29100985	29102011	29103646	29103863	29104838	29106148
P30.WT.CSMN	8	7.6	6.3	6.9	5.6	7.2	7.7	6.3	8.2	7	7.8	7.9	9.6	7.3	7.2	6.4
P30.SOD1.CSMN	10	9.2	6.1	7.5	7.2	8.8	8.2	6.9	9.3	8	8.4	8.7	10.9	8.4	8.1	7.3

Kctd20::TranscriptClusterID:6965688

Exon.ID	759979	759980	759981	759982	759982	759982
mRNA.Accession	NM_025888	NM_025888	NM_025888	NM_025888	NM_025888	XR_033421
Chromosome	chr7	chr7	chr7	chr7	chr7	chr7
Strand	-	-	-	-	-	-
Start	18270635	18270892	18271222	18271399	18271509	18271626
Stop	18270688	18271008	18271306	18271424	18271540	18271656
P30.WT.CSMN	5.4	5.6	6.2	7.3	7.9	7.4
P30.SOD1.CSMN	5.5	6.5	7.5	8.7	8.5	8.3

Kctd21::TranscriptClusterID:6962748

Exon.ID	748029	748029	748029	748030	748031
mRNA.Accession	NM_001039039	NM_001039039	NM_001039039	NM_001039039	NM_001039039
Chromosome	chr7	chr7	chr7	chr7	chr7
Strand	+	+	+	+	+
Start	104496612	104496809	104497005	104497536	104498494
Stop	104496721	104496970	104497126	104498250	104498688
P30.WT.CSMN	6.8	3.9	4.4	6.2	6.1
P30.SOD1.CSMN	7.8	6.6	5.9	6.8	6.9

Scn1a::TranscriptClusterID:6887327

Exon.ID	490749	490756	490757	490758	490764	490765	490766	490769	490770	490771	490772	490773	490774	490775	490776	490776	490777	490778	490779	490780
mRNA.Accession	AK144548	AK034330	AK034330	AK034330	NM_018733	NM_018733	NM_018733	NM_018733	NM_018733	NM_018733	NM_018733	NM_018733	NM_018733	NM_018733	NM_018733	NM_018733	NM_018733	NM_018733	NM_018733	NM_018733
Chromosome	chr2	chr2	chr2	chr2	chr2	chr2	chr2	chr2	chr2	chr2	chr2	chr2	chr2	chr2	chr2	chr2	chr2	chr2	chr2	chr2
Strand	-	-	-	-	-	-	-	-	-	-	-	-	-	-	-	-	-	-	-	-
Start	65781521	65855536	65856811	65857276	66108922	66115675	66118753	66123979	66125788	66126981	66134861	66137713	66140423	66141650	66155874	66156022	66157478	66158925	66160311	66161391
Stop	65781836	65855628	65856977	65857693	66109599	66115782	66118810	66124104	66125813	66127201	66134932	66137843	66140576	66141734	66155900	66156313	66157798	66159081	66160534	66161501
P30.WT.CSMN	3.9	5.5	6.3	5.8	10.7	10.5	10.1	10.3	10.3	10.8	10.6	9.4	10.4	8.2	7.3	10.2	10.5	9	7.9	7.9
P30.SOD1.CSMN	5.6	5.2	7.4	6.1	10.6	10.8	9.6	9.7	10.7	10.3	10.5	9	9.9	8.1	8.5	11.1	10.9	9.8	8.6	8.2

Exon.ID	490781	490781	490783	490784	490786	490787	490788	490789	490790	490792	490794	490795	490796	490797	490798	490800	490800	490800	490803	490805	490806
mRNA.Accession	NM_018733	NM_018733	NM_018733	NM_018733	NM_018733	NM_018733	NM_018733	NM_018733	NM_018733	NM_018733	NM_018733	AK053066	AK053066	AK159058	AK159058	AK046001	AK046001	NM_018733	AK054148	ENSMUST00000112366	NM_018733
Chromosome	chr2	chr2	chr2	chr2	chr2	chr2	chr2	chr2	chr2	chr2	chr2	chr2	chr2	chr2	chr2	chr2	chr2	chr2	chr2	chr2	chr2
Strand	-	-	-	-	-	-	-	-	-	-	-	-	-	-	-	-	-	-	-	-	-
Start	66162664	66162983	66163989	66165725	66166462	66167113	66169313	66170271	66172267	66173508	66175914	66176985	66180763	66181949	66182334	66186720	66187728	66189116	66213492	66247942	66278793
Stop	66162948	66163008	66164168	66165904	66166552	66167142	66169477	66170300	66172355	66173540	66175983	66177243	66181063	66182211	66182554	66187512	66188658	66189235	66213691	66248049	66278872
P30.WT.CSMN	9.9	10.2	7.9	9.3	8.2	9.3	8.4	7.5	8.3	7.5	7	6.7	10.6	8.1	7.5	7.6	8.6	9.5	6.6	8.1	7.8
P30.SOD1.CSMN	10.9	11.2	8.5	10.1	9.8	10.4	9.5	8.5	9.2	8.8	8	7.3	10.7	9.2	9.5	9	9.4	10.4	6.5	9.6	8.3

Scn2a1::TranscriptClusterID:6877739

Exon.ID	455833	455834	455838	455839	455840	455841	455841	455841	455842	455846	455847	455848	455849	455850	455851	455852
mRNA.Accession	ENSMUST00000028377	AK035787	DQ993535	NM_001099298	AK143234	AK143234	NM_001099298	AK143234	AK134953	NM_001099298	NM_001099298	BC158048	NM_001099298	NM_001099298	NM_001099298	NM_001099298
Chromosome	chr2	chr2	chr2	chr2	chr2	chr2	chr2	chr2	chr2	chr2	chr2	chr2	chr2	chr2	chr2	chr2
Strand	+	+	+	+	+	+	+	+	+	+	+	+	+	+	+	+
Start	65459000	65460716	65506833	65508506	65509264	65509621	65509680	65509772	65514365	65519076	65520095	65520798	65521924	65524854	65526375	65526665
Stop	65459485	65461174	65506871	65508632	65509291	65509658	65509764	65509803	65514675	65519103	65520155	65520829	65521986	65524904	65526500	65526802
P30.WT.CSMN	6	6.9	7.5	9.3	6.5	7.3	10.1	1.8	6.9	8.6	7.9	9	8.8	9.9	8.3	10.7
P30.SOD1.CSMN	6.5	6.5	6.6	9.7	7.1	6.6	10.4	2.4	6	9.5	9	10	8.7	10.5	8.6	11.3

455852	455853	455853	455853	455858	455859	455862	455863	455864	455865	455868	455869	455870	455871	455872	455873
NM_001099298	NM_001099298	NM_001099298	NM_001099298	NM_001099298	NM_001099298	NM_001099298	NM_001099298	NM_001099298	NM_001099298	NM_001099298	NM_001099298	NM_001099298	NM_001099298	NM_001099298	NM_001099298
chr2	chr2	chr2	chr2	chr2	chr2	chr2	chr2	chr2	chr2	chr2	chr2	chr2	chr2	chr2	chr2
+	+	+	+	+	+	+	+	+	+	+	+	+	+	+	+
65526665	65528108	65528154	65528308	65539811	65545331	65549790	65551755	65553883	65555476	65564394	65566456	65568167	65571294	65573672	65581148
65526802	65528137	65528302	65528373	65539978	65545413	65549894	65551892	65554036	65555763	65564494	65566595	65568336	65571398	65573934	65581172
10.7	8.8	9.9	9.2	10.5	9.7	8.3	7.4	10.1	11.4	10.2	10.6	11.8	10.2	11.6	11.6
11.3	9.2	10.3	9.5	11	10.2	8.7	8	11.2	12	10.5	10.9	12.3	10.6	12.3	12.2

455874	455875	455876	455880	455880	455880	455881	455881	455882	455882	455882	455882	455882	455882
NM_001099298	NM_001099298	NM_001099298	NM_001099298	NM_001099298	NM_001099298	NM_001099298	NM_001099298	NM_001099298	NM_001099298	NM_001099298	NM_001099298	NM_001099298	NM_00109929
chr2	chr2	chr2	chr2	chr2	chr2	chr2	chr2	chr2	chr2	chr2	chr2	chr2	chr2
+	+	+	+	+	+	+	+	+	+	+	+	+	+
65586351	65586907	65589925	65602036	65602640	65602682	65602761	65602846	65602934	65603009	65603068	65603139	65603502	65605264
65586458	65586933	65590139	65602629	65602667	65602734	65602799	65602877	65602991	65603036	65603138	65603182	65604841	65605452
10.9	10.7	8.6	10.1	1.5	9.3	9.6	9.8	9.8	2.8	10.5	9.7	9.4	6
11.5	11	9.5	10.5	3.3	9.6	10.7	9.6	10.7	3.4	11	10.7	9.9	7.4

Scn2a1::TranscriptClusterID:6887374

Exon.ID	490931	490931	490931	490932	490933	490935	490936	490937	490938	490940	490941	490942	490944	490945	490946	490946	490947	490947	490948	490948	490949
mRNA.Accession	NM_018852	NM_018852	NM_001099298	NM_018852	NM_018852	NM_018852	NM_018852	NM_018852	NM_018852	NM_018852	NM_018852	NM_018852	AK051636	AK051636	NM_018852	NM_018852	ENSMUST00000119700	ENSMUST00000119700	NM_018852	NM_001099298	NM_018852
Chromosome	chr2	chr2	chr2	chr2	chr2	chr2	chr2	chr2	chr2	chr2	chr2	chr2	chr2	chr2	chr2	chr2	chr2	chr2	chr2	chr2	chr2
Strand	-	-	-	-	-	-	-	-	-	-	-	-	-	-	-	-	-	-	-	-	-
Start	66318847	66321651	66322540	66325473	66326040	66331181	66331949	66332671	66339675	66342945	66346602	66353373	66358803	66359995	66364874	66365084	66368590	66368650	66371139	66371446	66372181
Stop	66321451	66321759	66322599	66325634	66326065	66331267	66331973	66332942	66339786	66343048	66346708	66353458	66359135	66360574	66365055	66365108	66368625	66368723	66371299	66371471	66372344
P30.WT.CSMN	4.3	3.3	10.7	1.7	5.6	3.2	6.8	6	2.6	6.2	5.5	3.8	6.1	3.7	4.5	2.4	2.1	4.2	4	10.2	6
P30.SOD1.CSMN	5.9	5.4	11	3.8	6.6	3.8	8.2	6.8	3.2	5.5	5	6.3	6.7	3.9	4.9	1.5	3.5	4.3	5.2	10.6	5.6

490951	490951	490952	490953	490954	490955	490955	490956	490957	490958	490963	490964	490965	490966	490966	490968	490969	490970	490971
NM_018852	NM_018852	NM_018852	NM_018852	NM_018852	NM_018852	NM_018852	NM_018852	NM_018852	NM_018852	NM_018852	NM_018732	NM_001099298	AK141871	NM_018852	NM_018852	NM_018852	NM_018852	NM_01885
chr2	chr2	chr2	chr2	chr2	chr2	chr2	chr2	chr2	chr2	chr2	chr2	chr2	chr2	chr2	chr2	chr2	chr2	chr2
-	-	-	-	-	-	-	-	-	-	-	-	-	-	-	-	-	-	-
66374168	66374260	66375336	66378476	66381127	66385044	66385103	66387061	66388401	66389745	66400446	66401402	66401604	66402840	66403200	66403874	66404231	66406167	66472687
66374254	66374322	66375456	66378686	66381360	66385072	66385233	66387172	66388458	66389773	66400530	66401433	66401668	66403082	66403288	66403906	66404321	66406298	66472962
3.2	2	5.6	7.1	5.2	6.7	6.1	6.1	3.7	4.4	3.6	8.7	5	3.7	5.1	4.4	2.4	4.7	5.5
2.1	1.7	4.6	6.9	5.6	4.2	4.9	6.1	3.3	4.8	3.6	9.8	4	4.3	4.8	4.1	3.9	5.3	6.2

Scn3a::TranscriptClusterID:6887311

Exon.ID	490692
mRNA.Accession	AK141113
Chromosome	chr2
Strand	-
Start	65305286
Stop	65305557
P30.WT.CSMN	4.3
P30.SOD1.CSMN	4.8

Scn3a::TranscriptClusterID:6887315

Exon.ID	490696
mRNA.Accession	AK148712
Chromosome	chr2
Strand	-
Start	65307269
Stop	65307929
P30.WT.CSMN	4.7
P30.SOD1.CSMN	3.7

Scn3a::TranscriptClusterID:6887320

Exon.ID	490701
mRNA.Accession	AK148712
Chromosome	chr2
Strand	-
Start	65320814
Stop	65320902
P30.WT.CSMN	6.6
P30.SOD1.CSMN	6.1

Scn3a::TranscriptClusterID:6887324

Exon.ID	490706	490706	490707	490707	490707	490708	490711	490712	490713	490713	490714	490715	490716
mRNA.Accession	L42337	NM_018732	NM_018732	NM_018732	L42337	NM_018732	NM_018732	NM_018732	ENSMUST00000066432	ENSMUST00000066432	NM_018732	NM_018732	NM_018732
Chromosome	chr2	chr2	chr2	chr2	chr2	chr2	chr2	chr2	chr2	chr2	chr2	chr2	chr2
Strand	-	-	-	-	-	-	-	-	-	-	-	-	-
Start	65331215	65333053	65335446	65335754	65335803	65336924	65344132	65345204	65346332	65346372	65346464	65352537	65358923
Stop	65332186	65333438	65335473	65335779	65336505	65337089	65344263	65345307	65346368	65346420	65346597	65352654	65359072
P30.WT.CSMN	5.3	7.3	6.3	10.3	2.2	7.3	5.5	6.3	2.6	6.4	6.1	4.2	4.8
P30.SOD1.CSMN	6.4	7.5	6.7	10.7	3.7	7.9	7.1	5.5	2.9	6.5	7.1	6.2	5.1

Exon.ID	490717	490718	490719	490720	490721	490721	490722	490722	490723	490724	490726	490726	490728
mRNA.Accession	NM_018732	NM_018732	NM_018732	NM_018732	ENSMUST00000112407	ENSMUST00000112407	NM_018732	NM_018732	NM_018732	NM_018732	NM_018732	NM_018732	NM_018732
Chromosome	chr2	chr2	chr2	chr2	chr2	chr2	chr2	chr2	chr2	chr2	chr2	chr2	chr2
Strand	-	-	-	-	-	-	-	-	-	-	-	-	-
Start	65359867	65362235	65362661	65363761	65363941	65363981	65364582	65364653	65367447	65368902	65374589	65374739	65405337
Stop	65359989	65362260	65362796	65363789	65363970	65364025	65364613	65364679	65367495	65368958	65374699	65374783	65405439
P30.WT.CSMN	6.7	4.9	6.2	8.9	4.5	4.2	6.3	2.6	6	6.8	7.9	6	7
P30.SOD1.CSMN	6.4	3.9	6.8	9.9	5	3.6	6.1	4.9	6.5	5.8	8.3	3.7	8.2

Scn3a::TranscriptClusterID:6887374

Exon.ID	490931	490931	490931	490932	490933	490935	490936	490937	490938	490940	490941	490942	490944	490945	490946	490946	490947	490947	490948
mRNA.Accession	NM_018852	NM_018852	NM_001099298	NM_018852	NM_018852	NM_018852	NM_018852	NM_018852	NM_018852	NM_018852	NM_018852	NM_018852	AK051636	AK051636	NM_018852	NM_018852	ENSMUST00000119700	ENSMUST00000119700	NM_018852
Chromosome	chr2	chr2	chr2	chr2	chr2	chr2	chr2	chr2	chr2	chr2	chr2	chr2	chr2	chr2	chr2	chr2	chr2	chr2	chr2
Strand	-	-	-	-	-	-	-	-	-	-	-	-	-	-	-	-	-	-	-
Start	66318847	66321651	66322540	66325473	66326040	66331181	66331949	66332671	66339675	66342945	66346602	66353373	66358803	66359995	66364874	66365084	66368590	66368650	66371139
Stop	66321451	66321759	66322599	66325634	66326065	66331267	66331973	66332942	66339786	66343048	66346708	66353458	66359135	66360574	66365055	66365108	66368625	66368723	66371299
P30.WT.CSMN	4.3	3.3	10.7	1.7	5.6	3.2	6.8	6	2.6	6.2	5.5	3.8	6.1	3.7	4.5	2.4	2.1	4.2	4
P30.SOD1.CSMN	5.9	5.4	11	3.8	6.6	3.8	8.2	6.8	3.2	5.5	5	6.3	6.7	3.9	4.9	1.5	3.5	4.3	5.2

Exon.ID	490948	490949	490951	490951	490952	490953	490954	490955	490955	490956	490957	490958	490963	490964	490965	490966	490966	490968	490969	490970	490971
mRNA.Accession	NM_001099298	NM_018852	NM_018852	NM_018852	NM_018852	NM_018852	NM_018852	NM_018852	NM_018852	NM_018852	NM_018852	NM_018852	NM_018852	NM_018732	NM_001099298	AK141871	NM_018852	NM_018852	NM_018852	NM_018852	NM_018852
Chromosome	chr2	chr2	chr2	chr2	chr2	chr2	chr2	chr2	chr2	chr2	chr2	chr2	chr2	chr2	chr2	chr2	chr2	chr2	chr2	chr2	chr2
Strand	-	-	-	-	-	-	-	-	-	-	-	-	-	-	-	-	-	-	-	-	-
Start	66371446	66372181	66374168	66374260	66375336	66378476	66381127	66385044	66385103	66387061	66388401	66389745	66400446	66401402	66401604	66402840	66403200	66403874	66404231	66406167	66472687
Stop	66371471	66372344	66374254	66374322	66375456	66378686	66381360	66385072	66385233	66387172	66388458	66389773	66400530	66401433	66401668	66403082	66403288	66403906	66404321	66406298	66472962
P30.WT.CSMN	10.2	6	3.2	2	5.6	7.1	5.2	6.7	6.1	6.1	3.7	4.4	3.6	8.7	5	3.7	5.1	4.4	2.4	4.7	5.5
P30.SOD1.CSMN	10.6	5.6	2.1	1.7	4.6	6.9	5.6	4.2	4.9	6.1	3.3	4.8	3.6	9.8	4	4.3	4.8	4.1	3.9	5.3	6.2

Scn4a::TranscriptClusterID:6791915

Exon.ID	157973	157973	157973	157973	157973	157973	157974	157975	157978	157979	157980	157981	157982	157983	157984	157985	157986
mRNA.Accession	NM_133199	NM_133199	NM_133199	NM_133199	NM_133199	NM_133199	NM_133199	NM_133199	NM_133199	NM_133199	NM_133199	NM_133199	NM_133199	NM_133199	NM_133199	NM_133199	NM_133199
Chromosome	chr11	chr11	chr11	chr11	chr11	chr11	chr11	chr11	chr11	chr11	chr11	chr11	chr11	chr11	chr11	chr11	chr11
Strand	-	-	-	-	-	-	-	-	-	-	-	-	-	-	-	-	-
Start	106180077	106180353	106180666	106180983	106181110	106181586	106183273	106184102	106185213	106185522	106185775	106187455	106188146	106188871	106189336	106191318	106196821
Stop	106180333	106180557	106180919	106181088	106181135	106182186	106183487	106184127	106185319	106185573	106185878	106187561	106188185	106188970	106189462	106191486	106197030
P30.WT.CSMN	5.1	4.9	4.6	6.8	5.3	5.2	3.9	7	2.7	4.1	4.9	5	5.3	5.5	5.3	5.9	4.5
P30.SOD1.CSMN	6.3	6.9	6.3	8	2.9	7.3	5.5	8.8	4.9	5.9	7	7.4	6.8	7.3	6.4	7.6	7.1

	157987	157988	157989	157990	157991	157992	157993	157994	157995	157996	157998	157999	157999	158000	158002
	NM_133199	NM_133199	NM_133199	NM_133199	NM_133199	NM_133199	NM_133199	NM_133199	NM_133199	NM_133199	NM_133199	NM_133199	NM_133199	ENSMUST00000106818	ENSMUST00000106818
	chr11	chr11	chr11	chr11	chr11	chr11	chr11	chr11	chr11	chr11	chr11	chr11	chr11	chr11	chr11
	-	-	-	-	-	-	-	-	-	-	-	-	-	-	-
	106198374	106200478	106202745	106203713	106205236	106205552	106206746	106209071	106209623	106210017	106210214	106210495	106210642	106210756	106226798
	106198503	106200654	106202819	106203826	106205343	106205591	106207002	106209155	106209688	106210087	106210323	106210634	106210666	106210998	106226830
	5.3	4.5	3.6	4.7	2.8	4.3	4.3	5.6	4.2	3.7	5.3	3.9	8.5	5.5	7.8
	7.1	6	5.6	6	5.6	6.6	5.6	7.3	5.5	4.9	6.7	6.3	9.9	7.6	9.8

Scn5a::TranscriptClusterID:6999438

Exon.ID	879694	879694	879697	879698	879699	879700	879701	879702	879703	879704	879705	879707	879708	879711	879712	879714	879715
mRNA.Accession	NM_021544	NM_021544	NM_021544	NM_021544	NM_021544	NM_021544	NM_021544	NM_021544	NM_021544	NM_021544	NM_021544	NM_021544	NM_021544	NM_021544	NM_021544	NM_021544	NM_021544
Chromosome	chr9	chr9	chr9	chr9	chr9	chr9	chr9	chr9	chr9	chr9	chr9	chr9	chr9	chr9	chr9	chr9	chr9
Strand	-	-	-	-	-	-	-	-	-	-	-	-	-	-	-	-	-
Start	119392702	119394739	119398867	119400579	119401187	119402180	119404664	119407208	119410816	119421292	119422125	119425060	119426533	119430298	119431601	119438128	119439075
Stop	119394674	119395322	119399131	119400674	119401294	119402232	119404800	119407303	119410890	119421409	119422214	119425114	119426809	119430359	119431742	119438261	119439207
P30.WT.CSMN	5.1	6.7	8.4	6	7	8.2	6.9	5.8	6.6	6	6.2	5.5	6.4	6.1	6.4	6.8	6.6
P30.SOD1.CSMN	7.5	7.4	6.8	6	6.7	8.3	6.3	6.9	6.7	7.4	7.5	7.2	7.3	6.9	7.8	7.2	8.3

	879716	879717	879718	879719	879720	879722	879723	879724	879725	879727	879733	879734	879735	879735	879744	879744
	NM_021544	NM_021544	NM_021544	NM_021544	NM_021544	NM_021544	ENSMUST00000120420	NM_021544	NM_021544	NM_021544	NM_021544	NM_021544	NM_021544	NM_021544	NM_021544	NM_021544
	chr9	chr9	chr9	chr9	chr9	chr9	chr9	chr9	chr9	chr9	chr9	chr9	chr9	chr9	chr9	chr9
	-	-	-	-	-	-	-	-	-	-	-	-	-	-	-	-
	119443019	119443673	119444957	119445590	119446741	119448651	119452220	119452482	119459745	119461104	119469388	119469465	119471613	119471743	119487967	119488036
	119443102	119443852	119445127	119445698	119446792	119448828	119452284	119452522	119459833	119461156	119469414	119469491	119471739	119471772	119488033	119488091
	5.6	6.4	5.5	5.8	6	7.2	6.8	6.1	6.3	6.4	8.2	8.9	6.1	7.5	10	9.1
	5.6	6.7	6.7	7.5	7.4	7.3	7.4	6	6.6	6.8	8	7.3	7.2	8.4	11.7	10.2

Scn7a::TranscriptClusterID:6887380

Exon.ID	490981	490981	490982	490983	490984	490985	490986	490987	490988	490988	490989	490989	490990	490991	490992	490993
mRNA.Accession	NM_009135	NM_009135	NM_009135	NM_009135	NM_009135	NM_009135	NM_009135	NM_009135	NM_009135	NM_009135	NM_009135	NM_009135	NM_009135	NM_009135	NM_009135	NM_009135
Chromosome	chr2	chr2	chr2	chr2	chr2	chr2	chr2	chr2	chr2	chr2	chr2	chr2	chr2	chr2	chr2	chr2
Strand	-	-	-	-	-	-	-	-	-	-	-	-	-	-	-	-
Start	66511945	66513876	66518194	66518931	66520770	66521332	66522113	66525807	66527589	66527701	66530614	66530693	66532883	66535674	66538004	66538801
Stop	66513034	66514566	66518389	66519018	66520870	66521364	66522299	66525888	66527697	66527762	66530641	66530717	66532968	66536033	66538212	66538925
P30.WT.CSMN	5.9	3.1	3.2	4.6	3	1.1	3.2	3.4	3	1.6	3.7	3.8	1.7	4	1.9	3.1
P30.SOD1.CSMN	6.2	5.3	4.5	6.3	4.5	3.6	6.1	3.8	4.1	2	3.1	2.9	3.6	5.8	4.5	3.9

Exon.ID	490994	490994	490995	490996	490997	490998	490999	491000	491001	491003	491003	491004	491005	491005	491006
mRNA.Accession	NM_009135	NM_009135	NM_009135	AK142964	NM_009135	NM_009135	NM_009135	NM_009135	NM_009135	NM_009135	NM_009135	NM_009135	NM_009135	NM_009135	NM_009135
Chromosome	chr2	chr2	chr2	chr2	chr2	chr2	chr2	chr2	chr2	chr2	chr2	chr2	chr2	chr2	chr2
Strand	-	-	-	-	-	-	-	-	-	-	-	-	-	-	-
Start	66541760	66541811	66543097	66551295	66551912	66564216	66567069	66575451	66580038	66581760	66581834	66589933	66590253	66590335	66595218
Stop	66541808	66541899	66543132	66551722	66552064	66564352	66567126	66575632	66580092	66581789	66581888	66590014	66590327	66590371	66595382
P30.WT.CSMN	4.1	3.5	4.6	3.4	2.3	2.8	3.4	4.7	2.2	2.7	2.8	1.6	5.1	2.8	2.3
P30.SOD1.CSMN	5.1	4.2	3.7	5.5	6	5.2	6.1	7.2	2.1	3.2	2.8	2.5	4.8	4.2	3.6

Scn8a::TranscriptClusterID:6833286

Exon.ID	298659	298662	298662	298663	298666	298669	298670	298671	298672	298678	298679	298680
mRNA.Accession	NM_001077499	NM_001077499	NM_001077499	BC099466	NM_001077499	NM_001077499	NM_001077499	NM_001077499	NM_011323	NM_001077499	NM_001077499	NM_001077499
Chromosome	chr15	chr15	chr15	chr15	chr15	chr15	chr15	chr15	chr15	chr15	chr15	chr15
Strand	+	+	+	+	+	+	+	+	+	+	+	+
Start	100701134	100766717	100766818	100770574	100785888	100787503	100787892	100789802	100790085	100799508	100800637	100801960
Stop	100701174	100766741	100767020	100770784	100785943	100787548	100787983	100789842	100790109	100799664	100800697	100802092
P30.WT.CSMN	13.1	9.2	10.1	8	7.9	7	8.8	8.1	9.2	9.8	10	8.4
P30.SOD1.CSMN	13.8	9.7	10.6	7.8	9.3	7.4	9	7.4	10.2	10.6	10.7	8.3

298680	298681	298681	298682	298686	298686	298686	298687	298687	298690	298691	298692	298693
NM_001077499	NM_001077499	NM_001077499	NM_001077499	NM_001077499	NM_001077499	NM_001077499	NM_001077499	ENSMUST00000082209	AK047470	AK047470	AK140587	NM_001077499
chr15	chr15	chr15	chr15	chr15	chr15	chr15	chr15	chr15	chr15	chr15	chr15	chr15
+	+	+	+	+	+	+	+	+	+	+	+	+
100801960	100803193	100803339	100804858	100814131	100814174	100814268	100814382	100814477	100820418	100821748	100832128	100832465
100802092	100803261	100803363	100805120	100814155	100814254	100814354	100814470	100814502	100820899	100822109	100832290	100832490
8.4	10.5	8.6	9.5	8.5	8.2	8.3	10.2	6.2	8.6	7.4	1.4	9.7
8.3	10.9	9.5	9.8	10	8.6	9.4	11	4.2	8.5	6.5	2.6	10

298693	298694	298695	298698	298698	298699	298700	298701	298702	298702	298706
NM_001077499	NM_001077499	NM_001077499	NM_001077499	NM_001077499	NM_001077499	NM_001077499	NM_001077499	NM_001077499	NM_001077499	ENSMUST00000108907
chr15	chr15	chr15	chr15	chr15	chr15	chr15	chr15	chr15	chr15	chr15
+	+	+	+	+	+	+	+	+	+	+
100832493	100838586	100841523	100843670	100843823	100846209	100846930	100847493	100848815	100848928	100854270
100832595	100838715	100841621	100843694	100844000	100846504	100846974	100847551	100848916	100848955	100854303
10.4	9.9	9.2	9.9	9.7	9.3	8.4	10.5	8.6	10.6	6.1
11.3	10.4	9.7	10.4	10.7	10.3	8.7	10.7	8.9	11.7	5.6

298707	298709	298709	298709	298711	298712	298714	298715	298716	298719	298719	298720
NM_001077499	AK036303	AK036303	AK036303	NM_001077499	NM_001077499	NM_001077499	NM_001077499	NM_001077499	NM_001077499	NM_001077499	NM_001077499
chr15	chr15	chr15	chr15	chr15	chr15	chr15	chr15	chr15	chr15	chr15	chr15
+	+	+	+	+	+	+	+	+	+	+	+
100854701	100858291	100858766	100858885	100860090	100862167	100862616	100863944	100865890	100870102	100871438	100872904
100854781	100858413	100858805	100859379	100860275	100862194	100862730	100864013	100866025	100870902	100872733	100874616
9.7	8.9	8.4	6.8	11	10.3	10.8	8.8	10.1	11.9	11.2	11.5
10.2	8.6	7.4	6.7	11.4	10.8	11.5	9	10.5	12.3	11.7	12.1

Scn9a::TranscriptClusterID:6877767

Exon.ID	456002	456006	456007	456008	456009	456010
mRNA.Accession	AK039017	NM_018852	NM_018852	AK039017	AK039017	EF410143
Chromosome	chr2	chr2	chr2	chr2	chr2	chr2
Strand	+	+	+	+	+	+
Start	66278976	66318576	66322450	66340504	66351014	66383628
Stop	66279283	66318792	66322612	66340533	66351234	66383893
P30.WT.CSMN	5.8	3.7	8.8	6	6.9	6.8
P30.SOD1.CSMN	7	3.9	8	5.2	6.9	7.3

Scn9a::TranscriptClusterID:6877772

Exon.ID	456024
mRNA.Accession	NM_018852
Chromosome	chr2
Strand	+
Start	66385179
Stop	66385298
P30.WT.CSMN	7.8
P30.SOD1.CSMN	8.3

Scn9a::TranscriptClusterID:6877776

Exon.ID	456032	456032
mRNA.Accession	NM_018852	NM_018852
Chromosome	chr2	chr2
Strand	+	+
Start	66472864	66472919
Stop	66472907	66472956
P30.WT.CSMN	6.8	4.4
P30.SOD1.CSMN	8.4	5.7

Scn9a::TranscriptClusterID:6877739

Exon.ID	455833	455834	455838	455839	455840	455841	455841	455841	455842	455846	455847	455848	455849	455850	455851	455852	455853
mRNA.Accession	ENSMUST00000028377	AK035787	DQ993535	NM_001099298	AK143234	AK143234	NM_001099298	AK143234	AK134953	NM_001099298	NM_001099298	BC158048	NM_001099298	NM_001099298	NM_001099298	NM_001099298	NM_001099298
Chromosome	chr2	chr2	chr2	chr2	chr2	chr2	chr2	chr2	chr2	chr2	chr2	chr2	chr2	chr2	chr2	chr2	chr2
Strand	+	+	+	+	+	+	+	+	+	+	+	+	+	+	+	+	+
Start	65459000	65460716	65506833	65508506	65509264	65509621	65509680	65509772	65514365	65519076	65520095	65520798	65521924	65524854	65526375	65526665	65528108
Stop	65459485	65461174	65506871	65508632	65509291	65509658	65509764	65509803	65514675	65519103	65520155	65520829	65521986	65524904	65526500	65526802	65528137
P30.WT.CSMN	6	6.9	7.5	9.3	6.5	7.3	10.1	1.8	6.9	8.6	7.9	9	8.8	9.9	8.3	10.7	8.8
P30.SOD1.CSMN	6.5	6.5	6.6	9.7	7.1	6.6	10.4	2.4	6	9.5	9	10	8.7	10.5	8.6	11.3	9.2

Exon.ID	455853	455853	455853	455858	455859	455862	455863	455864	455865	455868	455869	455870	455871	455872	455873
mRNA.Accession	NM_001099298	NM_001099298	NM_001099298	NM_001099298	NM_001099298	NM_001099298	NM_001099298	NM_001099298	NM_001099298	NM_001099298	NM_001099298	NM_001099298	NM_001099298	NM_001099298	NM_001099298
Chromosome	chr2	chr2	chr2	chr2	chr2	chr2	chr2	chr2	chr2	chr2	chr2	chr2	chr2	chr2	chr2
Strand	+	+	+	+	+	+	+	+	+	+	+	+	+	+	+
Start	65528108	65528154	65528308	65539811	65545331	65549790	65551755	65553883	65555476	65564394	65566456	65568167	65571294	65573672	65581148
Stop	65528137	65528302	65528373	65539978	65545413	65549894	65551892	65554036	65555763	65564494	65566595	65568336	65571398	65573934	65581172
P30.WT.CSMN	8.8	9.9	9.2	10.5	9.7	8.3	7.4	10.1	11.4	10.2	10.6	11.8	10.2	11.6	11.6
P30.SOD1.CSMN	9.2	10.3	9.5	11	10.2	8.7	8	11.2	12	10.5	10.9	12.3	10.6	12.3	12.2

Exon.ID	455874	455875	455876	455880	455880	455880	455881	455881	455882	455882	455882	455882	455882	455882
mRNA.Accession	NM_001099298	NM_001099298	NM_001099298	NM_001099298	NM_001099298	NM_001099298	NM_001099298	NM_001099298	NM_001099298	NM_001099298	NM_001099298	NM_001099298	NM_001099298	NM_001099298
Chromosome	chr2	chr2	chr2	chr2	chr2	chr2	chr2	chr2	chr2	chr2	chr2	chr2	chr2	chr2
Strand	+	+	+	+	+	+	+	+	+	+	+	+	+	+
Start	65586351	65586907	65589925	65602036	65602640	65602682	65602761	65602846	65602934	65603009	65603068	65603139	65603502	65605264
Stop	65586458	65586933	65590139	65602629	65602667	65602734	65602799	65602877	65602991	65603036	65603138	65603182	65604841	65605452
P30.WT.CSMN	10.9	10.7	8.6	10.1	1.5	9.3	9.6	9.8	9.8	2.8	10.5	9.7	9.4	6
P30.SOD1.CSMN	11.5	11	9.5	10.5	3.3	9.6	10.7	9.6	10.7	3.4	11	10.7	9.9	7.4

Scn9a::TranscriptClusterID:6887374

Exon.ID	490931	490931	490931	490932	490933	490935	490936	490937	490938	490940	490941	490942	490944	490945	490946	490946
mRNA.Accession	NM_018852	NM_018852	NM_001099298	NM_018852	NM_018852	NM_018852	NM_018852	NM_018852	NM_018852	NM_018852	NM_018852	NM_018852	AK051636	AK051636	NM_018852	NM_018852
Chromosome	chr2	chr2	chr2	chr2	chr2	chr2	chr2	chr2	chr2	chr2	chr2	chr2	chr2	chr2	chr2	chr2
Strand	-	-	-	-	-	-	-	-	-	-	-	-	-	-	-	-
Start	66318847	66321651	66322540	66325473	66326040	66331181	66331949	66332671	66339675	66342945	66346602	66353373	66358803	66359995	66364874	66365084
Stop	66321451	66321759	66322599	66325634	66326065	66331267	66331973	66332942	66339786	66343048	66346708	66353458	66359135	66360574	66365055	66365108
P30.WT.CSMN	4.3	3.3	10.7	1.7	5.6	3.2	6.8	6	2.6	6.2	5.5	3.8	6.1	3.7	4.5	2.4
P30.SOD1.CSMN	5.9	5.4	11	3.8	6.6	3.8	8.2	6.8	3.2	5.5	5	6.3	6.7	3.9	4.9	1.5

Exon.ID	490947	490947	490948	490948	490949	490951	490951	490952	490953	490954	490955	490955
mRNA.Accession	ENSMUST00000119700	ENSMUST00000119700	NM_018852	NM_001099298	NM_018852	NM_018852	NM_018852	NM_018852	NM_018852	NM_018852	NM_018852	NM_018852
Chromosome	chr2	chr2	chr2	chr2	chr2	chr2	chr2	chr2	chr2	chr2	chr2	chr2
Strand	-	-	-	-	-	-	-	-	-	-	-	-
Start	66368590	66368650	66371139	66371446	66372181	66374168	66374260	66375336	66378476	66381127	66385044	66385103
Stop	66368625	66368723	66371299	66371471	66372344	66374254	66374322	66375456	66378686	66381360	66385072	66385233
P30.WT.CSMN	2.1	4.2	4	10.2	6	3.2	2	5.6	7.1	5.2	6.7	6.1
P30.SOD1.CSMN	3.5	4.3	5.2	10.6	5.6	2.1	1.7	4.6	6.9	5.6	4.2	4.9

Scn10a::TranscriptClusterID:6999442

Exon.ID	879750	879750	879751	879752	879753	879754	879755	879756	879757	879758	879758	879759	879760	879762	879763	879763	879765	879766	879770
mRNA.Accession	NM_009134	NM_009134	NM_009134	NM_009134	NM_009134	NM_009134	NM_009134	NM_009134	NM_009134	NM_009134	NM_009134	NM_009134	NM_009134	NM_009134	NM_009134	NM_009134	AK135787	NM_009134	NM_009134
Chromosome	chr9	chr9	chr9	chr9	chr9	chr9	chr9	chr9	chr9	chr9	chr9	chr9	chr9	chr9	chr9	chr9	chr9	chr9	chr9
Strand	-	-	-	-	-	-	-	-	-	-	-	-	-	-	-	-	-	-	-
Start	119517605	119518183	119522646	119525818	119526845	119527962	119531899	119533144	119536235	119539230	119539328	119540576	119542902	119544613	119547557	119547884	119556263	119560030	119567524
Stop	119517957	119519045	119522890	119525893	119526925	119527989	119532062	119533248	119536318	119539323	119539356	119540666	119542986	119544911	119547803	119547913	119556563	119560195	119567593
P30.WT.CSMN	3.6	5.5	4.6	7.4	4.9	1.6	2.9	3.7	4.2	3.1	10	6.7	4.4	4.7	4.9	7.1	4.2	4.4	3.3
P30.SOD1.CSMN	5.5	6.1	6.2	6.3	6.2	3.6	6	5.9	5.5	5.4	11.5	8.5	6.8	5.8	6.9	8	6.3	6.7	5

Exon.ID	879772	879773	879774	879775	879776	879777	879781	879782	879783	879784	879786	879788	879788	879791	879792	879793	879794	879794
mRNA.Accession	NM_009134	NM_009134	NM_009134	NM_009134	NM_009134	NM_009134	NM_009134	AK051134	NM_009134	AK051134	NM_009134	NM_009134	NM_009134	DQ497424	DQ497424	DQ497422	DQ516971	DQ516971
Chromosome	chr9	chr9	chr9	chr9	chr9	chr9	chr9	chr9	chr9	chr9	chr9	chr9	chr9	chr9	chr9	chr9	chr9	chr9
Strand	-	-	-	-	-	-	-	-	-	-	-	-	-	-	-	-	-	-
Start	119570298	119573899	119575012	119575488	119579073	119579475	119581263	119596446	119597007	119597370	119600563	119603174	119603449	119625548	119625788	119627936	119628129	119628173
Stop	119570489	119574053	119575194	119575620	119579133	119579635	119581352	119596727	119597066	119598083	119600680	119603426	119603473	119625742	119625848	119628067	119628170	119628279
P30.WT.CSMN	4.3	5.4	4.9	5.4	4.6	4.3	2.4	5.5	3.5	5.3	4.1	5.8	3.7	5.5	6.6	3.9	7.2	3
P30.SOD1.CSMN	6.2	6.6	5.6	5.9	6	7.4	4.3	6.1	3.7	6	5.5	6.6	2.8	7.3	8.2	4.8	8.2	6.1

Exon.ID	490956	490957	490958	490963	490964	490965	490966	490966	490968	490969	490970	490971
mRNA.Accession	NM_018852	NM_018852	NM_018852	NM_018852	NM_018732	NM_001099298	AK141871	NM_018852	NM_018852	NM_018852	NM_018852	NM_018852
Chromosome	chr2	chr2	chr2	chr2	chr2	chr2	chr2	chr2	chr2	chr2	chr2	chr2
Strand	-	-	-	-	-	-	-	-	-	-	-	-
Start	66387061	66388401	66389745	66400446	66401402	66401604	66402840	66403200	66403874	66404231	66406167	66472687
Stop	66387172	66388458	66389773	66400530	66401433	66401668	66403082	66403288	66403906	66404321	66406298	66472962
P30.WT.CSMN	6.1	3.7	4.4	3.6	8.7	5	3.7	5.1	4.4	2.4	4.7	5.5
P30.SOD1.CSMN	6.1	3.3	4.8	3.6	9.8	4	4.3	4.8	4.1	3.9	5.3	6.2

Scn11a::TranscriptClusterID:6999443

Exon.ID	879800	879800	879800	879803	879804	879806	879807	879809	879810	879813	879815	879817	879818	879819	879819	879820	879821	879821
mRNA.Accession	NM_011887	NM_011887	NM_011887	NM_011887	NM_011887	NM_011887	NM_011887	NM_011887	NM_011887	NM_011887	NM_011887	NM_011887	NM_011887	NM_011887	NM_011887	NM_011887	NM_011887	NM_011887
Chromosome	chr9	chr9	chr9	chr9	chr9	chr9	chr9	chr9	chr9	chr9	chr9	chr9	chr9	chr9	chr9	chr9	chr9	chr9
Strand	-	-	-	-	-	-	-	-	-	-	-	-	-	-	-	-	-	-
Start	119662970	119663369	119663641	119667566	119668987	119674556	119677470	119678883	119679543	119683420	119688059	119689898	119691024	119693012	119693235	119699053	119701494	119701542
Stop	119663302	119663448	119664384	119667784	119669011	119674679	119677511	119679138	119679628	119683559	119688128	119690013	119691094	119693180	119693259	119699375	119701522	119701626
P30.WT.CSMN	4.7	5.3	5.9	3.5	1.2	2.3	4.3	5.4	5.5	3.4	4.1	2.2	4	5.2	6.2	4	5	2.9
P30.SOD1.CSMN	6.6	5.6	7.2	6.1	2	4.9	5.6	7	7.1	2.6	6.6	3.3	3.6	5	7.4	6.2	7.1	4.3

Exon.ID	879822	879823	879824	879824	879825	879826	879827	879828	879829	879830	879831	879832	879834	879837	879837	879838
mRNA.Accession	NM_011887	NM_011887	NM_011887	NM_011887	NM_011887	NM_011887	NM_011887	NM_011887	NM_011887	NM_011887	NM_011887	NM_011887	NM_011887	ENSMUST00000070617	ENSMUST00000070617	NM_011887
Chromosome	chr9	chr9	chr9	chr9	chr9	chr9	chr9	chr9	chr9	chr9	chr9	chr9	chr9	chr9	chr9	chr9
Strand	-	-	-	-	-	-	-	-	-	-	-	-	-	-	-	-
Start	119703085	119704780	119712459	119712583	119713566	119714796	119716045	119716869	119720251	119722176	119724326	119725628	119728860	119732889	119732928	119734543
Stop	119703238	119704871	119712524	119712607	119713681	119714911	119716098	119717015	119720303	119722304	119724395	119725737	119729108	119732924	119732990	119734567
P30.WT.CSMN	5.4	4.5	4.1	6	4.4	4.3	4.3	5.7	2.7	2.2	2.8	2.5	4.1	6.2	6	2.9
P30.SOD1.CSMN	5.7	5.8	6.3	4.6	5.8	5.4	3.1	6.8	2.9	3.6	2.8	2.5	6.5	5.9	6.6	5.7

Scn1b::TranscriptClusterID:6966356

Exon.ID	762806	762808	762809	762810
mRNA.Accession	NM_011322	NM_011322	NM_011322	NM_011322
Chromosome	chr7	chr7	chr7	chr7
Strand	-	-	-	-
Start	31902765	31907943	31910097	31911722
Stop	31902856	31908162	31910210	31911990
P30.WT.CSMN	11	11.9	12.2	8
P30.SOD1.CSMN	12.4	12.9	12.9	8.7

Scn1b::TranscriptClusterID:6966355

Exon.ID	762805
mRNA.Accession	NM_011322
Chromosome	chr7
Strand	-
Start	31901578
Stop	31901676
P30.WT.CSMN	7.6
P30.SOD1.CSMN	9.7

Scn2b::TranscriptClusterID:6988713

Exon.ID	839804	839804	839806	839807	839808	839808	839808	839808	839809	839810
mRNA.Accession	NM_001014761	NM_001014761	NM_001014761	NM_001014761	NM_001014761	NM_001014761	NM_001014761	NM_001014761	NM_001014761	NM_001014761
Chromosome	chr9	chr9	chr9	chr9	chr9	chr9	chr9	chr9	chr9	chr9
Strand	+	+	+	+	+	+	+	+	+	+
Start	44925961	44926107	44933066	44933518	44934192	44934359	44934564	44934817	44934963	44936052
Stop	44926104	44926168	44933184	44933710	44934336	44934387	44934753	44934887	44935175	44937409
P30.WT.CSMN	8.4	10.6	9.9	8.3	9.1	9	6.6	7.9	7.3	10.1
P30.SOD1.CSMN	9.6	11.7	10.9	9	10.3	10.4	8.4	8.9	9	11.5

Scn3b::TranscriptClusterID:6988366

Exon.ID	838683	838683	838684	838686	838687	838687	838687	838689	838690	838692	838693	838693	838693	838693	838693
mRNA.Accession	NM_153522	NM_153522	NM_153522	NM_153522	AK051805	AK051805	AK051805	NM_153522	NM_153522	NM_153522	NM_178227	NM_153522	NM_153522	NM_153522	NM_153522
Chromosome	chr9	chr9	chr9	chr9	chr9	chr9	chr9	chr9	chr9	chr9	chr9	chr9	chr9	chr9	chr9
Strand	+	+	+	+	+	+	+	+	+	+	+	+	+	+	+
Start	40076815	40077359	40077578	40084672	40084869	40085224	40085323	40086961	40090027	40095985	40096073	40096129	40096275	40096860	40097432
Stop	40077176	40077523	40077631	40084748	40085038	40085274	40085590	40087154	40090129	40096020	40096127	40096184	40096694	40096943	40099000
P30.WT.CSMN	6.4	4.7	7.7	7.5	5.9	5.3	5.2	8	6.9	8	6.7	8.6	8.4	6.7	6.1
P30.SOD1.CSMN	7.7	4.8	9.5	9.1	6.5	8	7.1	9.3	8.1	8.3	7.4	9.8	9.6	8.1	8.7

Scn4b::TranscriptClusterID:6988714

Exon.ID	839811	839811	839812	839813	839815	839816	839817	839817
mRNA.Accession	ENSMUST00000060125	NM_001013390	NM_001013390	NM_001013390	NM_001013390	NM_001013390	NM_001013390	NM_001013390
Chromosome	chr9	chr9	chr9	chr9	chr9	chr9	chr9	chr9
Strand	+	+	+	+	+	+	+	+
Start	44946546	44947128	44954767	44955763	44957353	44958496	44958618	44961799
Stop	44946999	44947169	44954919	44955922	44957428	44958559	44961193	44962142
P30.WT.CSMN	7.4	9.3	10	8.3	9	7.9	10.1	8.7
P30.SOD1.CSMN	8.4	10.1	10.7	8.5	9.5	8.2	10.9	9.1
