# Supplementary figures and images for: The Electrophysiological Determinants of Corticospinal Motor Neuron Vulnerability in ALS
Source: Front Mol Neurosci. 2020 May 19;13:73. doi: 10.3389/fnmol.2020.00073 (PMC7248374; doi:10.3389/fnmol.2020.00073)

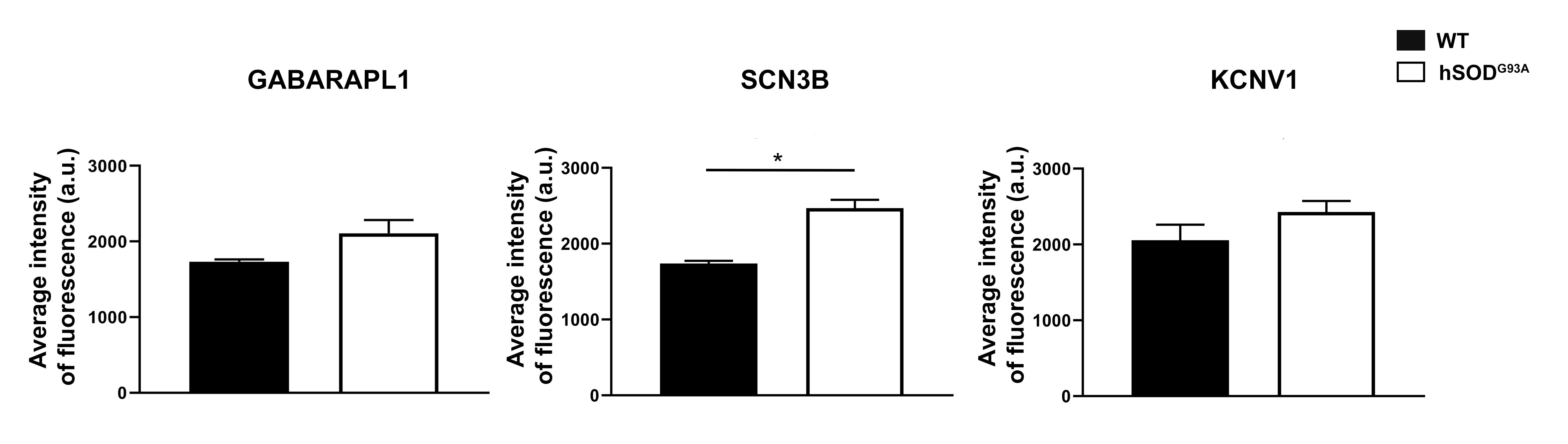

Supplement: FIGURE S2 — Bar graph representation for the average intensity of fluorescence for GABARAPL1, SCN3B, and KCNV1 expression in CSMN of WT (black box) and hSOD1G93A (white box) mice at P30. Data are shown as mean ± SEM of three independent experimental replicates. T-test is used to determine statistical significance, and *p < 0.05 is considered significant. [file Image_2.tiff]
